# Dual-Action NSAID-Gold(I) Alkynyl Hybrids for Synergistic Anti-Inflammatory and Anticancer Therapy of Colorectal Cancer

**DOI:** 10.1021/acs.inorgchem.5c05908

**Published:** 2026-03-27

**Authors:** Javier Sáez, Luis Vicente Herrera-Marcos, María Jesús Rodríguez-Yoldi, M. Concepción Gimeno, Elena Cerrada

**Affiliations:** † Departamento de Química Inorgánica, Instituto de Síntesis Química Y Catálisis Homogénea (ISQCH). Universidad de Zaragoza-C.S.I.C, Zaragoza 50009, Spain; ‡ Departamento de anatomía E Histología Humanas, 16765 Facultad de Ciencias de la Salud Y Del Deporte, Pl. Universidad 3, Huesca 22002, Spain; § Departamento de farmacología Y Fisiología, Medicina Legal Y Forense. Unidad de Fisiología, Facultad de Veterinaria, Ciber de Fisiopatología de la Obesidad Y Nutrición (CIBERobn), Instituto Agroalimentario de Aragón (IA2), Zaragoza 50013, Spain; ∥ Instituto de Investigación Sanitaria de Aragón (IIS Aragón), Zaragoza 50009, Spain

## Abstract

Colorectal cancer (CRC) remains a major global health challenge, in which chronic inflammation and redox dysregulation are key drivers of tumor progression. Here, we report a rationally designed family of NSAID-derived alkyne ligands coordinated to JohnPhos–gold­(I) fragments, affording eight new alkynyl gold­(I) derivatives. Complexes based on naproxen, ibuprofen, and salicylic acid derivatives display potent antiproliferative activity against Caco-2/TC7 colon cancer cells, outperforming oxaliplatin and being comparable to auranofin, while showing markedly reduced cytotoxicity in breast cancer lines and nonmalignant cells, thus indicating promising selectivity. Mechanistic studies revealed that the most active complex, [Au­(L1)­JP] (**1**), which contains a naproxen-derived alkyne, inhibits thioredoxin reductase (TrxR), triggers ROS overproduction, disrupts mitochondrial membrane potential, and induces G1-phase arrest while only marginally increasing apoptosis. This suggests the involvement of additional forms of cell death or cytostatic effects. Additionally, complex **1** selectively inhibits the enzyme cyclooxygenase-2 (COX-2) over COX-1 and reduces *IL-8* expression without affecting *PTGS2* transcription, highlighting a post-transcriptional anti-inflammatory action. These results support NSAID-derived alkynyl gold­(I) complexes as promising multitarget agents for colorectal cancer intervention, combining disruption and COX-2 modulation.

## Graphical abstract

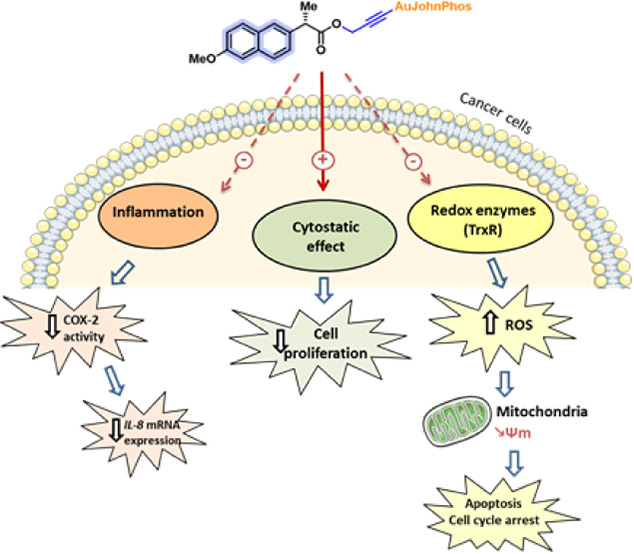

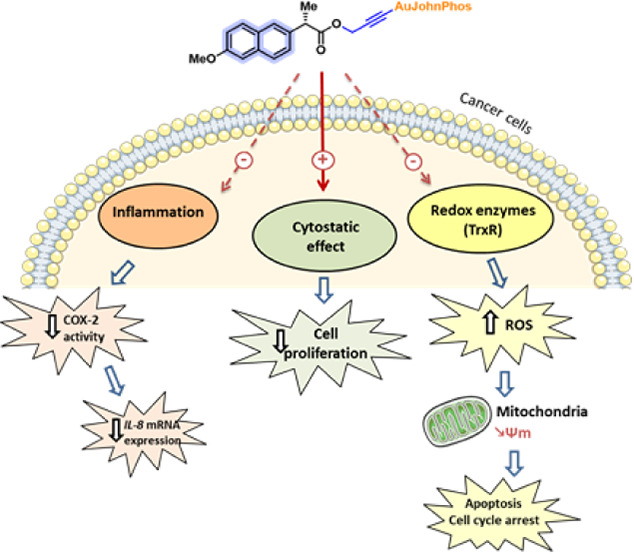

## 1 Introduction

Colorectal cancer (CRC), a disease characterized by abnormal cell growth in the colon and rectum, ranks among the leading causes of cancer-related morbidity and mortality worldwide, significantly contributing to global health burdens. Despite advances in screening and treatment, the development of CRC remains driven by complex interactions among genetic, environmental, and lifestyle factors. Most CRC cases have been associated with environmental factors rather than heritable genetic changes. Risk factors include food-borne and ecological mutagens, specific intestinal microbes and pathogens, and chronic intestinal inflammation, which often precedes tumor development.^1^ These events cause structural changes in DNA, transforming normal cells into cancer cells.^2^

In recent years, inflammation has become a key factor in the development and progression of colorectal cancer. Chronic inflammation, especially within the colonic microenvironment, has been associated with promoting neoplastic transformation through mechanisms such as cellular damage, genomic instability, and the activation of oncogenic pathways.^3^ Tumor progression relies on the interactions between tumor cells and components of the tumor microenvironment. Cells in the microenvironment release pro-inflammatory cytokines, chemokines, growth factors, and proteases that regulate the growth, differentiation, and survival of cancer cells, thereby facilitating angiogenesis and metastasis.^4^ Although chronic inflammation may be involved in all three stages of tumor development (initiation, promotion, and progression) it appears to play a significant role in tumor promotion and progression.^5^

Inflammatory conditions such as inflammatory bowel disease (IBD), which includes Crohn’s disease and ulcerative colitis, are well-recognized risk factors for colorectal cancer, emphasizing the connection between chronic inflammation and tumorigenesis.^6,7^ Cancer-related inflammation accelerates drug resistance, making it a promising target for the prevention, treatment, and control of cancer.^8^ Inflammation is driven by specific prostaglandins and thromboxanes, whose production is catalyzed by the enzyme cyclooxygenase (COX), particularly the inducible isoform COX-2. It is essential to recognize that the COX-2 gene is often overexpressed in various cancerous tissues and transformed cells, including those of colon cancer.^9,10^ Overexpression of COX-2 increases prostaglandin production, which may promote angiogenesis, cell proliferation, and the suppression of apoptosis.^11^ Therefore, COX-2 could be considered a molecular target for colorectal cancer prevention, since blocking its activity has been demonstrated to have antitumor and antiangiogenic effects in a wide range of human malignancies.^12–14^ Regular use of nonsteroidal anti-inflammatory drugs (NSAIDs), especially selective COX-2 inhibitors (COXIBS), has been shown to reduce colorectal adenomas by 30–40% and decrease CRC risk.^15,16^ However, despite their proven chemopreventive benefits, long-term NSAID use is not advised clinically due to significant adverse effects. Traditional NSAIDs such as aspirin, ibuprofen, and naproxen, which nonselectively inhibit both COX-1 and COX-2, cause gastrointestinal toxicity (ulcers, bleeding) by suppressing COX-1-mediated gastroprotective prostaglandin synthesis.^17^ While selective COX-2 inhibitors (e.g., celecoxib) avoid gastrointestinal problems, they carry increased cardiovascular risks, including myocardial infarction and stroke, especially with prolonged use. This therapeutic dilemma has prompted the search for novel alternative strategies that maintain COX-2 inhibition while reducing systemic toxicity.

Given the limitations of NSAIDs as chemopreventive agents, alternative strategies that combine anti-inflammatory and cytotoxic mechanisms are needed. In this context, gold complexes have garnered significant interest as promising alternatives to conventional chemotherapy, particularly in the treatment of colon cancer.^18,19^ Unlike commonly used platinum-based drugs like cisplatin, which often cause severe side effects, gold complexes operate through different mechanisms. They may selectively target cancer cells while reducing toxicity to healthy tissue. Among these, gold alkynyl derivatives are highly versatile compounds with applications spanning cancer therapy, diagnostics, catalysis, and materials science.^20–24^ Their unique attributes, such as inhibiting key enzymes like thioredoxin reductase (TrxR), which can lead to oxidative stress, and their notable photophysical properties, have unlocked therapeutic and technological opportunities.

Herein, we report the synthesis of new NSAID-derived ligands bearing a propargyl ether group, along with their corresponding phosphine gold­(I) derivatives, and evaluate their activity against a panel of cancer cell lines. To support the hypothesis of a multitarget profile, we investigated their effects on the redox enzyme thioredoxin reductase and assessed their selectivity for the COX-1 and COX-2 isoforms. Additionally, their anti-inflammatory properties were evaluated by analyzing their impact on the mRNA expression levels of *IL-8*, *NOS2*, and *PTGS2*.

## 2 Results and Discussion

### 2.1 Synthesis of the Ligands

The synthesis of the new ligands **L1–6** (Scheme 1), derived from NSAIDs, was achieved using two distinct strategies. For naproxen, ibuprofen, mefenamic acid, and indomethacin, the propargyl group was introduced in a single step (Scheme 1A,i) by reacting the carboxylic acid with propargyl bromide in refluxing acetone, using K_2_CO_3_ as the base. The corresponding ligands **L1–L4** were obtained in good yields (around 70%). However, for salicylic acid and diflunisal, an alternative approach was necessary (Scheme 1A,ii), since the same conditions produced a mixture of mono- and dialkylated derivatives. In this case, propargylation was performed with tetrabutylammonium fluoride (TBAF) in THF, allowing the isolation of pure ligands **L5** and **L6** after column chromatography (yields: 55–65%).

**Scheme 1:**
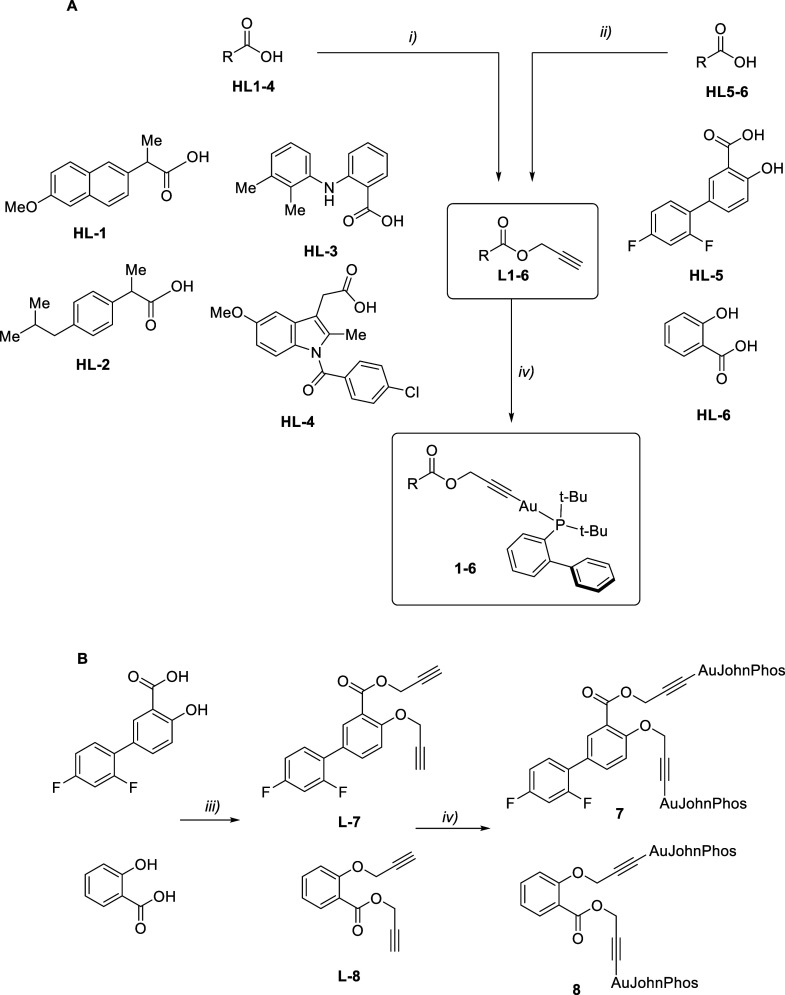
Synthetic Strategy for the Preparation of NSAID Alkyne L1-8 and the Corresponding Mononuclear Gold­(I) Complexes **1**–**6** (A) and Dinuclear **7**–**8** (B)^a^

a i) Propargyl bromide + K_2_CO_3_; (ii) Propargyl bromide + TBAF; (iii) Propargyl bromide (3.2 equiv) + K_2_CO_3_; (iv) [Au­(acac)­(JP)]

The formation of the new ligands was confirmed by ^1^H NMR spectroscopy. A characteristic doublet for the methylene group was observed at around 5 ppm. In the ^1^H NMR spectra of naproxen and ibuprofen, the signal corresponding to the methylene group adjacent to the stereogenic center appears as a doublet of doublets of doublets (ddd). The presence of the chiral carbon creates an anisotropic environment, making the diastereotopic methylene protons magnetically nonequivalent. This nonequivalence results in geminal coupling (J_H–H_) between the two protons, along with the expected vicinal couplings (J_H–H_) with nearby nuclei. The combination of these interactions explains the complex multiplicity observed in the signal. The disappearance of the broad carboxylic acid resonance around 12 ppm further confirms the conversion of the parent NSAIDs into their propargyl derivatives. Additionally, a triplet at 2.5 ppm (J_H–H_ ≈ 2.4 Hz) confirms the presence of the acetylenic proton.

Additional evidence was obtained from their IR spectra, which showed the disappearance of the strong O–H stretching band of the carboxylic acid (2500–3300 cm^–1^) and the C=O stretching band associated with the COOH group, along with the appearance of the characteristic C≡C stretching vibration of the propargyl fragment (2100–2200 cm^–1^). These features collectively confirmed the successful propargylation of all ligands.

The synthesis of the bis-alkyne ligands **L7–8**, also derived from diflunisal and salicylic acid, was carried out using 3.2 equiv of propargyl bromide in the presence of K_2_CO_3_ (Scheme 1B,iii). In this case, the ^1^H NMR spectra confirmed the substitution at both positions, as evidenced by two doublets around 4.7 ppm and two triplets near 2.4 ppm (*J*_H–H_ ≈ 2.4 Hz).

### 2.2 Synthesis of Gold Complexes

Treatment of the alkyne ligands **L1–6** with [Au­(acac)­(JP)] (JP = JohnPhos, acac = acetylacetonate) yielded the corresponding alkynyl–phosphane gold­(I) derivatives **1–6** (Scheme 1A,iv) as air-stable solids, through deprotonation of the alkyne by the internal base acac. The disappearance of the acetylenic triplet and the downfield shift of the methylene resonance in their ^1^H NMR spectra clearly indicate gold coordination. The spectra of complexes **1**–**6** display more complex patterns compared to their precursors, owing to the presence of the JohnPhos ligand in the aromatic region. The ^31^P­{^1^H} NMR spectra exhibit a single resonance at approximately δ 64.3 ppm, consistent with a unique phosphorus environment. The bis-alkyne ligands **L7–8** afford complexes bearing two Au­(I)–JohnPhos units, as evidenced by the appearance of two distinct phosphorus resonances in their ^31^P­{^1^H} NMR spectra. Furthermore, the IR spectra of the complexes show the disappearance of the ≡C–H stretching band at approximately 3250 cm^–1^, confirming deprotonation of the terminal alkyne. In contrast, the ν­(C≡C) stretching vibration, observed for the free ligands at around 2100–2120 cm^–1^, does not exhibit a significant shift upon coordination.^25^

The crystalline structure of complex **3**, derived from the NSAID mefenamic acid, has been established by X-ray analysis (Figure 1).

**Figure 1:**
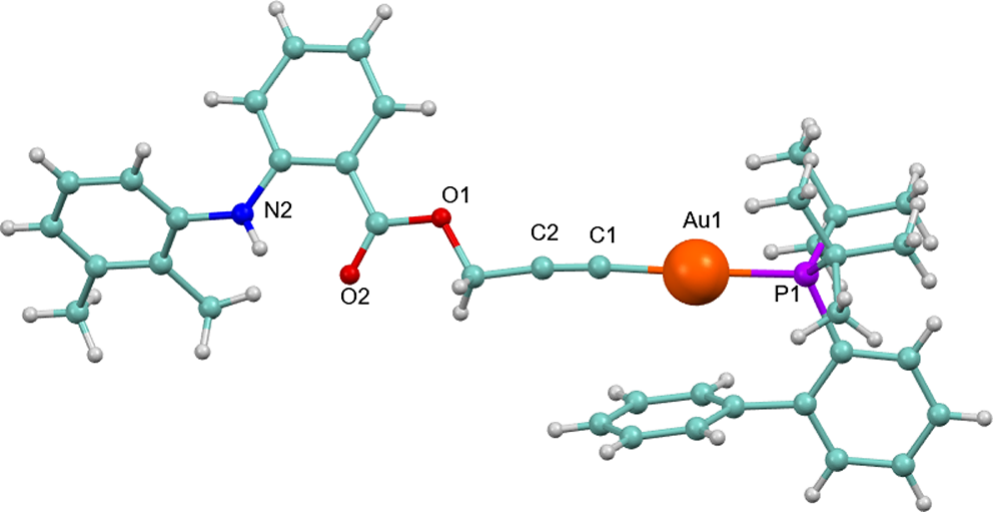
Molecular structure of complex **3**. Selected bond lengths [Å] and angles [°]: Au1–C1 2.016(3), Au1–P1 2.2939(7), O1–C4 1.347(3), O1–C3 1.459(3), C1–C2 1.185(4), O2–C4 1.217(3), N2–C10 1.375(3), N2–C11 1.423(3); C1–Au1–P1 177.83(8), C2–C1–Au1 175.4(2), C1–C2–C3 173.3(3).

The complex crystallizes in the triclinic P-1 space group with one molecule in the asymmetric unit. The gold center is coordinated to the alkynyl carbon and the phosphorus atom, with bond distances of Au1–C1 = 2.016(3) Å and Au1–P1 = 2.2939(7) Å, respectively. The metal adopts the expected linear geometry, defined by a C–Au–P angle of 177.83(8)°, which is consistent with the nearly colinear arrangement of the alkynyl fragment (C2–C1–Au1 = 175.4(2)°). Bond lengths and angles within the alkynyl unit and within the carboxylate group of the mefenamic moiety fall within normal ranges. An intramolecular N2–H···O2 hydrogen bond (1.995 Å) is present, along with a slipped Au···π interaction of approximately 3 Å involving the phenyl ring of the JohnPhos ligand.

### 2.3 Solution Stability

The stability of the gold­(I) complexes under physiologically relevant conditions was evaluated by UV–visible absorption spectroscopy in phosphate-buffered saline (PBS, pH 7.4) at 37 °C. Stock solutions (10 mM) were prepared in DMSO and diluted to working concentrations in PBS. Monitoring through UV–vis spectroscopy over 72 h (Figure S87) confirmed that no absorption band around 500 nm (indicative of colloidal gold formation via reduction of Au­(I) to Au(0)) was detected for any complex at any time point, demonstrating the absence of metal degradation under these conditions. However, the lipophilic nature of the complexes (see Table 1), imposed by the bulky JohnPhos phosphane ligand, resulted in limited aqueous solubility at concentrations exceeding 50 μM in both buffered saline and serum-free cell culture media. This behavior, involving the aggregation and/or precipitation of lipophilic gold­(I)-phosphine complexes in aqueous media, has been widely reported for similar systems and is attributed to the hydrophobic character of bulky tertiary phosphane ligands.^26^ Significantly, this solubility limitation does not hinder the biological evaluation of these complexes. The most active derivatives (complexes **1**, **2**, and **6**) exhibit IC_50_ values of 1.5–2 μM against Caco-2 cells (see Table 1), concentrations that are 25–30 times below the solubility threshold observed at 50 μM. This significant margin ensures that the antiproliferative activity was assessed under conditions where the complexes remained in solution, a characteristic shared by other potent lipophilic metallodrugs.

**Table 1:** Distribution Coefficients and IC_50_ (μM)^a^ Values of the Complexes on Caco-2/TC7, MCF-7 and MDA-MB-231 Cancer Cell Lines After 72 h Incubation Compared with Auranofin and Oxaliplatin^b^,^c^

Compound	LogP_7.4_	Caco-2/TC7	MCF-7	MDA-MB231	Fibroblasts	SI
[Au(L1)JP] (**1**)	1.9	1.5 ± 0.2	15.2 ± 0.6	22.6 ± 0.6	8.4 ± 0.1	5.6
[Au(L2)JP] (**2**)	1.7	2.0 ± 0.4	11.5 ±0.8	5.5 ± 0.2	10.0±0.6	4.9
[Au(L3)JP] (**3**)	1.3	22.7 ± 1.5	33.9 ± 2.4	61.8 ± 0.7		
[Au(L4)JP] (**4**)	2.1	28.7 ± 0.6	>100	37.2 ± 1.6		
[Au(L5)JP] (**5**)	1.5	39.1 ± 1.0	18.5 ± 0.9	45.4 ± 0.5		
[Au(L6)JP] (**6**)	1.8	1.6 ± 0.1	11.2 ± 0.4	9.0 ± 0.2	5.4± 0.1	3.3
[Au_2_(L7)(JP)_2_] (**7**)	1.0	5.3 ± 0.2	11.7 ± 0.8	9.0 ± 0.3		
[Au_2_(L8)(JP)_2_] (**8**)	1.6	5.8± 0.0	18.0 ± 0.4	10.4 ± 0.2		
Auranofin	1.6^[b]^	1.8 ± 0.1	3.8 ± 0.4	0.4 ± 0.0		
Oxaliplatin	–1.6^[c]^	7.8 ± 0.4	8.9 ± 0.6	11.0 ± 0.6		

a Mean ± SE of at least three determinations by using the MTT method.

b Ref 31.

c Ref 32.

### 2.4 Lipophilicity

Lipophilicity is a vital parameter in drug development. It can be evaluated using the octanol–water partition coefficient (logP). LogP values above 3 typically indicate lipophilic compounds, while values below 1 suggest hydrophilic ones. In drug development, a compound should neither be excessively hydrophilic nor overly lipophilic. If the compound is too polar, it cannot cross lipid membranes and therefore cannot enter the cell. Conversely, if it is too lipophilic, it may become trapped within the lipid membrane and be unable to pass through this barrier.

Thus, a compound’s lipophilicity affects pharmacokinetics, pharmacodynamics, and bioavailability. Partition coefficients (logP) for complexes **1**–**8** were measured using the shake-flask method. The logP values ranged from 0.96 to 2.09 (Table 1). Most complexes displayed values between 1.3 and 2.1, indicating the lipophilic nature provided by the phosphine ligands (PPh_3_ or JohnPhos). Dinuclear complexes **7** and **8** showed the lowest logP values (0.96 and 1.65, respectively), likely due to the presence of two gold centers that increase molecular polarity. Complex **4**, derived from indomethacin, exhibited the highest logP value (2.09), consistent with the multicyclic and highly conjugated structure of this ligand. These values are comparable to those reported for other gold­(I) complexes with phosphine ligands, such as auranofin (logP = 1.60).

### 2.5 Analysis of the Viability Effect

The effect of the novel alkynyl derivatives on the cellular viability of colon cancer cells (Caco-2/TC7) was assessed using the MTT assay after 72 h of treatment (Table 1). The results were compared with auranofin, a gold-based reference drug, and oxaliplatin, one of the drugs used in first-line chemotherapy for colon cancer (the FOLFOX regimen, which includes leucovorin (folic acid), 5-fluorouracil, and oxaliplatin).^27^ Gold complexes with naproxen, ibuprofen, and salicylic acid-derived alkynes [Au­(L1)­JP] (**1**), [Au­(L2)­JP] (**2**), and [Au­(L6)­JP] (**6**) showed the highest activity, with IC_50_ values between 1.5 and 2 μM- values close to that of auranofin (1.8 μM) and significantly more potent than oxaliplatin (7.77 μM). However, complexes [Au­(L3)­JP] (**3**), [Au­(L4)­JP] (**4**), and [Au­(L5)­JP] (**5**), which contain alkynes derived from mefenamic acid, indomethacin, and diflunisal, respectively, exhibited much weaker activity (>20 μM). This suggests that modifications in the alkyne ligand structure influence potency against colon cancer. Overall, similar IC_50_ values have been previously reported for alkynyl phosphine gold­(I) derivatives in Caco-2 cells.^23,28–30^

The cytotoxicity of the gold derivatives was also evaluated on two breast cancer cell lines: MCF-7, which represents early stage breast cancer with low metastatic potential, and MDA-MB-231 cells, a triple-negative breast cancer model, characterized by high aggressiveness, poor prognosis, and serving as a model of late-stage disease.

Overall, the gold complexes exhibit moderate to low cytotoxicity toward breast cancer cells in comparison with other alkynyl gold­(I) derivatives previously reported that displayed higher cytotoxicity against the same cancer cell lines.^24,33–36^ Auranofin consistently proves to be more potent than most of the new derivatives, especially in MCF-7 cells, with IC_50_ values generally above 10 μM for the tested derivatives. Only the complexes [Au­(L2)­JP] (**2**), [Au­(L6)­JP] (**6**) and [Au_2_(L7)­(JP)_2_] (**7**) show values in the same range as oxaliplatin. In the more aggressive breast cancer cell line MDA-MB-231, complexes [Au­(L2)­JP] (**2**), [Au­(L6)­JP] (**6**) and [Au_2_(L7)­(JP)_2_] (**7**) achieve comparable or even superior cytoxicity (IC_50_ values around 5–9 μM) relative to oxaliplatin, both of which showed similar potency. Based on these data, a certain degree of selectivity toward colon cancer cells can indeed be concluded for some of these alkynyl derivatives. Notably, complexes **1**, **2**, and **6** stand out because they exhibit low IC_50_ values against Caco-2 cells (1.5–2 μM), but show markedly weaker activity against breast cancer models (MCF-7: ∼11–15 μM; MDA-MB-231: ∼9–23 μM). This differential potency suggests a preference for cytotoxicity against colon cancer, in contrast to auranofin, which is broadly active against all tested cell lines, and oxaliplatin, which exhibits only moderate activity and lacks marked selectivity.

In addition to the complexes, the free ligands were also evaluated against the three cell lines. No relevant cytotoxicity was observed, as evidenced by IC_50_ values above 50 μM in all cases (Table S1)

There is no linear correlation between lipophilicity (expressed as logP) and cytotoxicity. However, most complexes within an intermediate range (logP 0.96–1.9) exhibit the lowest IC_50_ values, suggesting an optimal lipophilicity window that results in a more efficient reduction of cell viability.

Additionally, we tested the toxicity of the most active gold derivatives [Au­(L1)­JP] (**1**), [Au­(L2)­JP] (**2**), and [Au­(L6)­JP] (**6**) on fibroblast cells to obtain an initial assessment of their effects on noncancerous tissue (Table 1). The Selectivity Index (SI), calculated as SI = IC_50_(fibroblasts)/IC_50_(Caco-2), afforded similar values for the three complexes, although higher selectivity toward cancer cells is observed for complexes **1** and **2**.

### 2.6 Interaction with Biomolecules

#### 2.6.1 Interaction with NAC and GSH

Within biological systems, gold complexes encounter high concentrations of thiol-containing biomolecules, such as glutathione (GSH, millimolar levels in cells) and cysteine-rich proteins (e.g., serum albumin), which can interact with the metal center due to gold’s strong affinity for sulfur-containing soft bases.^37–39^ To assess the stability of the alkynyl gold complexes under these conditions, N-acetyl-l-cysteine (NAC) and reduced glutathione (GSH) were selected as representative model nucleophiles. The reactivity of the most active complexes **1**, **2**, and **6** toward equimolar amounts (10 mM) of NAC or GSH was monitored in DMSO-*d*_6_/D_2_O (80:20, v/v) at room temperature by ^1^ H and ^31^ P­{^1^H} NMR spectroscopy over 72 h at 37 °C. ^1^ H NMR and ^31^P­{^1^H} NMR monitoring (Figures S61–S73) showed spectral changes consistent with ligand exchange (see Scheme 2 as an example of the reactivity of complex **1**).

**Scheme 2:**
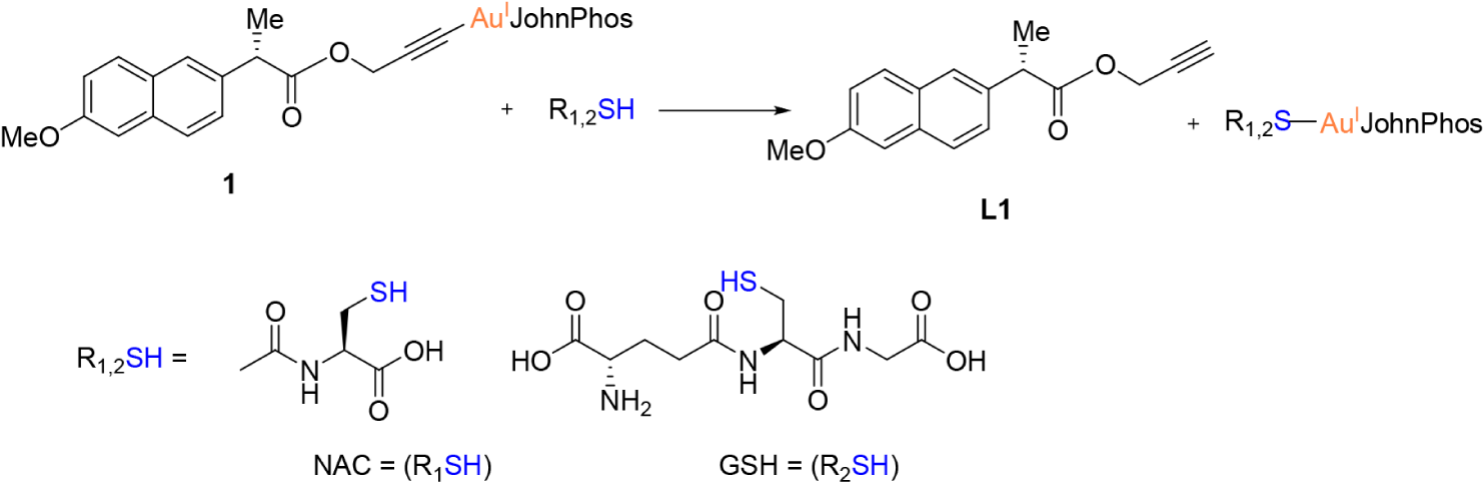
Reaction Scheme between [AuL1­(JP)] (1) (JP = JohnPhos) and NAC/GSH

Notably, modifications were observed in two regions common to all three complexes upon reaction with both NAC and GSH: the shift of signals around 5 ppm and the appearance of a characteristic triplet near 3.5 ppm. Although the complexity of the reaction mixture and spectral overlap prevented definitive assignment of all individual resonances, the observed changes are consistent with the formation of new species resulting from alkynyl displacement (Scheme 2).

Furthermore, signals indicating thiol auto-oxidation were observed in the ^1^H NMR spectra, likely due to disulfide formation (*N*,*N*′-diacetylcystine from NAC, and GSSG from GSH) under the mildly aerobic conditions of the NMR experiments. However, the extent of auto-oxidation could not be quantified due to spectral overlap with signals from the Au-thiolate species and the liberated alkyne. ^31^P­{^1^H} NMR spectroscopy provided unambiguous confirmation of ligand exchange. The signal corresponding to the parent alkynyl–Au­(I) complexes (δ 64.05 ppm in the case of complex **1**) was progressively replaced by a new resonance at δ 63.38 ppm (see Figure S61), assigned to the corresponding thiolate–Au­(JP) species based on observed ligand substitution. The slight upfield shift upon coordination to the thiolate ligand aligns with increased electron density at gold from the more electron-donating sulfur donor relative to the π-acidic alkynyl group. After 72 h, the conversion was essentially complete for all three complexes with both NAC and GSH, confirming that thiol nucleophiles readily displace the alkynyl ligands under these conditions (Figure S61 and Scheme 2). These results demonstrate that complexes **1**, **2**, and **6** undergo ligand exchange with thiol-containing biomolecules, generating stable Au­(I)–thiolate species and releasing the free propargylated NSAID. This reactivity is consistent with that reported for other gold­(I) complexes and suggests that coordination to cysteine residues in target proteins, such as thioredoxin reductase (see below), may play a key role in their biological activity.

The use of phosphine ligands in medicinal inorganic chemistry might induce decomposition through dissociation of the phosphine and be followed by oxidation in the biological medium. Such dissociation and subsequent oxidation have been observed in auranofin^40^ and related phosphine gold­(I) derivatives,^41,42^ although no decomposition has been detected in such examples. In our case, solution studies in the presence of thiol-containing biomolecules show no evidence of phosphine dissociation, probably due to the stronger bond to the gold center and the bulky nature of JohnPhos.

#### 2.6.2 Interaction with BSA

In intravenous administration of chemotherapeutic agents, bovine serum albumin (BSA) and human serum albumin (HSA), among other serum proteins, play vital roles in the pharmacokinetics and pharmacodynamics of therapeutic molecules. Additionally, they significantly influence toxicity, biodistribution, and metabolism, thereby enhancing drug efficacy. These proteins are abundant in the bloodstream and can serve dual functions in drug interactions, both activating drugs and shielding them from degradation. Both BSA and HSA display strong ligand-binding capacities, supported by their multiple binding pockets and a rich network of noncovalent interactions, including hydrogen bonding, hydrophobic contacts, van der Waals forces, and electrostatic contributions. This binding can notably affect the solubility, stability, distribution, and elimination of drugs in the body.

The interaction of the most biologically active alkynyl gold derivatives [Au­(L1)­JP] (**1**), [Au­(L2)­JP] (**2**) and [Au­(L6)­JP] (**6**) with alkyne ligands derived from the NSAIDs naproxen, ibuprofen and salicylic acid and BSA was investigated by fluorescence spectroscopy. The complexes were gradually added to a BSA solution, and the quenching of BSA’s intrinsic fluorescence, attributed to its tryptophan residues, was monitored upon excitation at 295 nm. The naproxen-derived complex (**1**) exhibited fluorescence emission in the 310–400 nm range and was therefore excluded from the study.

During the fluorescence titration experiments, the BSA concentration was kept at 50 μM, while the concentration of the gold complex varied. The tryptophan residue in BSA was excited at 290 nm, and emission was measured between 300 and 450 nm. A concentration-dependent quenching of fluorescence was observed, with no significant changes in shape, and a slight shift in the maximum position (approximately 6–8 nm) was noted (see Figure 2 as an example), indicating subtle changes in the environment surrounding the tryptophan residues.

**Figure 2:**
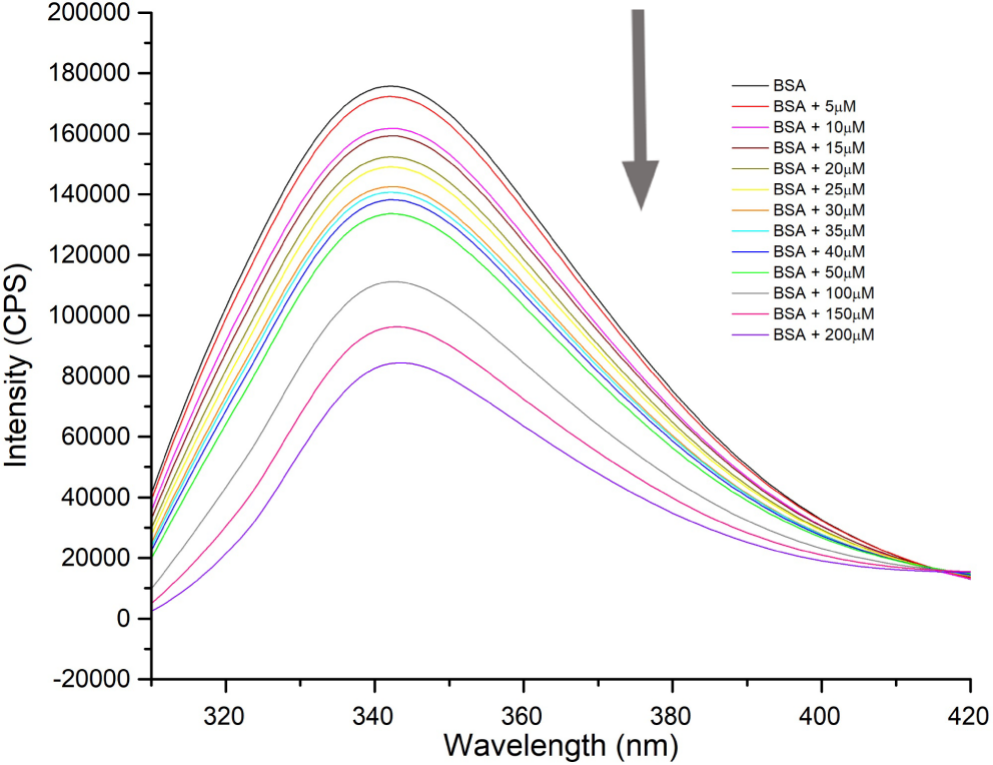
Fluorescence quenching of BSA at 298 K (λ_exc_ = 290 nm, [BSA] = 50 μM) in the presence of increasing concentrations of [Au­(L6)­JP] (6), arrow indicates the increase of the quencher concentration (0–200 μM).

Fluorescence data were analyzed using the Stern–Volmer equation, and the corresponding plot of F_0_/F versus [complex] (F and F_0_ are the corresponding intensities in the presence and the absence of the quencher agent) gives a linear plot (Figures S76 and S78), characteristic of the presence of a single mechanism of quenching, either static or dynamic.^43^ This plot was used to determine the Stern–Volmer quenching constant *K*_sv_ (Table 2). The complex with NSAID ibuprofen (**2**) requires a higher concentration to quench the emission (Figure S74), resulting in a significantly lower quenching constant (Figure S76) than that of the salicylic acid complex (**6**).

**Table 2:** Values of Stern–Volmer Quenching Constant (*K_sv_*), the Number of Binding Sites and the Apparent Binding Constant (*K_b_*) for the Interaction of Complexes **2** and **6** with BSA and with BSA-Ibuprofen or BSA-Phenylbutazone for Complex **6**

	*K*_*sv*_ (M^–1^)	*K*_*b*_ (M^–1^)	*n*
BSA-[Au(L2)(JP)] (**2**)	955.28	1.07 × 10^4^	1.27
BSA-[Au(L6)(JP)] (**6**)	7386.1	3.28 × 10^5^	1.36
BSA-Ibuprofen-**6**	5660.8	3.01 × 10^5^	1.38
BSA-Phenylbutazone-**6**	4930.8	1.07 × 10^4^	1.27

The representation of log (F_0_ – F/F) versus log­[complex] (Figures S75 and S79) allowed us to calculate the binding constant for each experiment and the number of binding sites. The binding constants (summarized in Table 2) are in the same order as other alkynyl gold complexes previously described by some of us.^28,30^

The binding site of the salicylic acid-derived complex (**6**) in BSA was also investigated. The main binding sites of BSA are located in subdomains IIA and IIIA, commonly referred to as sites I and II. Site I preferably accommodates molecules such as warfarin and phenylbutazone; meanwhile, site II is well-suited for ibuprofen, diazepam, and similar molecules.^44–46^ To identify the binding site of complex **6**, displacement experiments were conducted using ibuprofen and phenylbutazone as site markers (Figures S80–S85). The binding constant *K*_*b*_ of complex **6** (Table 2) was significantly reduced in the presence of phenylbutazone but remained unaffected in the presence of ibuprofen, indicating that the complex binds to site I (subdomain IIA) of BSA, which is also the binding site for phenylbutazone.

UV–vis absorption measurement is a straightforward and effective method for detecting interactions with biomolecules, such as BSA. Any changes in the absorption spectrum of BSA could indicate the formation of a metal-BSA adduct. BSA displays two absorption peaks at around 210 nm, corresponding to the α-helix content in the protein, and at 280 nm due to the π → π* transition of aromatic amino acid residues.^47^ The interaction of the complex with naproxen (**1**), which exhibited fluorescence emission in the 310–400 nm range, and BSA was examined at room temperature by UV–vis spectrometry over time and at different concentrations (Figure S86). Increasing amounts of the gold complex added to a solution of BSA (10 μM in PBS) resulted in a slight decrease in absorbance at 280 nm, suggesting a weak interaction between the complex and the biomolecule.

### 2.7 Mechanism Studies

Based on the results obtained regarding the antiproliferative effect and selectivity on cancer cells, [Au­(L1)­JP] (**1**), which contains the naproxen-derived alkyne, was identified as the most promising complex. Its mechanism of action on Caco-2/TC7 cells was then further investigated.

#### 2.7.1 Analysis of the Redox Enzyme TrxR and Determination of ROS Levels

Thioredoxin reductase (TrxR) is a vital enzyme in maintaining cellular redox homeostasis, playing a central role in regulating mitochondrial function and oxidative stress signaling.^48^ In cancer cells, maintaining a balanced redox homeostasis is crucial for cell survival, and disruptions, such as the overproduction of reactive oxygen species (ROS), can trigger cell death.^49^ Therefore, the key regulators of redox balance are attractive targets for anticancer therapy. TrxR is frequently overexpressed in various cancer types, including those from colon cancer.^50,51^ This overexpression is believed to contribute to tumor progression by preserving redox balance and protecting cells against oxidative stress. Consequently, inhibition of this enzyme’s activity has become a promising therapeutic strategy.^52–54^

Gold-based complexes serve as potent inhibitors of TrxR.^55–57^ Its inhibition disrupts the Trx system, impairing antioxidant defenses and increasing intracellular reactive oxygen species (ROS). Mitochondria are known to generate significant amounts of endogenous ROS, especially in cancer cells, which contribute to intracellular oxidative stress.^58^ Generally, gold derivatives exert their anticancer effects by inhibiting TrxR, disrupting mitochondrial function, and inducing oxidative stress (ROS), ultimately leading to apoptosis or other forms of cell death.^59^

Therefore, we investigated the effect of [Au­(L1)­JP] (**1**) on TrxR activity (Figure 3A) and measured ROS production (Figure 3B) in Caco-2/TC7 cells after 24 h of incubation with the complex at its IC_50_ concentration. We evaluated the enzyme activity in protein extracts from Caco-2/TC7 cells treated with the complex for 24 h. The intracellular TrxR activity was determined using a thioredoxin reductase colorimetric assay kit, which detects the reduction of DTNB (5,5′-dithio-bis­(2-dinitrobenzoic acid); Ellman’s reagent) by the enzyme and NADPH to TNB (5-thio-2-nitrobenzoic acid) at 405 nm. Figure 3A shows TrxR activity of 56 ± 9%, indicating an inhibition of 44%. Furthermore, the ability of complex **1** to increase total basal ROS production in Caco-2/TC7 cells was assessed using flow cytometry with the CellROX assay kit. Figure 3B displays an increase in luminescent intensity, suggesting enhanced reactive species generation. Consequently, the tested complex **1** appears to inhibit cellular TrxR activity and target mitochondria, both of which induce significant ROS production, potentially leading to cell death.

**Figure 3:**
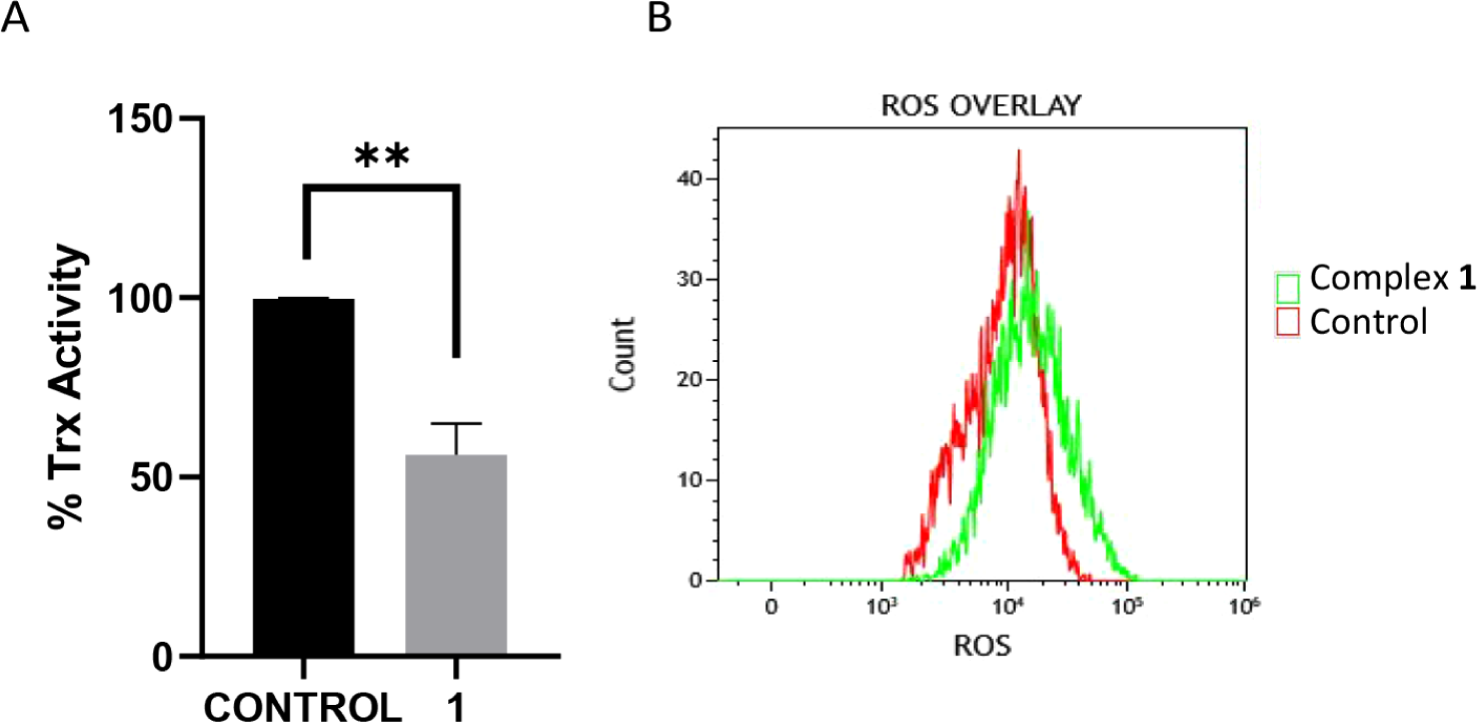
Measurement of the activity of TrxR from Caco-2/TC7 lysates (A) and ROS generation (B) on undifferentiated Caco-2/TC7 cells upon incubation with [Au­(L1)­JP] (**1**) at its IC_50_ value for 24 h. **p < 0.01 vs negative control.

#### 2.7.2 Measurement of COX-1/2 Activity

Prostaglandin-endoperoxide synthase (PTGS), also known as cyclooxygenase, is the key enzyme in prostaglandin biosynthesis. The inducible isoform COX-2 converts arachidonic acid into prostaglandins, particularly prostaglandin E2 (PGE2), during inflammation and has been linked to the growth and development of various human cancers.^60^ As stated above, increased COX-2 expression has been observed in colon cancer tissues from patients with clinically diagnosed colorectal cancer^9,61^ and in Caco-2 cells.^62^

COX-1 is constitutively expressed in most tissues and is crucial for the production of prostaglandins (PGs), which regulate normal physiological functions, making it primarily responsible for “housekeeping” PG synthesis. In contrast, COX-2 is undetectable in most healthy tissues, except for the central nervous system, kidneys, and seminal vesicles; however, it is upregulated by inflammatory and mitogenic stimuli.^63,64^

Using COX-2 inhibitors can slow inflammation and cancer progression while reducing side effects associated with COX-1 inhibition, such as gastrointestinal damage and renal impairment.

To date, only a limited number of gold complexes have been studied for their ability to inhibit COX-2 or alter its gene expression.^28,65–68^ Auranofin was reported to inhibit COX-2, thereby reducing the production of the pro-inflammatory mediator prostaglandin E2.^69^ In contrast, COX-1 inhibition in human platelets needs high concentrations (10 and 100 μM) that, according to our data on differentiated Caco-2 cells, would be considerably more cytotoxic to noncancerous tissues.^70^

We therefore, evaluated the activity of COX-1 (Figure 4A) and COX-2 (Figure 4B) in Caco-2/TC7 cells after 24 h of incubation with the most active complex, which contains an alkyne derived from naproxen [Au­(L1)­JP] (**1**), and compared it with the COX activity in the presence of the free ligand at the same concentration. Both figures display the inhibition of COX enzymes expressed as the percentage of inhibition relative to control. The free ligand is not able to reduce the activity of both COX-1 and COX-2 at this concentration. In contrast, after treatment with the naproxen-derived complex **1**, a moderate COX-2 inhibition of 55% was observed, with a residual activity of 45 ± 14%. In comparison, the inhibition of COX-1 was slight (residual activity: 85 ± 8%), suggesting a specific selectivity toward COX-2. This result is exciting from a therapeutic perspective since COX-2 inhibition plays a central role in controlling inflammation associated with cancer progression, and COX-1 inhibition is associated with gastrointestinal toxicity. Thus, selective inhibition of COX-2 by complex **1** offers a promising strategy for cancer treatment.^71^

**Figure 4:**
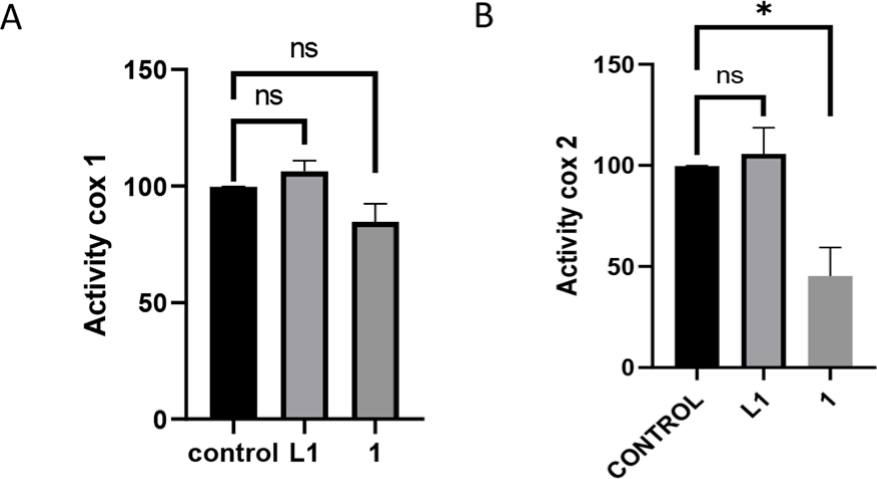
Determination of COX-1 activity (A) and COX-2 activity (B) from undifferentiated Caco-2/TC7 cells after 24 h incubation with [Au­(L1)­JP] (**1**) at its IC_50_ value or incubation with **L1** at the same concentration. *p < 0.05 vs negative control.

Besides, previous studies have shown that the use of selective COX-2 inhibitors such as celecoxib increases mitochondrial superoxide production, leading to ROS-dependent apoptosis in metastatic cancer cells^72^ or generates, elevated ROS in the absence of apoptosis in cutaneous squamous cell carcinoma (cSCC),^73^ which is in accordance with the results covered in Figure 3B.

#### 2.7.3 Anti-Inflammatory Effect of Gold Complexes on Colon Cancer

Nitric oxide (NO) is a short-lived signaling molecule essential for many physiological processes and is produced by nitric oxide synthases (NOS). Due to its mutagenic properties, prolonged exposure to high NO levels from inducible nitric oxide synthase (iNOS) induction during chronic inflammation may contribute to carcinogenesis. NO can promote tumor growth and metastasis by enhancing the migratory, invasive, and angiogenic properties of tumor cells, potentially through the activation of cyclooxygenase (COX-2).^74^

Furthermore, interleukins (ILs) are cytokines that regulate growth, differentiation, and activation in immune and inflammatory responses by binding to high-affinity cell-surface receptors.^75^ IL-8 has been linked to various cancers, including gastric and colorectal cancer, where it is overexpressed in CRC tissues and produced differently by tumor or stromal components depending on the CRC genetic background.^76^ IL-8 plays a crucial role in the progression and metastasis of colon cancer by fostering an inflammatory environment that supports tumor development, angiogenesis, and metastasis, making it a vital molecule in both the growth and dissemination of colon cancer. Furthermore, IL-8 expression has been significantly associated with poor prognosis in colorectal cancer, especially in stage IV. Therefore, it may serve as a strong prognostic marker in CRC, indicating its potential to enhance prognostic evaluation and inform personalized therapeutic approaches for individual patients.^76–78^

IL-8, iNOS, and COX-2 play a key role in the inflammatory cascade in colon cancer, each contributing to tumor development, survival, angiogenesis, and metastasis. Their synergistic functions in maintaining chronic inflammation make them essential therapeutic targets for reducing tumor progression and enhancing patient outcomes.

Previous studies have confirmed the pro-inflammatory state of undifferentiated Caco-2 cells, as they exhibit significantly higher gene expression of *iNOS* and *PTGS2* (gene that encodes COX-2) compared to their differentiated counterparts.^62^ Consequently, we decided to explore the anti-inflammatory effect of the gold complex **1** on colon cancer cells Caco-2/TC7 measured by *IL-8, iNOS,* and *PTGS2* gene expression after 24 h of incubation (Figure 5), observing that in the experimental conditions, only *IL-8* has been affected without any alteration in *iNOS* or *PTGS2* gene expression.

**Figure 5:**
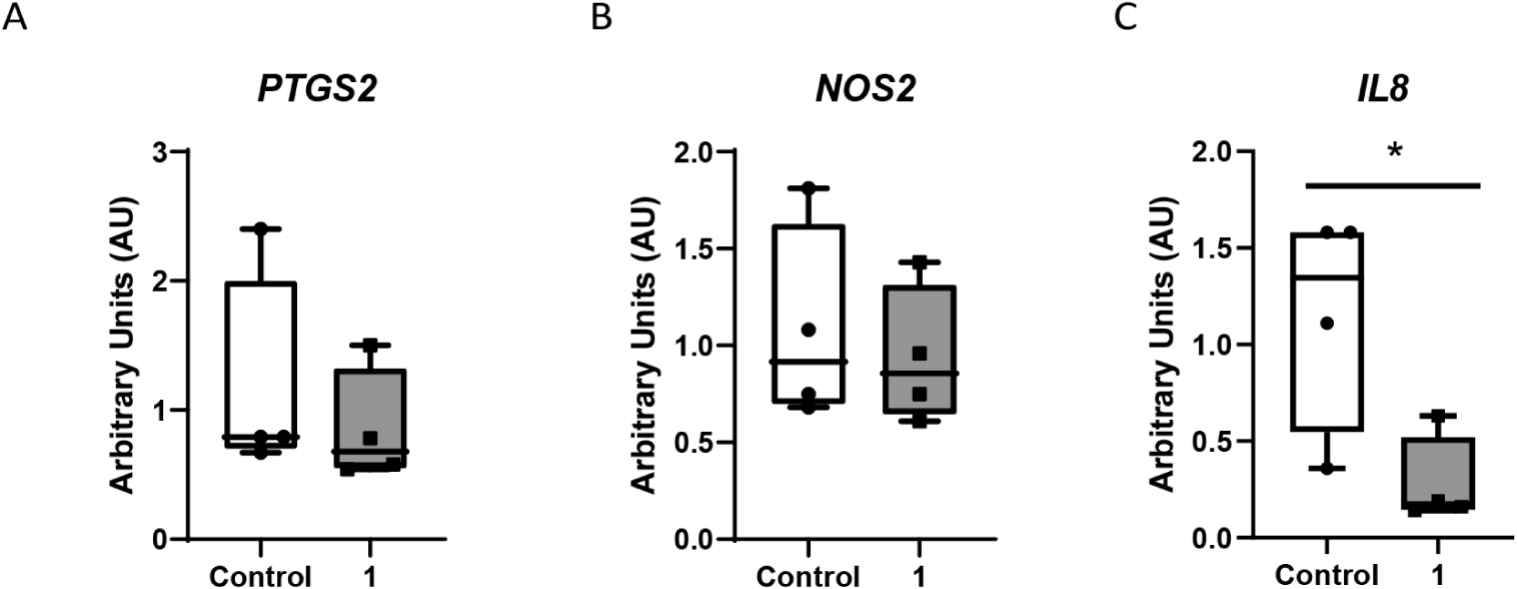
Gene expression of *PTGS2* (A), *NOS2* (B), and *IL-8* (C) measured by qPCR in Caco-2 cells after exposure to complex **1** at its IC_50_ value for 24 h. Data are expressed as boxes (means ± SD) and whiskers (min to max).

This result reflects that complex **1** can inhibit COX-2 activity without affecting *PTGS2* mRNA expression, demonstrating that it regulates this protein at the post-transcriptional level, directly targeting the enzyme’s catalytic function or altering protein dynamics, while leaving gene transcription unchanged. And as a consequence of this regulation mechanism, the inflammatory environment would be reduced as shown by the decrease in *IL8* mRNA expression.

Auranofin has been found to reduce the gene expression of *iNOS*, *TNF*α, *COX-2* and several interleukins in palmitic acid- and LPS (lipopolysaccharide)-stimulated inflammatory macrophages,^79^ as well as suppress proinflammatory cytokines.^80^ Similar results have been previously observed in human macrophages stimulated with LPS (Lipopolysaccharide) after incubation with NSAIDs.^81^

#### 2.7.4 Cell Death Studies

Once the mechanism of action of the multitarget complex [Au­(L1)­JP] (**1**) has been evaluated, we analyzed its potential capacity to induce apoptosis by using a combination of the apoptotic markers 3,8-diamino-5-{3-[diethyl­(methyl)­ammonio]­propyl}-6-phenylphenanthridiniumdiiodide (propidium iodide, PI) and annexin V and compared it to untreated cells. As shown in Figure 6A, no significant changes in the percentage of cells undergoing necrosis were noticed after 48 h or 72 h of incubation with complex **1**. Furthermore, incubation of complex **1** at its IC_50_ value resulted in only 5.25% apoptotic cell death, which increased to 18.07% at 2x IC_50_ and augmented to 30.24 and 37.42% after 72 h incubation at IC_50_ and 2× IC_50_ respectively. Given the slight increase in the late apoptotic cell population, along with a nonsignificant rise in early apoptotic cells, during the first 48 h it cannot be concluded that incubating cells with complex **1** causes cell death by apoptosis alone, as additional forms of cell death might also be involved. However, the results suggest that the complex requires prolonged incubation times to induce significant apoptosis.

**Figure 6:**
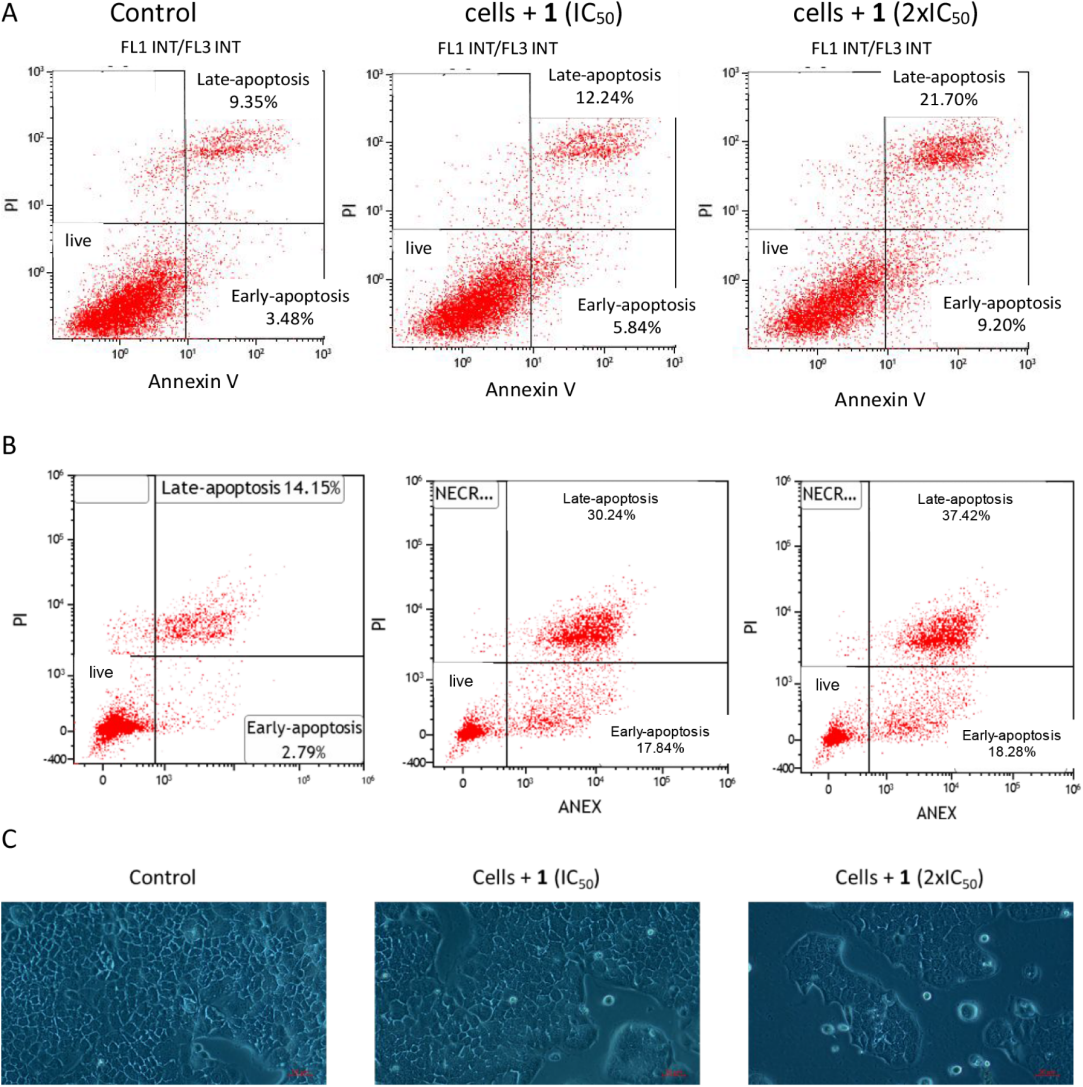
Analysis of the type of cell death induced on undifferentiated Caco-2/TC7 cells after incubation with [Au­(L1)­JP] (**1**). Percentages of alive, necrotic, early apoptotic and late apoptotic cells at its IC_50_ and 2xIC_50_ values after 48h (A) and after 72h (B). C) Live cell imaging by optical microscopy using a 10× objective in Caco-2/TC7 cells with and without complex **1** (1.5 μM) after 48 h incubation; Scale bar: 50 μm.

Besides, live cell imaging was performed and microscopy images of Caco-2/TC7 cells were monitored to observe morphological changes in treated cells with complex **1** at the same concentrations shown in the apoptosis measurement assays (Figure 6B). While control cells showed a continuous extension of the cell layer, nonsignificant changes in cell morphology can be observed in the images after complex **1** incubation, which could suggest that the decrease in cell viability could also be related to a possible cytostatic effect of the gold complex tested.

Mitochondria play essential roles in cellular energy production, metabolic regulation, cell death signaling, and the generation of reactive oxygen species (ROS). The regulation of intracellular ROS levels is associated with cell cycle arrest, induction of apoptosis, autophagy, and decreased viability and invasiveness of cancer cells.^82^ Additionally, alterations in mitochondrial membrane potential (MMP) play a crucial role in regulating cell death. Loss of MMP often results from the opening of the mitochondrial permeability transition pore, which increases the permeability of the inner mitochondrial membrane, leading to the loss of the proton gradient, ATP depletion, and mitochondrial swelling. These events are hallmarks of mitochondrial dysfunction and apoptotic induction.^83^ Gold-based chemotherapeutic agents, such as auranofin, have been shown to induce mitochondrial permeability changes by inhibiting mitochondrial thioredoxin reductase, leading to MMP loss and activation of apoptosis.^84^ To evaluate mitochondrial integrity upon 48 h incubation with complex **1** at a concentration of 1.5 μM, we measured the uptake of the cationic dye DiIC1 (1,1′,3,3,3′-hexamethylindodicarboxy-cyanine iodide). As shown in Figure 7A, treatment with complex **1** increased the percentage of cells with altered MMP compared to untreated cells, suggesting mitochondrial dysfunction. Furthermore, incubation with complex **1** alters cell cycle progression. Notably, there is an accumulation of cells in the G1 phase, which is observed (rising from 52.97 to 62.63%), alongside a decrease in both the S phase and G2/M phase (Figure 7B), similar to the effects observed in auranofin.^85^ This finding suggests that treatment with the gold complex induces a G1 phase arrest, preventing cells from progressing through DNA synthesis and mitosis and thereby reducing cell proliferation.

**Figure 7:**
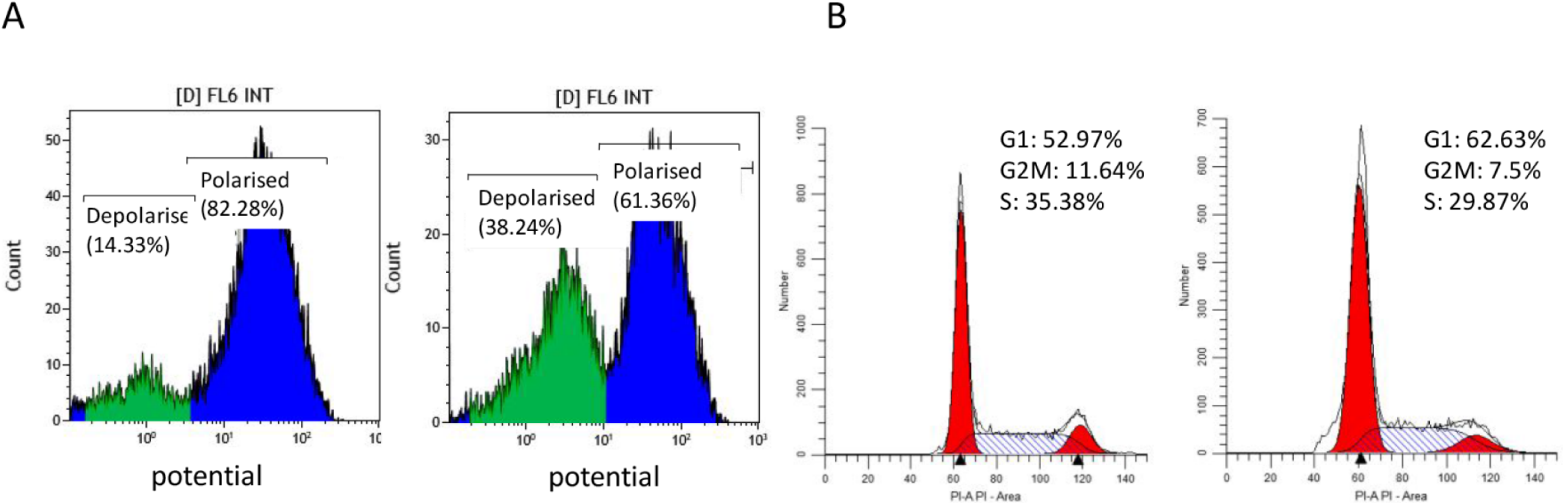
A) Analysis of the integrity of mitochondrial membrane potential in terms of loss of fluorescence after 48 h of incubation of complex **1** at its IC_50_ value. Percentages of each cell population are included. B) Cell cycle analysis. Percentages of each cell population are included.

## 3 Conclusions

We have synthesized and fully characterized a new family of NSAID-derived alkyne ligands and their corresponding phosphine gold­(I) complexes. These alkynyl gold derivatives display selective antiproliferative activity against Caco-2/TC7 colon cancer cells, behaving as thiol-reactive species capable of exchanging their alkynyl ligands with biologically relevant thiols such as N-acetyl-l-cysteine (NAC) and glutathione (GSH). Among them, the naproxen-based complex [Au­(L1)­JP] (**1**) emerges as the most potent and mechanistically versatile candidate. Complex **1** inhibits mitochondrial thioredoxin reductase, leading to disrupted redox homeostasis, increased intracellular ROS levels, and mitochondrial dysfunction. These effects culminate in G1-phase cell-cycle arrest and only mild apoptosis, suggesting that additional nonapoptotic or cytostatic mechanisms may also contribute to its antiproliferative action. Given the central role of mitochondria in both metabolic control and inflammatory signaling, this dual redox and mitochondrial targeting is particularly relevant. In addition, complex **1** exhibits a distinct anti-inflammatory profile, selectively inhibiting COX-2 activity without altering *PTGS2* gene expression, indicating a post-transcriptional regulatory mechanism. This is accompanied by a marked reduction in *IL-8* gene expression, a key pro-inflammatory cytokine associated with tumor progression and poor prognosis in colorectal cancer. The ability to simultaneously modulate redox stress, inhibit COX-2, and attenuate IL-8-driven inflammation underscores the multitarget therapeutic potential of these NSAID–gold hybrids. Overall, these results position NSAID-derived alkynyl gold­(I) complexes, particularly compound **1**, as promising dual-action agents that integrate anticancer and anti-inflammatory properties, offering a compelling strategy for colorectal cancer intervention.

The NSAID-gold hybrids reported in the paper exhibit a unique multi-target pharmacological profile when compared to previously published metal-NSAID complexes. Specifically, complex **1** can simultaneously modulate redox stress, selectively inhibit COX-2 activity, and attenuate IL-8-mediated inflammatory signaling, whereas most of complexes in the literature mainly concentrate on cyclooxygenase and exclusively in most on them in the isoform COX-2. This combination of activities represents a significant advantage, as it allows for the simultaneous modulation of multiple pathways associated with inflammation and cancer progression, besides enhancement of its therapeutic efficacy while lowering side effects associated with non anti-inflammatory drugs.

## 4 Experimental Section

All chemicals and spectroscopic-grade solvents were commercially acquired and utilized without additional purification. Solvent drying and usage followed standard procedures. [AuCl­(tht)], [AuCl­(JohnPhos)], were prepared according to published procedures, and their experimental data agree with that reported elsewhere. All other reagents were commercially available and used without further purification. ^1^H, ^13^C­{^1^H} and ^31^P­{^1^H} were recorded on a Bruker Avance 400 or a Bruker ARX 300 spectrometers. Chemical shifts (δ ppm) were reported relative to the solvent peaks in the ^1^H, ^13^C spectra or external 85% H_3_PO_4_ in ^31^P. IR spectra were recorded in the range 4000–200 cm^–1^ on a PerkinElmer Spectrum 100 spectrophotometer on solid samples using an ATR accessory. A Bruker MicroToF-Q spectrometer was used for high-resolution mass spectra (HRMS-ESI) equipped with an API-ESI source and a QTOF mass analyzer.

### 4.1 General Procedure for the Preparation of the Ligands (**L1**–**4**)

NSAID (naproxen, ibuprofen, mefenamic acid, or indomethacin; 1.0 mmol) was dissolved in acetone (10 mL). K_2_CO_3_ (553 mg, 4.0 mmol) was added, followed by propargyl bromide (80% solution in toluene, 178 μL, 1.6 mmol). The mixture was heated under reflux for 12 h, cooled to room temperature, and filtered through Celite. The filtrate was concentrated under reduced pressure to afford the desired product, which was used without further purification. (See supplementary for experimental data).

### 4.2 General Procedure for the Preparation of the Ligands (**L5**–**6**)

Diflunisal or salicylic acid (1.0 mmol) was dissolved in tetrabutylammonium fluoride (TBAF, 1.0 M solution in THF, 5.0 mL, 5.0 mmol). Propargyl bromide (80% solution in toluene, 178 μL, 1.6 mmol) was added dropwise at room temperature. The reaction mixture was stirred overnight (16 h), then diluted with water (15 mL) and extracted with ethyl acetate (3 × 10 mL). The combined organic layers were dried over MgSO_4_, filtered off, and concentrated under reduced pressure. The crude product was purified by flash column chromatography on silica gel (hexane/EtOAc, 8:2) to afford the desired ligand. (See supplementary for experimental data).

### 4.3 General Procedure for the Preparation of the Ligands (**L7**–**8**)

NSAID (diflunisal or salicylic acid; 1.0 mmol) was dissolved in acetone (10 mL). K_2_CO_3_ (553 mg, 4.0 mmol) was added, followed by propargyl bromide (80% solution in toluene, 356 μL, 3.2 mmol). The mixture was heated under reflux overnight (16 h), cooled to room temperature, and filtered through Celite. The filtrate was concentrated under reduced pressure to afford the desired product, which was used without further purification. (See supplementary for experimental data).

### 4.4 General Procedure for the Preparation of Gold­(I)-Alkynyl Complexes (**1**–**8**)

Ligands **L1–8** (0.10 mmol) in CH_2_Cl_2_ (5 mL) were treated with [Au­(acac)­(JohnPhos)] (0.10 mmol for monoalkynyl ligands, 0.20 mmol for bis-alkynyl ligands). The mixture was stirred at room temperature (r.t.) for 8 h, then concentrated and precipitated with hexane. The solid was collected by filtration, washed with hexane, and dried under vacuum to give the gold­(I) complexes **1–8** (see supplementary for experimental data).

No uncommon hazards are noted

## Supplementary Material



## Notes

Experimental data for the new derivatives and experimental procedures corresponding to stability in buffered solution, BSA interaction, logP_7.4_, interaction with NAC/GSH, cell culture and viability studies, measurement of COX-1/2 activity, analysis of thioredoxin reductase activity, analysis of total cellular oxidative stress, cell death studies, determination of mitochondrial membrane potential, cell cycle analysis, *IL8, iNOS, and PTGS2* gene expression determination, and statistical analysis; in addition, NMR spectra, UV–vis spectra from stability assays and BSA fluorescence quenching spectra (PDF)
