# Supplementary material for: Dual-Action NSAID-Gold(I) Alkynyl Hybrids for Synergistic Anti-Inflammatory and Anticancer Therapy of Colorectal Cancer
Source: Inorg Chem. 2026 Mar 27;65(13):7210–23. doi: 10.1021/acs.inorgchem.5c05908 (PMC13058884; doi:10.1021/acs.inorgchem.5c05908)
Supplement: Supplementary file 1 [file ic5c05908_si_001.pdf]

Supplementary information

Dual-action NSAID–gold(I) alkynyl hybrids for synergistic anti-inflammatory and anticancer therapy of colorectal cancer

Javier Sáez,[a] Luis Vicente Herrera-Marcos,[b] María Jesús Rodríguez-Yoldi,[c] M. Concepción Gimeno,[a] and Elena Cerrada,[a*]*

[a]Departamento de Química Inorgánica. Instituto de Síntesis Química y Catálisis Homogénea (ISQCH). Universidad de Zaragoza-C.S.I.C., 50009 Zaragoza, Spain, E-mail: ecerrada@unizar.es and gimeno@unizar.es

[b]Departamento de Anatomía e Histología Humanas, Facultad de Ciencias de la Salud y del Deporte, Pl. Universidad, 3, 22002 Huesca, Spain

[c]Departamento de Farmacología y Fisiología, Medicina Legal y Forense. Unidad de Fisiología, Facultad de Veterinaria, Ciber de Fisiopatología de la Obesidad y Nutrición (CIBERobn), Instituto Agroalimentario de Aragón (IA2), 50013, Zaragoza, Spain and Instituto de Investigación Sanitaria de Aragón (IIS Aragón), 50009, Zaragoza, Spain.

Table of contents

1 Experimental section
2 Figures **S1-S33**. NMR spectra for ligands and complexes
3 Figures **S34-S44**. Mass spectra of compounds
4 Figures **S46-54**. Stability assays
5 Table **S1**. IC_{50} values of the free ligands
6 Figure **S60-S72**. Interaction studies with NAC and GSH
7 Figure **S73-S85**. BSA studies

Experimental

All chemicals and spectroscopic-grade solvents were commercially acquired and utilized without additional purification. Solvent drying and usage followed standard procedures. [AuCl(tht)], [AuCl(JohnPhos)], were prepared according to published procedures, and their experimental data agree with that reported elsewhere. All other reagents were commercially available and used without further purification. ^{1}H, $^{13}C\{^{1}H\}$ and $^{31}P\{^{1}H\}$

were recorded on a Bruker Avance 400 or a Bruker ARX 300 spectrometers. Chemical shifts (δ ppm) were reported relative to the solvent peaks in the ^{1}H, ^{13}C spectra or external 85% H_3PO_4 in ^{31}P. IR spectra were recorded in the range 4000−200 cm^{-1} on a Perkin-Elmer Spectrum 100 spectrophotometer on solid samples using an ATR accessory. A Bruker MicroToF-Qspectrometer was used for high-resolution mass spectra (HRMS-ESI) equipped with an API-ESI source and a QTOF mass analyzer.

General procedure for the preparation of the ligands (**L1-4**). NSAID (naproxen, ibuprofen, mefenamic acid, or indomethacin; 1.0 mmol) was dissolved in acetone (10 mL). K_2CO_3 (553 mg, 4.0 mmol) was added, followed by propargyl bromide (80% solution in toluene, 178 µL, 1.6 mmol). The mixture was heated under reflux for 12 h, cooled to room temperature, and filtered through Celite. The filtrate was concentrated under reduced pressure to afford the desired product, which was used without further purification

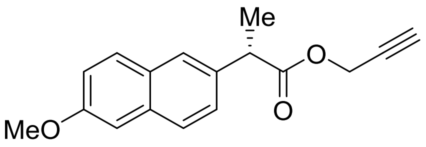

L1: 80 % Yield. White solid. ^{1}H NMR (400 MHz, $CDCl_3$) δ (ppm): 7.71 (m, 3H), 7.71 (m, 3H), 7.41 (dd, J = 8.5, 2.0 Hz, 1H), 7.14 (m, 2H), 4.73 (dd, J = 15.6, 2.5 Hz, 1H), 4.62 (dd, J = 15.6, 2.5 Hz, 1H), 3.91 (s, 3H), 3.89 (q, J = 7.1 Hz, 1H), 2.44 (t, J = 2.4 Hz, 1H), 1.60 (d, J = 7.1 Hz, 3H). ^{13}C NMR (101 MHz, $CDCl_3$) δ (ppm): 174.0, 140.8, 137.3, 129.5, 127.3, 74.9, 52.3, 45.1, 44.9, 30.3, 22.5, 18.6. IR νmax/cm^{-1}: 3248 ν(Csp–H), 2990,2969 ν(Csp^2–H), 2901 ν(Csp^3–H), 2130 ν(Csp≡Csp), 1722 ν(C=O); HRMS (ESI): m/z calcd for $C_{17}H_{16}O_3Na$ $[M+Na]^+$ 291.0992, found: 291.1005; calcd for $C_{17}H_{17}O_3$ $[M+H]^+$ 269.1172, found: 269.1162.

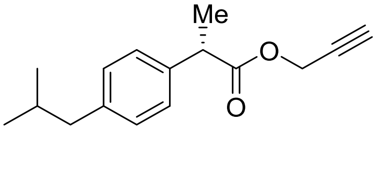

L2: 76 % Yield, white solid. ^{1}H NMR (400 MHz, $CDCl_3$) δ (ppm): 7.16 (m, 4H), 4.72 (dd, J = 15.6, 2.4 Hz, 1H), 4.60 (dd, J = 15.6, 2.5 Hz, 1H), 3.75 (q, J = 7.1, 7.1 Hz, 1H), 2.45 (m, 3H), 1.85 (dp, J = 13.8, 6.7, Hz, 1H), 1.51 (d, J = 7.1 Hz, 3H), 0.90 (d, J = 6.6 Hz, 6H). $^{13}C\{^{1}H\}$ NMR (101 MHz, $CDCl_3$) δ (ppm): 174.0, 140.8, 137.3, 129.5, 127.3, 74.9, 52.3, 45.1, 44.9, 30.3, 22.5, 18.6. IR νmax/cm^{-1}: 3249 ν(Csp–H), 2951 ν(Csp^2–H),

2869 ν(Csp³–H), 2132 ν(Csp≡Csp), 1729 ν(C=O). HRMS (ESI): m/z calcd for $C_{16}H_{20}O_2Na$ [M+Na]$^+$ 267.1356, found: 267.1357; calcd for $C_{15}H_{17}O_2$ [M−CH$_3$]$^+$ 229.1223, found: 229.1381.

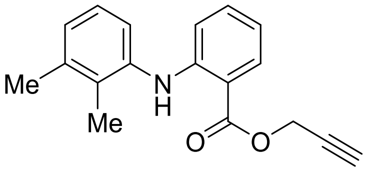

L3: 70% Yield, Pale-yellow solid. ^{1}H NMR (400 MHz, CDCl$_3$) δ (ppm): 9.17 (s, 1H), 8.01 (dd, J = 8.1, 1.8 Hz, 1H), 7.27 (ddd, J = 8.7, 6.9, 1.7 Hz, 1H), 7.10 (m,3H), 6.75 (dd, J = 8.6, 1.3 Hz, 1H), 6.68 (ddd, J = 8.1, 7.0, 1.2 Hz, 1H), 4.94 (d, J = 2.5 Hz, 2H), 2.54 (t, J = 2.4 Hz, 1H), 2.35 (s, 3H), 2.19 (s, 3H). ^{13}C{^{1}H} NMR (101 MHz, CDCl$_3$) δ (ppm): 167.8, 149.9, 138.6, 138.3, 134.7, 132.7, 131.7, 127.1, 126.1, 123.4, 116.2, 113.8, 110.0, 74.9, 52.0, 20.7, 14.1. IR νmax/cm^{-1}: 3356 ν (N-H), 3286 ν(Csp–H), 2987 ν(Csp²–H), 2901 ν(Csp³–H), 2127 ν(Csp≡Csp), 1673 ν(C=O). HRMS (ESI): m/z calcd for $C_{18}H_{17}NO_2Na$ [M+Na]$^+$ 302.1151, found: 302.1163; calcd for $C_{18}H_{18}NO_2$ [M+H]$^+$ 280.1332, found: 280.1334.

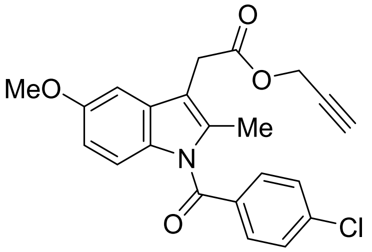

L4. 68 % Yield, Pale-yellow solid. ^{1}H NMR (400 MHz, CDCl$_3$) δ (ppm): 7.55 (m, 4H), 6.96 (d, J = 2.5 Hz, 1H), 6.87 (d, J = 9.0 Hz, 1H), 6.67 (dd, J = 9.0, 2.5 Hz, 1H), 4.71 (d, J = 2.4 Hz, 2H), 3.84 (s, 3H), 3.72 (s, 2H), 2.48 (t, J = 2.4, 2.4 Hz, 1H), 2.39 (s, 3H). ^{13}C{^{1}H} NMR (101 MHz, CDCl$_3$) δ (ppm): 170.1, 168.4, 156.2, 139.4, 136.2, 133.9, 131.3, 130.9, 130.6, 129.2, 115.1, 112.1, 111.9, 101.3, 75.2, 55.8, 52.6, 30.2, 13.5. IR νmax/cm^{-1}: 3285 ν(Csp–H), 2987 ν(Csp²–H), 2901 ν(Csp³–H), 2126 ν(Csp≡Csp), 1676 ν(C=O). HRMS (ESI): m/z calcd for $C_{22}H_{18}ClNO_4Na$ [M+Na]$^+$ 418.0822, found: 418.0835.

General procedure for the preparation of the ligands (**L5-6**).

Diflunisal or salicylic acid (1.0 mmol) was dissolved in tetrabutylammonium fluoride (TBAF, 1.0 M solution in THF, 5.0 mL, 5.0 mmol). Propargyl bromide (80% solution in

toluene, 178 μL, 1.6 mmol) was added dropwise at room temperature. The reaction mixture was stirred overnight (16 h), then diluted with water (15 mL) and extracted with ethyl acetate (3 × 10 mL). The combined organic layers were dried over $MgSO_4$, filtered off, and concentrated under reduced pressure. The crude product was purified by flash column chromatography on silica gel (hexane/EtOAc, 8:2) to afford the desired ligand.

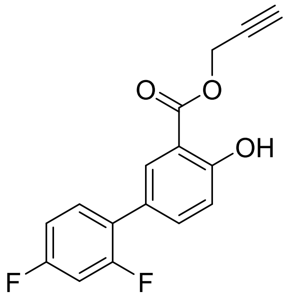

L5. 54% Yield, white solid. ^{1}H NMR (400 MHz, $CDCl_3$) δ (ppm): 10.59 (s, 1H), 8.00 (dd, *J* = 2.4, 1.2 Hz, 1H), 7.62 (dt, *J* = 8.6, 2.0 Hz, 1H), 7.37 (td, *J* = 8.8, 6.4 Hz, 1H), 7.07 (d, *J* = 8.7 Hz, 1H), 6.94 (m, 2H), 4.97 (d, *J* = 2.5 Hz, 2H), 2.56 (t, *J* = 2.5 Hz, 1H). $^{19}F\{^{1}H\}$ NMR (376 MHz, $CDCl_3$) δ (ppm): -111.2 (d, *J* = 7.5 Hz, 1F), -113.7 (d, *J* = 7.4 Hz, 1F). $^{13}C\{^{1}H\}$ NMR (101 MHz, $CDCl_3$) δ (ppm): 169.3, 163.6, 163.5, 161.5, 161.2, 161.1, 161.0, 161.0, 158.6, 158.5, 136.8, 136.8, 131.3, 131.2, 131.2, 131.1, 130.4, 130.3, 126.4, 126.4, 124.2, 124.2, 124.1, 124.0, 118.1, 112.0, 111.9, 111.8, 111.7, 111.6, 104.8, 104.5, 104.5, 104.3, 75.8, 53.0. IR νmax/cm^{-1}: 3295 ν(Csp–H), 3174 ν(O-H), 2972 ν (Csp^2–H), 2901 ν(Csp^3–H), 2126 ν(Csp≡Csp), 1683 ν(C=O). HRMS (ESI): m/z calcd for $C_{16}H_{10}F_2O_3Na$ $[M+Na]^+$ 311.0490, found: 311.0490.

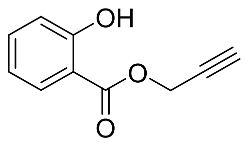

L6. 60% Yield, white solid. ^{1}H NMR (400 MHz, $CDCl_3$) δ (ppm): 10.51 (s, 1H), 7.88 (dd, *J* = 8.0, 1.7 Hz, 1H), 7.48 (ddd, *J* = 8.7, 7.2, 1.8 Hz, 1H), 6.99 (dd, *J* = 8.4, 1.2 Hz, 1H), 6.90 (ddd, *J* = 8.2, 7.2, 1.2 Hz, 1H), 4.95 (d, *J* = 2.5 Hz, 2H), 2.56 (t, *J* = 2.4 Hz, 1H). $^{13}C\{^{1}H\}$ NMR (101 MHz, $CDCl_3$) δ (ppm): 169.1, 161.5, 135.9, 129.9, 119.1, 117.5, 111.6, 75.4, 52.5. IR νmax/cm^{-1}: 3249 ν(Csp–H), 3157 ν(O-H), 2939 ν(Csp^2–H), 2870 ν(Csp^3–H), 2129 ν(Csp≡Csp), 1671 ν(C=O). HRMS (ESI+): *m/z* calcd for $C_{10}H_7O_2^+$ [M-OH]$^+$ 159.0441, found 159.0146; *m/z* calcd for $[C_9H_7O]^+$ 131.0497, found: 131.0038.

General procedure for the preparation of the ligands (**L7-8**).

NSAID (diflunisal or salicylic acid; 1.0 mmol) was dissolved in acetone (10 mL). K_2CO_3 (553 mg, 4.0 mmol) was added, followed by propargyl bromide (80% solution in toluene, 356 µL, 3.2 mmol). The mixture was heated under reflux overnight (16 h), cooled to room temperature, and filtered through Celite. The filtrate was concentrated under reduced pressure to afford the desired product, which was used without further purification.

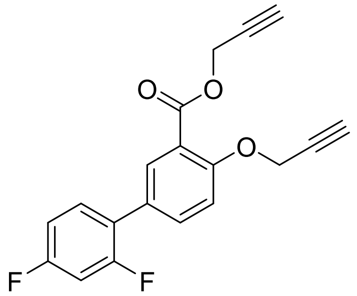

L7. 78% Yield, white Solid. ^{1}H NMR (400 MHz, $CDCl_3$) δ (ppm): 7.99 (dd, *J* = 2.4, 1.2 Hz, 1H), 7.65 (dt, *J* = 8.7, 2.0 Hz, 1H), 7.39 (td, *J* = 8.8, 6.4 Hz, 1H), 7.22 (d, *J* = 8.7 Hz, 1H), 6.94 (m, 2H), 4.92 (d, *J* = 2.4 Hz, 2H), 4.85 (d, *J* = 2.4 Hz, 2H), 2.56 (t, *J* = 2.4 Hz, 1H), 2.50 (t, *J* = 2.4 Hz, 1H). ^{19}F{^{1}H} NMR (376 MHz, $CDCl_3$) δ (ppm): -111.0 (d, *J* = 7.6 Hz, 1F), -113.6 (d, *J* = 7.5 Hz, 1F); ^{13}C{^{1}H} NMR (101 MHz, $CDCl_3$) δ (ppm): 164.5, 163.6, 163.5, 161.1, 161.0, 161.0, 160.8, 158.5, 158.4, 156.9, 134.1, 134.1, 132.3, 132.3, 131.2, 131.2, 131.1, 131.1, 128.2, 128.2, 123.8, 123.7, 123.6, 123.6, 120.2, 114.5, 111.8, 111.7, 111.6, 111.5, 104.7, 104.4, 104.4, 104.2, 77.9, 77.7, 76.4, 75.0, 57.0, 52.4. IR νmax/cm^{-1}: 3296, 3280 ν(Csp–H), 2973 ν(Csp^2–H), 2901 ν(Csp^3–H), 2125 ν(Csp≡Csp), 1683 ν(C=O). HRMS (ESI): m/z calcd for $C_{19}H_{12}F_2O_3Na$ [M+Na]$^+$ 349.0647, found 349.0693; calcd for $C_{19}H_{13}F_2O_3$ [M+H]$^+$ 327.0827, found: 327.0817.

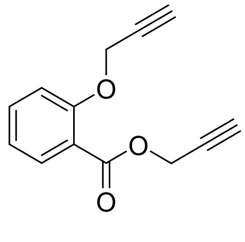

L8. 65% Yield, white solid. ^{1}H NMR (400 MHz, $CDCl_3$) δ (ppm): 7.77 (dd, *J* = 7.8, 1.8 Hz, 1H), 7.39 (ddd, *J* = 8.7, 7.2, 1.8 Hz, 1H), 7.03 (dd, *J* = 8.5, 1.0 Hz, 1H), 6.95 (d, *J* = 1.0 Hz, 1H), 4.79 (d, *J* = 2.6 Hz, 2H), 4.69 (d, *J* = 2.4 Hz, 2H), 2.43 (t, *J* = 2.5 Hz, 1H), 2.40 (t, *J* = 2.5 Hz, 1H). ^{13}C{^{1}H} NMR (101 MHz, $CDCl_3$) δ (ppm): 164.9, 157.5, 133.9, 132.1, 121.4, 120.2, 114.6, 114.6, 76.2, 75.0, 57.0, 52.4. IR νmax/cm^{-1}: 3273, 3239

ν(Csp–H), 2987 ν(Csp^2–H), 2901 ν(Csp^3–H), 2124 ν(Csp≡Csp), 1717 ν(C=O). HRMS (ESI): m/z calcd for $C_{13}H_{10}O_3Na$ $[M+Na]^+$ 237.0522, found: 237.0534.

General procedure for the preparation of gold(I)-alkynil complexes (**1-8**). Ligands **L1-8** (0.10 mmol) in CH_2Cl_2 (5 mL) were treated with [Au(acac)(JohnPhos)] (0.10 mmol for monoalkynyl ligands, 0.20 mmol for bis-alkynyl ligands). The mixture was stirred at room temperature (r.t.) for 8 h, then concentrated and precipitated with hexane. The solid was collected by filtration, washed with hexane, and dried under vacuum to give the gold(I) complexes **1–8.**

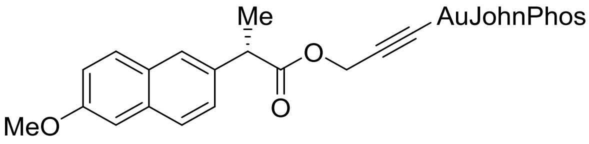

1. 70% Yield, white solid. ^{1}H NMR (400 MHz, DMSO-d_6) δ (ppm): 7.92 (m, 1H), 7.92 (m, 1H), 7.77 (m, 3H), 7.56 (m, 2H), 7.41 (dd, *J* = 8.5, 1.8 Hz, 1H), 7.19 (m, 6H), 7.02 (m, 2H), 4.58 (d, *J* = 15.2 Hz, 1H), 4.52 (d, *J* = 15.2 Hz, 1H), 3.93 (q, *J* = 7.1 Hz, 1H), 3.83 (s, 3H), 1.48 (d, J = 7.1 Hz, 3H), 1.28 (d, J = 14.9 Hz, 18H). ^{31}P{^{1}H} NMR (162 MHz, DMSO) δ (ppm): 64.3. ^{13}C{^{1}H} NMR (101 MHz, DMSO) δ (ppm): 173.4, 157.1, 149.3, 142.1, 135.7, 134.7, 133.7, 133.3, 132.5, 130.5, 129.1, 128.7, 128.6, 128.4, 127.1, 127.0,126.3, 126.3, 125.6, 118.7, 105.7, 93.7, 55.1, 54.0, 44.4, 36.8, 30.3, 18.65. IR νmax/cm^{-1}: 2987,2971 ν(Csp^2–H), 2901 ν(Csp^3–H), 2143 ν(Csp≡Csp), 1720 ν(C=O). HRMS (ESI): m/z calcd for $C_{37}H_{42}AuO_3PNa$ $[M+Na]^+$ 785.2420, found 785.2551; calcd for $C_{37}H_{43}AuO_3P$ $[M+H]^+$ 763.2601, found: 763.2629.

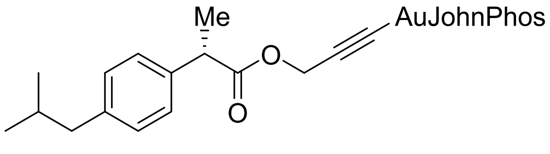

2. 59% Yield, Pale-Yellow Solid. ^{1}H NMR (400 MHz, $CDCl_3$) δ (ppm): 7.77 (td, *J* = 7.1, 7.0, 2.0 Hz, 1H), 7.39 (m, 2H), 7.31 (m, 3H), 7.18 (m, 4H), 7.04 (m, 5H), 4.76 (dd, *J* = 15.3, 1.6 Hz, 1H), 4.61 (dd, *J* = 15.3, 1.6 Hz, 1H), 3.71 (q, *J* = 7.2, Hz, 1H), 2.37 (d, *J* = 7.2 Hz, 2H), 1.76 (dh, *J* = 13.5, 6.7 Hz, 1H), 1.46 (d, *J* = 7.2 Hz, 3H), 1.31 (d, *J* = 15.0 Hz, 18H), 0.82 (d, *J* = 6.6 Hz, 6H). ^{31}P{^{1}H} NMR (162 MHz, $CDCl_3$) δ (ppm): 64.1. ^{13}C{^{1}H} NMR (101 MHz, $CDCl_3$) δ (ppm): 174.5, 150.4, 150.2, 142.3, 142.3, 140.5, 137.9, 134.4, 134.3, 133.1, 133.0, 131.2, 130.3, 130.3, 129.9, 129.3, 129.1, 129.0, 128.3, 127.6, 127.4, 127.3, 126.7, 126.70, 9.1, 94.9, 54.6, 54.5, 45.2, 37.6, 37.4, 31.1, 31.0, 30.2, 22.5, 22.5, 18.9. IR νmax/cm^{-1}: 2952 ν(Csp^2–H), 2865 ν(Csp^3–H), 2143 ν(Csp≡Csp),

1734 ν(C=O). HRMS (ESI): m/z calcd for $C_{36}H_{46}AuO_2PNa$ [M+Na]$^+$ 761.2784, found 761.3022; calcd for $C_{36}H_{47}AuO_2P$ [M+H]$^+$ 739.2965, found: 739.3202.

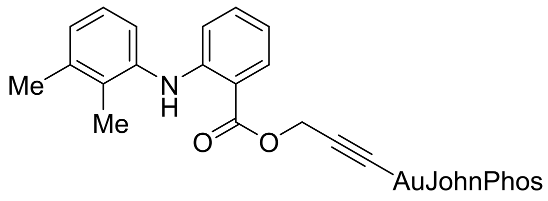

3. 55% Yield. Pale-yellow solid. ^{1}H NMR (400 MHz, $CDCl_3$) δ (ppm): 9.27 (s, 1H), 8.07 (dd, J = 8.0, 1.7 Hz, 1H), 7.77 (td, J = 7.1, 1.6 Hz, 1H), 7.39 (m, 5H), 7.12 (m, 7H), 6.93 (d, J = 7.4 Hz, 1H), 6.69 (d, J = 8.6 Hz, 1H), 6.62 (ddd, J = 8.2, 7.0, 1.1 Hz, 1H), 4.94 (d, J = 1.6 Hz, 2H), 2.25 (s, 3H), 2.13 (s, 3H), 1.32 (d, J = 15.0 Hz, 17H). ^{31}P{^{1}H} NMR (162 MHz, $CDCl_3$) δ (ppm): 64.1. ^{13}C{^{1}H} NMR (101 MHz, $CDCl_3$) δ (ppm): 168.4, 150.4, 150.3, 149.5, 142.4, 142.3, 139.0, 138.2, 134.4, 134.4, 134.1, 133.2, 133.1, 132.6, 132.1, 131.1, 130.3, 130.3, 129.8, 129.2, 129.1, 129.0, 128.2, 127.7, 127.3, 126.7, 126.7, 126.7, 125.9, 123.1, 116.1, 113.6, 111.2, 95.4, 95.2, 54.2, 54.2, 37.6, 37.4, 31.7, 31.1, 31.0, 22.7, 20.7, 14.2, 14.2. IR νmax/cm^{-1}: 2953 ν(Csp^2–H),2897 ν(Csp^3–H), 2143 ν(Csp≡Csp), 1723 ν(C=O). HRMS (ESI): m/z calcd for $C_{38}H_{43}AuNO_2PNa$ [M+Na]$^+$ 796.2580, found: 796.2580.

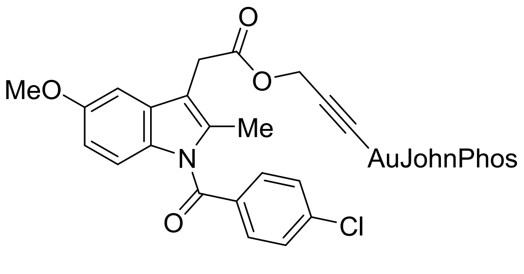

4. 65% Yield. Yellow solid. ^{1}H NMR (400 MHz, $CDCl_3$) δ (ppm): 7.77 (td, J = 7.2, 2.0 Hz, 1H), 7.60 (m, 2H), 7.38 (m, 7H), 7.21 (m, 1H) 7.05 (m, 2H), 6.94 (d, J = 2.5 Hz, 1H), 6.83 (d, J = 9.0 Hz, 1H), 6.60 (dd, J = 9.0, 2.5 Hz, 1H), 4.75 (d, J = 1.6 Hz, 2H), 3.78 (s, 3H), 3.66 (s, 2H), 2.33 (s, 3H), 1.31 (d, J = 15.0 Hz, 18H). ^{31}P{^{1}H} NMR (162 MHz, $CDCl_3$) δ (ppm): 64.1. ^{13}C{^{1}H} NMR (101 MHz, $CDCl_3$) δ (ppm): 170.6, 168.4, 156.2, 150.4, 150.2, 142.4, 142.3, 139.2, 136.0, 134.4, 134.4, 134.1, 133.1, 133.1, 131.6, 131.3, 130.9, 130.9, 130.4, 130.3, 130.3, 129.2, 129.2, 129.0, 128.1, 127.6, 127.2, 126.8, 126.7, 115.0, 112.9, 112.0, 101.3, 94.8, 94.6, 55.9, 54.8, 54.8, 37.6, 37.4, 31.1, 31.0, 30.4, 13.67. IR νmax/cm^{-1}: 2954 ν(Csp^2–H), 2861 ν(Csp^3–H), 2140 ν(Csp≡Csp), 1732 ν(C=O). HRMS (ESI): m/z calcd for $C_{42}H_{45}AuClNO_4PNa$ [M+Na]$^+$ 912.2241, found: 912.2241.

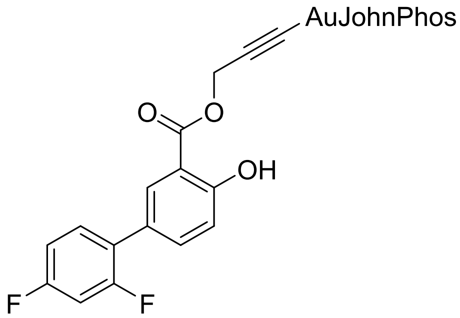

5. 65% Yield. White solid. ^{1}H NMR (400 MHz, $CDCl_3$) δ (ppm): 10.93 (s, 1H), 8.13 (dd, *J* = 2.4, 1.1 Hz, 1H), 7.84 (td, *J* = 7.2, 1.6 Hz, 1H), 7.62 (dt, *J* = 8.6, 2.1 Hz, 1H), 7.45 (m, 6H), 7.26 (m, 1H), 7.14 (m, 2H), 7.07 (d, *J* = 8.7 Hz, 1H), 6.93 (m, 2H), 5.06 (d, *J* = 1.6 Hz, 2H), 1.39 (d, *J* = 15.0 Hz, 18H). ^{31}P{^{1}H} NMR (162 MHz, $CDCl_3$) δ (ppm): 64.1. ^{19}F{^{1}H} NMR (376 MHz, $CDCl_3$) δ (ppm): -111.6 (d, *J* = 7.3 Hz, 1F), -113.5 (d, *J* = 7.3 Hz, 1F). ^{13}C{^{1}H} NMR (101 MHz, $CDCl_3$) δ (ppm): 169.7, 161.3, 150.4, 150.2, 142.5, 142.4, 136.2, 134.4, 133.2, 133.1, 131.7, 131.4, 131.3, 130.7, 130.7, 130.4, 130.4, 129.3, 129.0, 128.1, 127.6, 127.2, 126.8, 126.7, 126.1, 117.8, 112.9, 111.6, 104.5, 94.1, 55.3, 55.3, 37.7, 37.4, 31.1, 31.0. IR νmax/cm^{-1}: 2988, 2960 ν(Csp^{2}–H), 2900 ν(Csp^{3}–H), 2134 ν(Csp≡Csp), 1671 ν(C=O). HRMS (ESI): m/z calcd for $C_{36}H_{36}AuF_2O_3PNa$ $[M+Na]^+$ 805.1943, found: 805.1943.

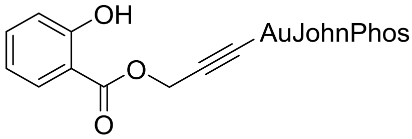

6. 58% Yield. White solid. ^{1}H NMR (400 MHz, DMSO-d_6) δ (ppm): 7.95 (m, 1H), 7.81 (dd, *J* = 8.0, 1.8 Hz, 1H), 7.58 (m, 2H), 7.50 (ddd, *J* = 8.7, 7.1, 1.8 Hz, 1H), 7.31 (m, 3H), 7.20 (m, 1H), 7.08 (m, 2H), 6.97 (dd, *J* = 8.4, 1.1 Hz, 1H), 6.92 (ddd, *J* = 8.1, 7.1, 1.1 Hz, 1H), 4.85 (d, *J* = 1.6 Hz, 2H), 1.31 (d, *J* = 14.9 Hz, 18H). ^{31}P{^{1}H} NMR (162 MHz, DMSO-d_6) δ (ppm): 64.3. ^{13}C{^{1}H} NMR (101 MHz, DMSO-d_6) δ (ppm): 168.3, 161.0, 149.3, 142.2, 135.5, 134.7, 133.7, 132.5, 130.6, 130.6, 130.1, 128.8, 128.6, 127.3, 127.2, 127.1, 126.6, 126.2, 118.6, 117.8, 93.6, 54.6, 36.8, 30.4. IR νmax/cm^{-1}: 2949 ν(Csp^{2}–H), 2861 ν(Csp^{3}–H), 2138 ν(Csp≡Csp), 1667 ν(C=O). HRMS (ESI): m/z calcd for $C_{30}H_{34}AuO_3PNa$ $[M+Na]^+$ 693.1819, found: 693.1819; calcd for $C_{30}H_{35}AuO_3P$ $[M+H]^+$ 671.1997, found: 671.1972.

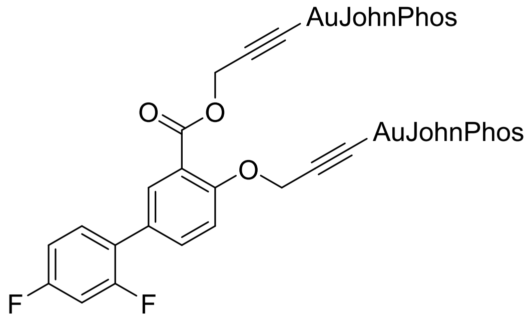

7. 70% Yield. White solid. ^{1}H NMR (400 MHz, DMSO-d$_6$) δ (ppm): 7.93 (m, 2H), 7.83 (m, 2H), 7.64 (td, *J* = 8.9, 6.6 Hz, 1H), 7.56 (m, 4H), 7.41 (d, *J* = 8.8 Hz, 1H), 7.27 (m, 9H), 7.06 (m, 6H), 4.80 (m, 4H), 1.31 (d, *J* = 2.7 Hz, 18H), 1.27 (d, *J* = 2.7 Hz, 18H). ^{31}P{^{1}H} NMR (162 MHz, DMSO-d$_6$) δ (ppm): 64.1, 64.1. ^{19}F{^{1}H} NMR (376 MHz, DMSO-d$_6$) δ (ppm): -111.2 (d, *J* = 7.3 Hz, 1F), -113.9 (d, *J* = 7.6 Hz, 1F). ^{13}C{^{1}H} NMR (101 MHz, DMSO-d$_6$) δ (ppm): 164.7, 160.4, 156.5, 149.4, 149.2, 142.1, 142.0, 142.0, 134.7, 134.7, 133.8, 133.3, 133.2, 132.5, 132.4, 131.7, 131.7, 131.6, 130.5, 130.5, 128.7, 128.6, 128.6, 127.4, 127.2, 127.1, 126.6, 126.5, 126.2, 126.1, 125.9, 123.8, 123.7, 120.6, 114.8, 112.2, 112.0, 104.8, 104.5, 104.3, 94.3, 94.1, 57.6, 54.2, 36.9, 36.7, 30.4, 30.3. IR νmax/cm^{-1}: 2988, 2960 ν(Csp^2–H), 2899 ν(Csp^3–H), 2136 ν(Csp≡Csp), 1726 ν(C=O). HRMS (ESI): m/z calcd for $C_{59}H_{64}Au_2F_2O_3P_2Na$ [M+Na]$^+$ 1337.3497, found: 1337.3497.

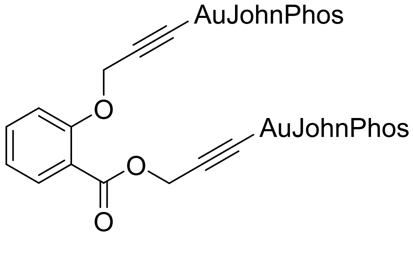

8. 69% Yield. White solid. ^{1}H NMR (400 MHz, DMSO-d$_6$) δ (ppm): 7.95 (m, 2H), 7.95 (m, 2H), 7.72 (dd, *J* = 7.6, 1.8 Hz, 1H), 7.65 (m, 1H), 7.58 (m, 4H), 7.37 (m, 3H), 7.28 (m, 2H), 7.20 (m, 2H), 7.08 (m, 7H), 4.78 (d, *J* = 1.5 Hz, 2H), 4.73 (d, *J* = 1.5 Hz, 2H) ,1.32 (d, *J* = 5.7 Hz, 18H), 1.29 (d, *J* = 5.7 Hz, 18H). ^{31}P{^{1}H} NMR (162 MHz, DMSO-d$_6$) δ (ppm): 64.2, 64.2. ^{13}C{^{1}H} NMR (101 MHz, DMSO-d$_6$) δ (ppm): 165.1, 156.8, 149.3, 142.0, 134.7, 134.0, 132.9,132.7 12.5, 132.4, 130.5, 130.4, 128.7, 128.7, 128.6, 128.6, 127.5, 127.1, 126.6, 126.2,120.5, 119.9, 114.3, 94.4, 94.0, 57.4, 53.9, 36.9, 36.7, 30.4, 30.3. IR νmax/cm^{-1}: 2971 ν(Csp^2–H), 2901 ν(Csp^3–H), 2139 ν(Csp≡Csp), 1724 ν(C=O). HRMS (ESI): m/z calcd for $C_{53}H_{62}Au_2O_3P_2Na$ [M+Na]$^+$ 1225.3390, found: 1225.3390.

Stability in a buffered solution.

The stability of gold(I) complexes **1–8** was studied by UV-visible absorption spectroscopy using a Thermo Scientific Evolution 600 spectrophotometer. Stock solutions of the complexes (10 mM) were prepared in DMSO. Working solutions (50 μM) were prepared by diluting the stock solutions in phosphate-buffered saline (PBS, pH 7.4, 10 mM phosphate, 0.15 M NaCl). The final DMSO concentration was 10% (v/v). UV-visible absorption spectra (200–600 nm) were recorded immediately after preparation (t = 0 h) and at 24, 48, and 72 h during incubation at 37 °C in the dark.

Distribution coefficient (logP).

The n-octanol-water coefficients of the complexes were determined by using a shake-flask method. Buffered-saline distilled water (100 mL, phosphate buffer [PO_4^{3-}] = 10 mM, [NaCl] = 0.15 M, pH 7.4) and n-octanol (100 mL) were shaken for 72 h to allow saturation of both phases. 1 mg of the new complexes was dissolved in 4 mL of the aqueous phase, and 4 mL of the organic phase was added, mixing for 10 minutes. The resulting emulsion was centrifuged to separate the phases. The concentration of the compounds in each phase was determined using UV absorbance spectroscopy. The partition coefficient was calculated as LogP =log{[compound(octanol)]/[compound(aqueous)]}

Interaction with NAC/GSH

The gold complexes 1, 2 and 6 (10 mM) were mixed with an equimolar amount of thiol (N-acetyl-L-cysteine or reduced glutathione, 10 mM) in DMSO-d_6:D_2O (80:20). ^1H NMR and $^{31}P\{^1H\}$ NMR spectra were recorded at room temperature immediately after mixing (t = 0 h) and at 24, 48, and 72 h.

Interaction with bovine serum albumin.

BSA was commercially available from Sigma Aldrich. A 2 mM stock solution of BSA was prepared in PBS at pH 7.4. The real concentration was confirmed by UV-Vis spectroscopy ($\varepsilon_{280\ nm}$ = 43824 $M^{-1}cm^{-1}$). Gold complexes were dissolved in DMSO to obtain 6 mM stock solutions, and aliquots of 2.5 μL were added to a 50 μM solution of BSA in PBS, placed in a quartz cuvette with a 1 cm optical path. The final concentrations of gold complexes in the cuvette were in the range 200 μM. The fluorescence spectra were recorded on a Jobin-Yvon-Horiba fluorolog FL-3-11 spectrometer. The samples were excited at 295 nm, and the emission spectra were recorded from 310 to 450 nm. The samples were measured 240 s after each addition of the gold complex aliquots. The data were analysed using the Stern–Volmer equation $F_0/F = 1 + K_{SV}$ [gold complex] to obtain

the Stern–Volmer quenching constant (K_{SV}). The binding constant (K_b) was quantified by using the Stern–Volmer equation: log $\{(F_0 - F)/F\}$ = log K_b + n log [gold complex].

Phenylbutazone and ibuprofen displacement studies. An equimolar solution of BSA and phenylbutazone or Ibuprofen (50 mM in PBS) was mixed, incubated for 1 h, and then titrated with the gold complex. Titration was monitored fluorometrically at 295 nm.

For UV experiments. UV-visible spectra were recorded over the wavelength range of 200-600 nm. The BSA concentration was fixed at 10 mM, while that of the gold complexes was varied from 10 μM to 50 μM. The absorbance due to the gold complex was nullified by its addition in the reference cell at the same increasing concentration (from 10 μM to 50 μM).

Cell culture.

The human Caco-2 cell line (TC7 clone) was kindly provided by Dr Edith Brot-Laroche (Université Pierre et Marie Curie-Paris 6, UMR S 872, Les Cordeliers, France). The breast adenocarcinoma cell lines MDA-MB-231 and MCF-7 were obtained from the Instituto de Investigación Sanitaria Aragón (IIS Aragón, Spain). Normal human dermal fibroblasts (NHDF-Ad, Lonza®, Porriño, Spain) were kindly provided by Dr Gracia Mendoza (Aragon Health Research Institute, IIS Aragón, Spain).

Caco-2/TC7 cells (passages 36–48), MDA-MB-231 (passages 10–20), MCF-7 (passages 10–20), and NHDF fibroblasts (passages 6-15) were maintained in Dulbecco's Modified Eagle's Medium (DMEM 1×, high glucose 4.5 g/L, GlutaMAX™, without sodium pyruvate; Gibco-Invitrogen, Paisley, UK), supplemented with 10% fetal bovine serum (FBS, lot-tested), 1% non-essential amino acids, and 1% penicillin/streptomycin (100 U/mL and 100 μg/mL, respectively). Cells were incubated at 37 °C in a humidified atmosphere containing 5% CO_2.

For subculture, Caco-2 and MCF-7 cells were seeded in 25 cm^2 plastic flasks at a density of 2×10^5 cells per flask, MDA-MB-231 cells at 1×10^5 cells per flask, and fibroblasts in 75 cm^2 flasks at 3×10^5 cells per flask. Cells were passaged at approximately 80% confluence using 0.05% trypsin-EDTA (Gibco) for detachment. The culture medium was renewed every two days.

Cell viability assay.

All compounds were dissolved in dimethyl sulfoxide (DMSO) to prepare 10 mM stock solutions, which were subsequently diluted in cell culture medium to the desired working concentrations. For cytotoxicity screening, Caco-2/TC7 and MCF-7 cells were seeded at a density of 4×10^3 cells per well in 96-well plates, MDA-MB-231 cells at 6×10^3 cells

per well, and fibroblasts at 6 × 10^3 cells per well. After 24 h, the culture medium was replaced with fresh medium containing the compounds, and the cells were incubated for an additional 48 h. A concentration range of 0.097–100 µM was tested in undifferentiated Caco-2/TC7, MDA-MB-231, MCF-7, and fibroblasts to determine IC_{50} values. The antiproliferative effect was evaluated using the MTT assay. After 72 h of incubation, the culture medium was removed, and the cells were incubated with MTT solution (0.5 mg/mL in PBS) for 3 h at 37 °C. The formazan crystals were dissolved in DMSO (100 µL per well), and absorbance was measured at 550 nm using a FLUOstar Omega microplate reader (BMG Labtech, Ortenberg, Germany). Cell viability was expressed as a percentage relative to untreated control cells. To calculate the selectivity index (SI), the IC_{50} value obtained for fibroblasts was divided by the IC_{50} value for undifferentiated Caco-2/TC7 cells. All experiments were performed in three independent biological replicates. Dose–response curves were fitted by nonlinear regression (log[concentration] *vs*. normalised response) using a 4-parameter logistic (variable slope) model with Top and Bottom constrained to 100% and 0% (relative viability), respectively; IC_{50} and Hill slope were fitted as free parameters. IC_{50} values were computed in GraphPad Prism 8 (GraphPad Software, San Diego, CA). Results are expressed as mean ± SEM.

Intracellular TrxR activity

Caco-2/TC7 cells were seeded at a density of 3.8 × 10^5 cells per well in 6-well plates. After 24 h, the medium was replaced with fresh medium containing compound 1 at its IC_{50} concentration, and cells were incubated for an additional 24 h. Cells were then harvested and lysed with M-PER™ reagent (Thermo Fisher Scientific, 78501) supplemented with an EDTA-free protease inhibitor cocktail. TrxR activity was measured using the Thioredoxin Reductase Assay Kit (Sigma-Aldrich, CS0170) according to the manufacturer's protocol. Reactions were initiated with the DTNB substrate, and TNB formation was monitored at 412 nm every 30 seconds for 5 minutes using a FLUOstar Omega microplate reader (BMG Labtech, Ortenberg, Germany). TrxR activity was expressed as a percentage relative to untreated control cells (assigned as 100% activity). All experiments were performed in triplicate.

Intracellular COX-1/ COX-2 activity

Caco-2/TC7 cells were seeded at a density of 3.8 × 10^5 cells per well in 6-well plates. After 24 h, the medium was replaced with fresh medium containing compound **1** or ligand **L1** at its IC_{50} concentration, and the cells were incubated for an additional 24 h. Cells were then collected and lysed using M-PER™ reagent (Thermo Fisher Scientific, 78501)

supplemented with an EDTA-free protease inhibitor cocktail. COX-1 and COX-2 enzymatic activities were measured separately using the Cyclooxygenase (COX) Activity Assay Kit, fluorometric (Abcam, ab204699), according to the manufacturer's protocol. Reactions were initiated by the addition of arachidonic acid, and fluorescence (Ex/Em = 535/587 nm) was recorded at 5-minute intervals over 90 minutes using a FLUOstar Omega microplate reader (BMG Labtech, Ortenberg, Germany). Enzyme activity was expressed as a percentage relative to untreated control cells. All experiments were performed in triplicate.

Flow cytometry analyses

All flow cytometry experiments were performed using a Beckman Coulter Gallios flow cytometer (Brea, CA, USA). Data were acquired using Kaluza software (Beckman Coulter). All experiments were performed in triplicate.

Intracellular ROS measurement

Caco-2/TC7 cells were seeded in 6-well plates at a density of 2.9×10^5 cells per well. After 24 h, cells were treated with compound **1** at its IC_{50} concentration and incubated for an additional 48 h. Intracellular ROS were measured using the CellROX® Green and CellROX® Orange Flow Cytometry Assay Kits (Molecular Probes, Life Technologies, C10492) according to the manufacturer's instructions. After incubation with the CellROX reagents, cells were processed for flow cytometry, and fluorescence was recorded in the FITC (CellROX Green) and PE (CellROX Orange) channels.

Apoptosis assay

Caco-2/TC7 cells were seeded in 6-well plates at a density of 2.9×10^5 cells per well. After 24 h, cells were treated with compound **1** at $1\times IC_{50}$ or $2\times IC_{50}$ concentrations and incubated for an additional 48 hours. Cells were then transferred to flow cytometry tubes, washed twice with PBS, and resuspended in 100 µL Annexin V binding buffer (10 mM HEPES/NaOH, pH 7.4, 140 mM NaCl, 2.5 mM $CaCl_2$). Annexin V-FITC (5 µL) and propidium iodide (5 µL) were added to each tube. After 15 min of incubation at room temperature in the dark, 400 µL of binding buffer was added, and the samples were analysed within 1 h. Data were acquired in the FITC (Annexin V) and PI channels.

Mitochondrial membrane potential (ΔΨm)

Caco-2/TC7 cells were seeded in 6-well plates at a density of 2.9×10^5 cells per well. After 24 h, cells were treated with compound **1** at $1\times IC_{50}$ or $2\times IC_{50}$ concentrations and incubated for an additional 48 h. ΔΨm was measured using the MitoStep™ Flow Cytometry Mitochondrial Membrane Potential Assay (Immunostep, MITO-100T)

according to the manufacturer's instructions. After staining with the MitoStep reagents, cells were analysed by flow cytometry.

Cell Cycle and DNA content analysis

Caco-2/TC7 cells were seeded in 25 cm^2 flasks at 1×10^6 cells per flask. After 24 h, cells were treated with compound **1** at its IC_{50} concentration and incubated for an additional 48 h. After treatment, cells were fixed in 70% ice-cold ethanol and stored at 4 °C for 24 h. Following centrifugation, cells were rehydrated in PBS and stained with propidium iodide (50 µg/mL) in the presence of RNase A (100 µg/mL). PI-stained cells were analysed for DNA content using a 488 nm laser. Red fluorescence from PI was collected in the 620 nm long-pass channel and displayed on a linear scale. Cell cycle distribution was determined using ModFit LT 3.0 (Verity Software House).

IL8, iNOS and PTGS2 gene expression

To investigate the anti-inflammatory effect of complex **1** in Caco-2 cells, the cells were seeded in a 6-well plate at a density of 5.7×10^4 cells/cm^2. Then, the cells were treated with LDE at 490 µg/mL (IC_{50}) for 24 h. Following the treatment period, the cells were evaluated for the pro-inflammatory genes *NOS2, PTGS2,* and *IL-8* using quantitative reverse transcription PCR (RT-qPCR). RNA was extracted with the *Quick*-RNA Miniprep Kit (ZYMOresearch, Frieburg, Germany). The quality, purity and concentration of the RNA were confirmed by agarose gel electrophoresis and an LVIS plate in the SPECTROstar Nano plate reader. Subsequently, 500 ng of total RNA were reverse-transcribed into complementary DNA (cDNA) using the PrimeScript RT Master Mix kit (TaKaRa Biotechnology, Kusatsu, Shiga, Japan). cDNA synthesis was performed using the First Strand synthesis kit (Thermo Scientific, Madrid, Spain). Changes in mRNA expression were measured by quantitative PCR (qPCR). SYBR Green PCR Master Mix (Applied Biosystems, Foster City, CA) was used to analyse gene expression by qPCR. Specific primers, designed and checked as previously described [1], were purchased from Applied Biosystems. Sequences are shown in Supplemental Table S2. The analysis of the data was conducted using the comparative ΔCt method, with results expressed as $2^{-\Delta\Delta Ct}$ after normalising gene expression to the endogenous control GAPDH. The results were expressed as a relative change in gene expression between the control and treated samples.

Statistical analyses.

All results are expressed as means ± SEM from at least three independent experiments. Statistical comparisons were performed using Student's t-test or one-way ANOVA

followed by Bonferroni post-test. P-values < 0.05 were considered statistically significant. Statistical analyses were carried out using GraphPad Prism 9.0 (GraphPad Software, San Diego, CA, USA).

X-ray diffraction studies

Crystals were mounted on a MiTeGen Crystal micromount and transferred to the cold gas stream of a Bruker D8 VENTURE diffractometer. Data were collected using monochromated MoKα radiation (λ = 0.71073 Å). Scan type ϖ. Absorption corrections based on multiple scans were applied using SADABS.[2] The structures were solved by direct methods and refined on F^2 using the program SHELXT-2018.[3] All non-hydrogen atoms were refined anisotropically.

CCDC 2506181 (complex **3**) contains the supplementary crystallographic data for this paper. These data can be obtained free of charge via www.ccdc.cam.ac.uk/data_request/cif, or by emailing data_request@ccdc.cam.ac.uk, or by contacting The Cambridge Crystallographic Data Centre, 12 Union Road, Cambridge CB2 1EZ, UK; fax: +44 1223 336033.

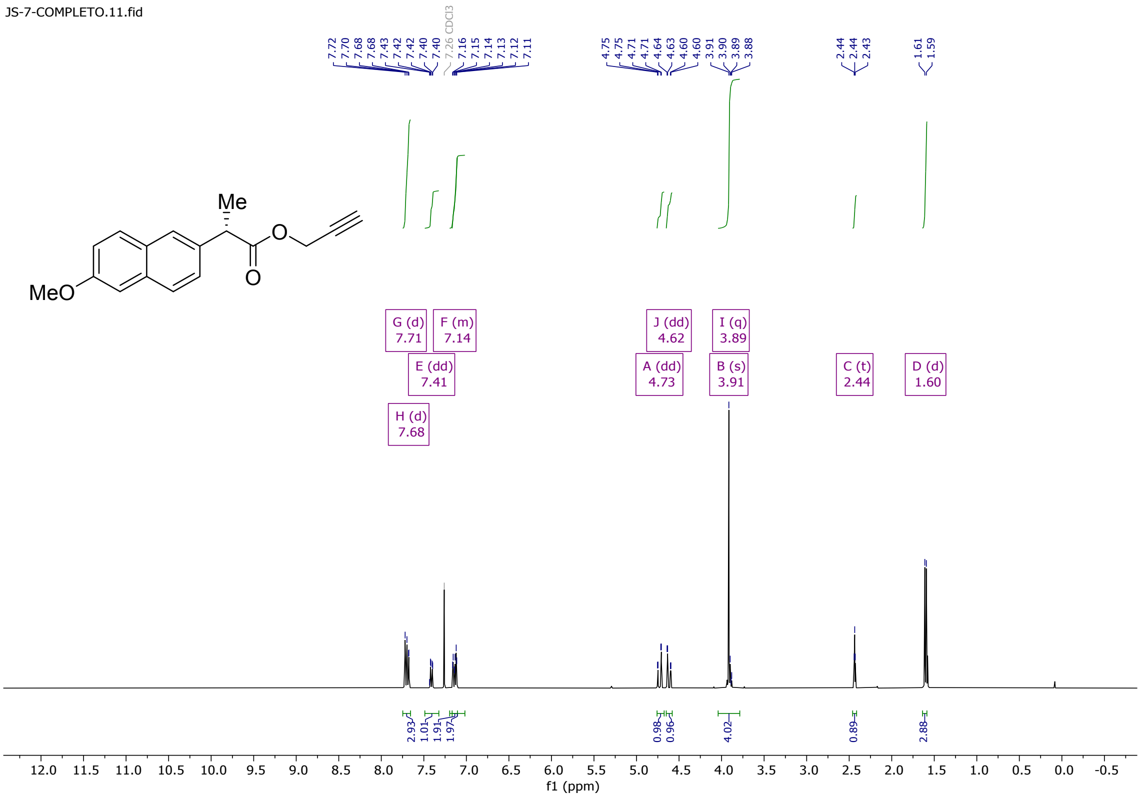

Figure S1. ^{1}H NMR Spectrum (400 MHz, $CDCl_3$) of **L1**.

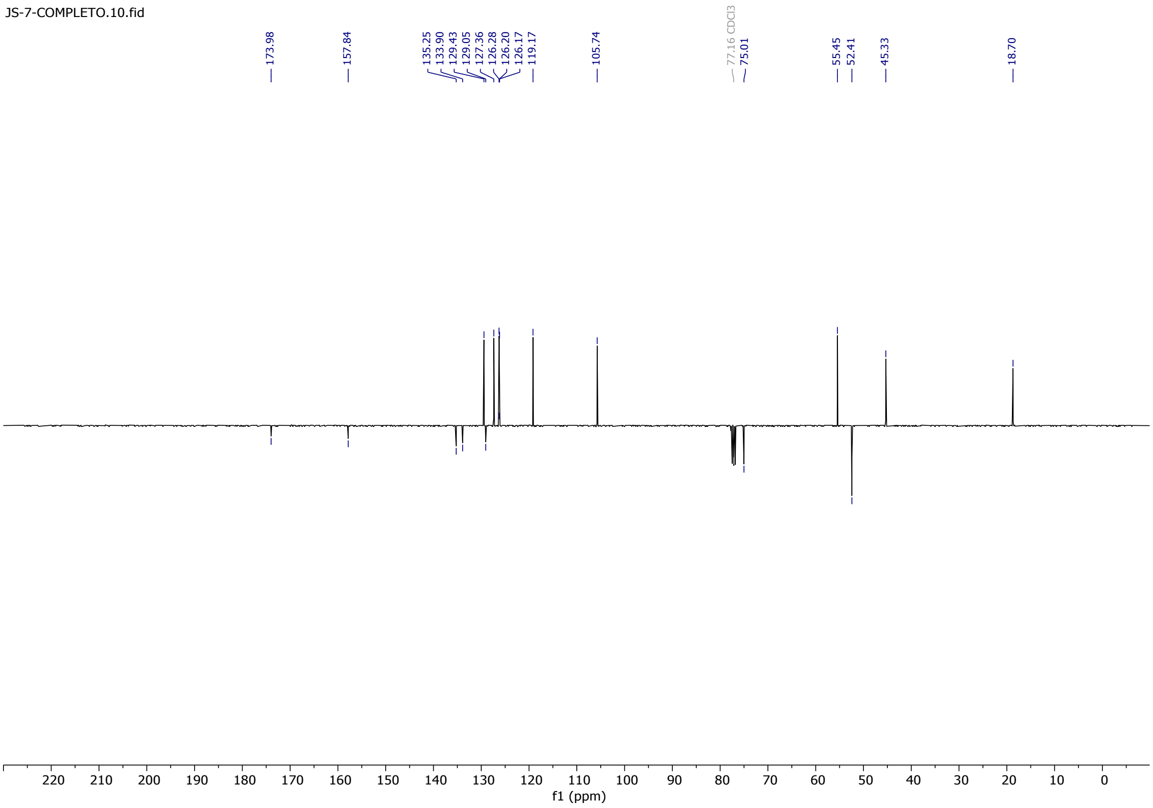

Figure S2.^{13}C {^{1}H} NMR (101 MHz, $CDCl_3$) spectrum of compound **L1.**

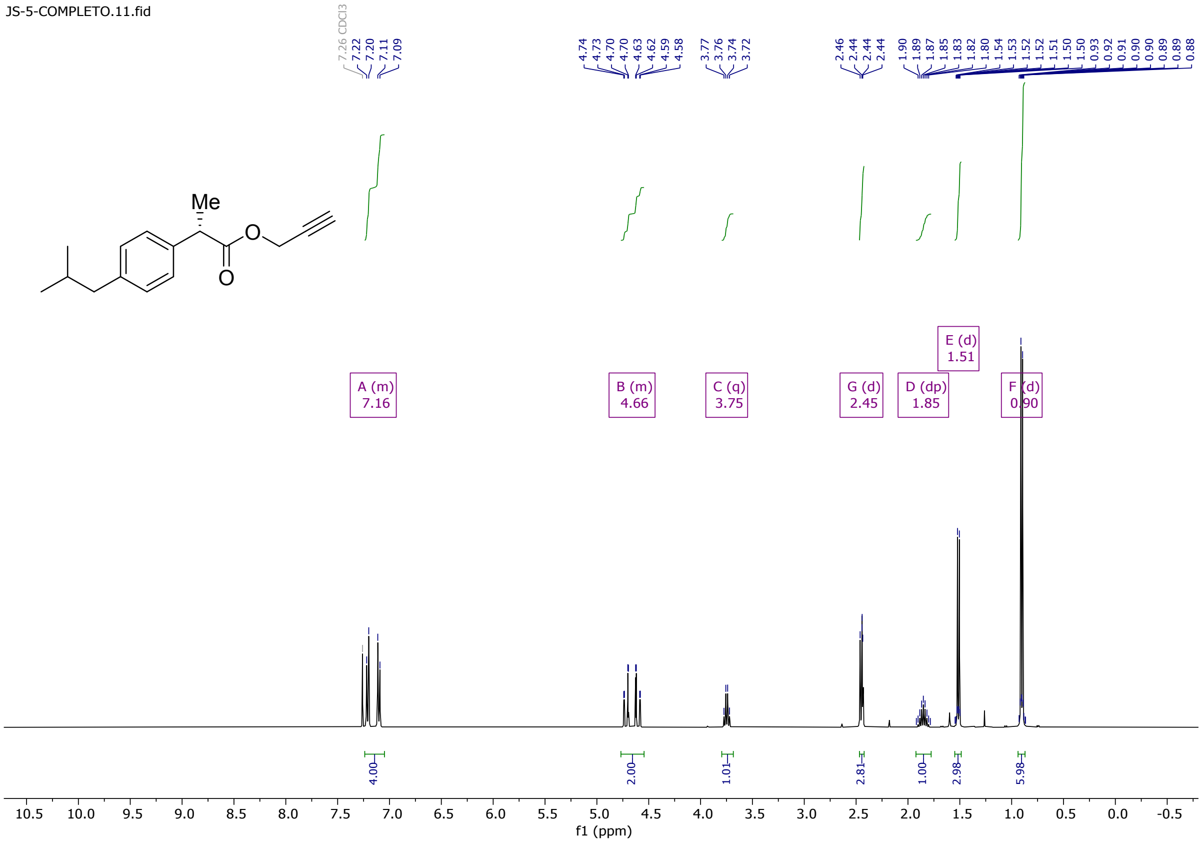

Figure S3. ^{1}H NMR Spectrum (400 MHz, $CDCl_3$) of **L2**.

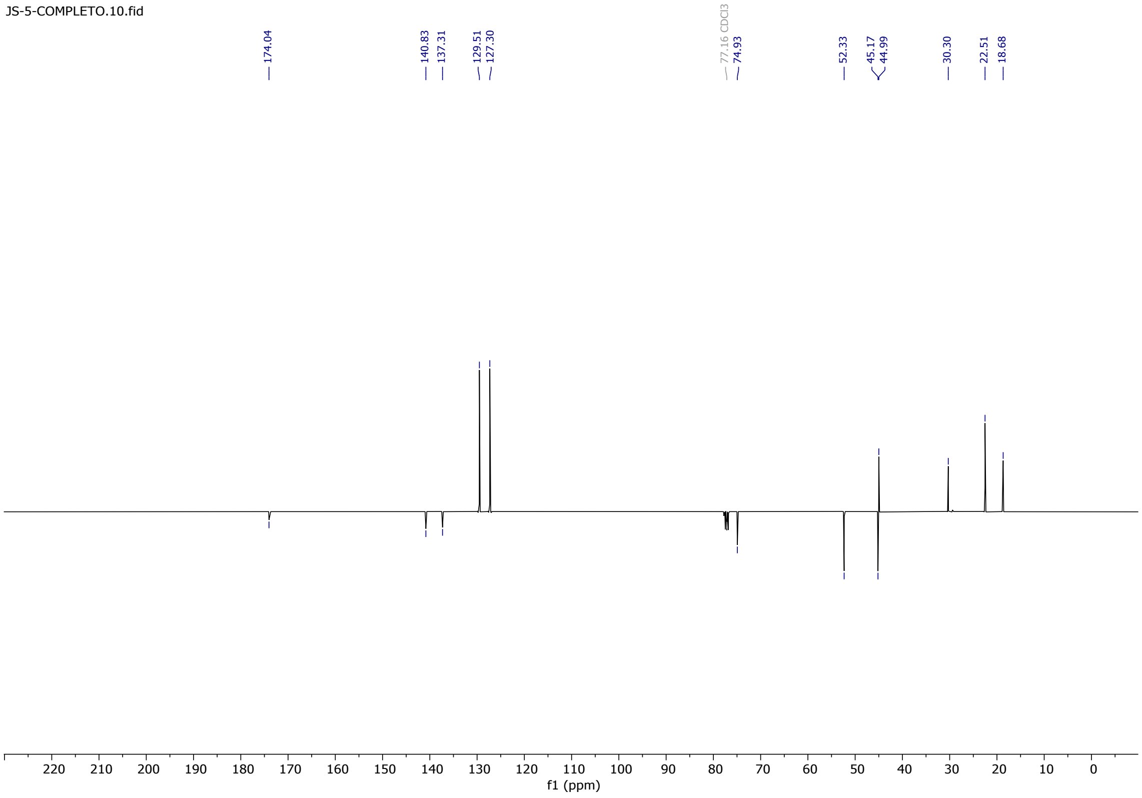

Figure S4. $^{13}C\{^{1}H\}$ NMR (101 MHz, $CDCl_3$) spectrum of **L2.**

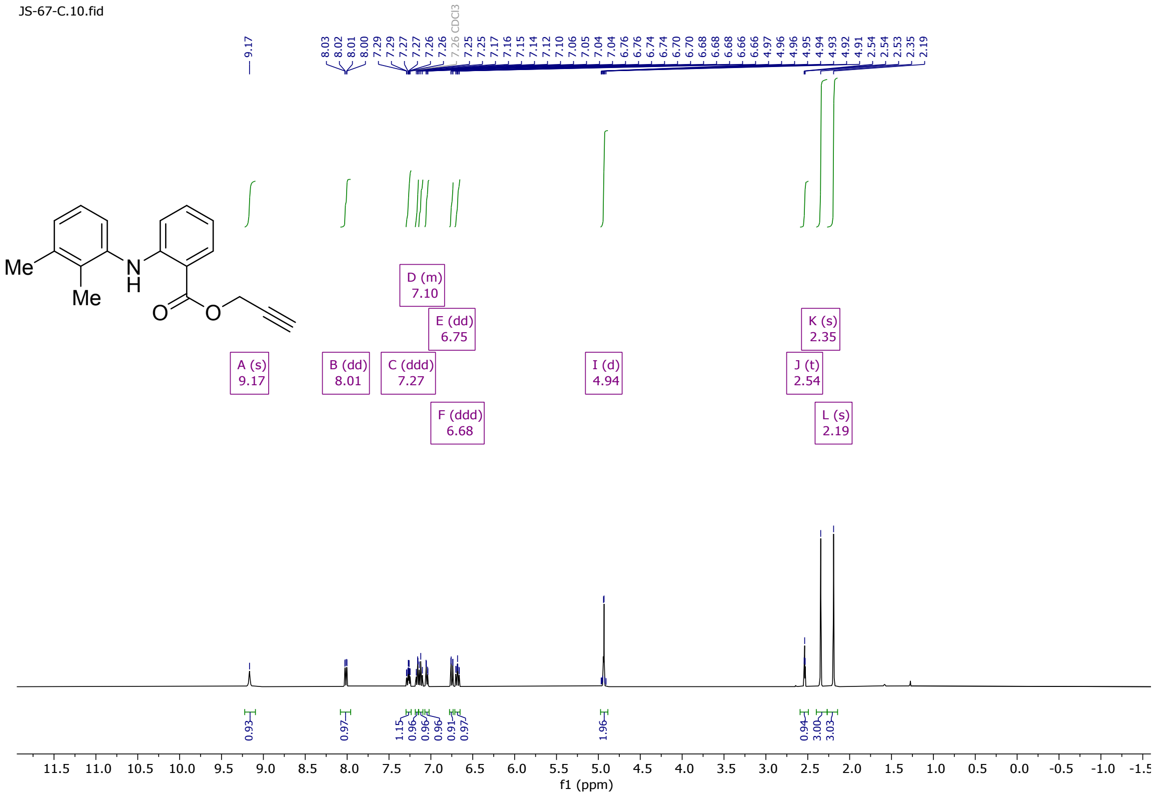

Figure S5. ^{1}H NMR Spectrum (400 MHz, $CDCl_3$) of **L3.**

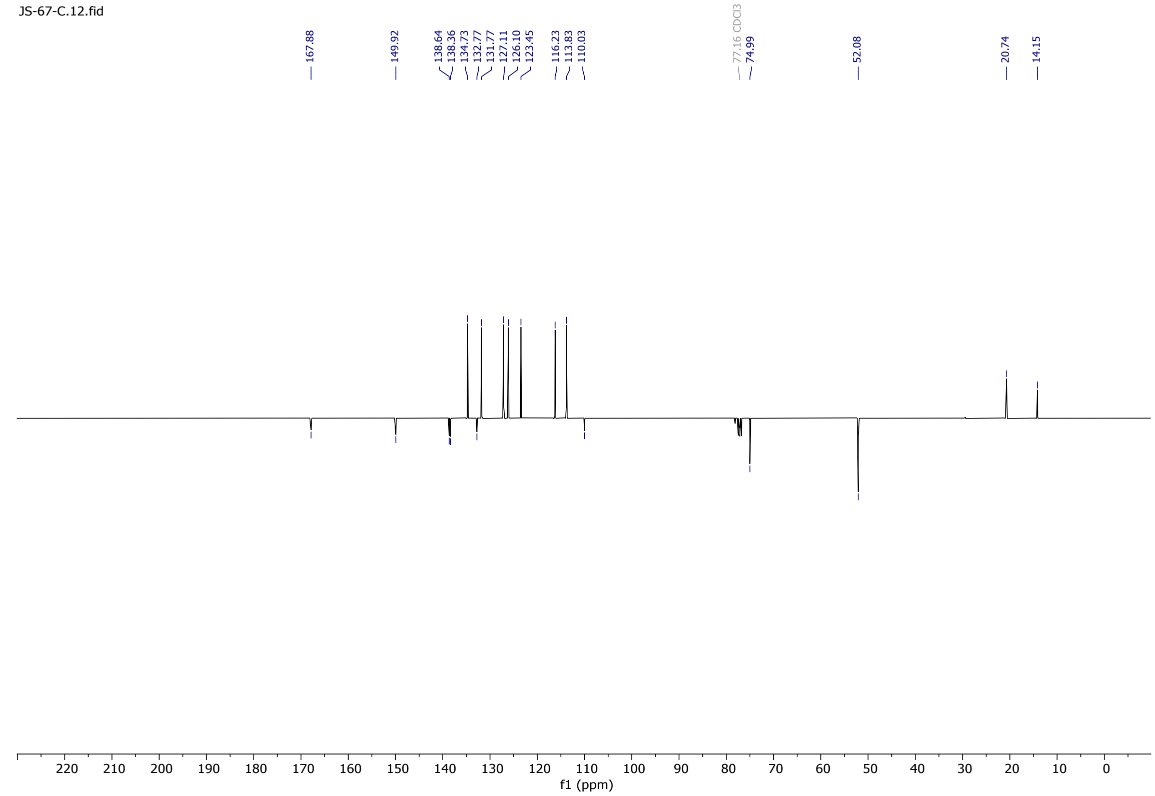

Figure S6. $^{13}C\{^{1}H\}$ NMR (101 MHz, $CDCl_3$) spectrum of **L3**

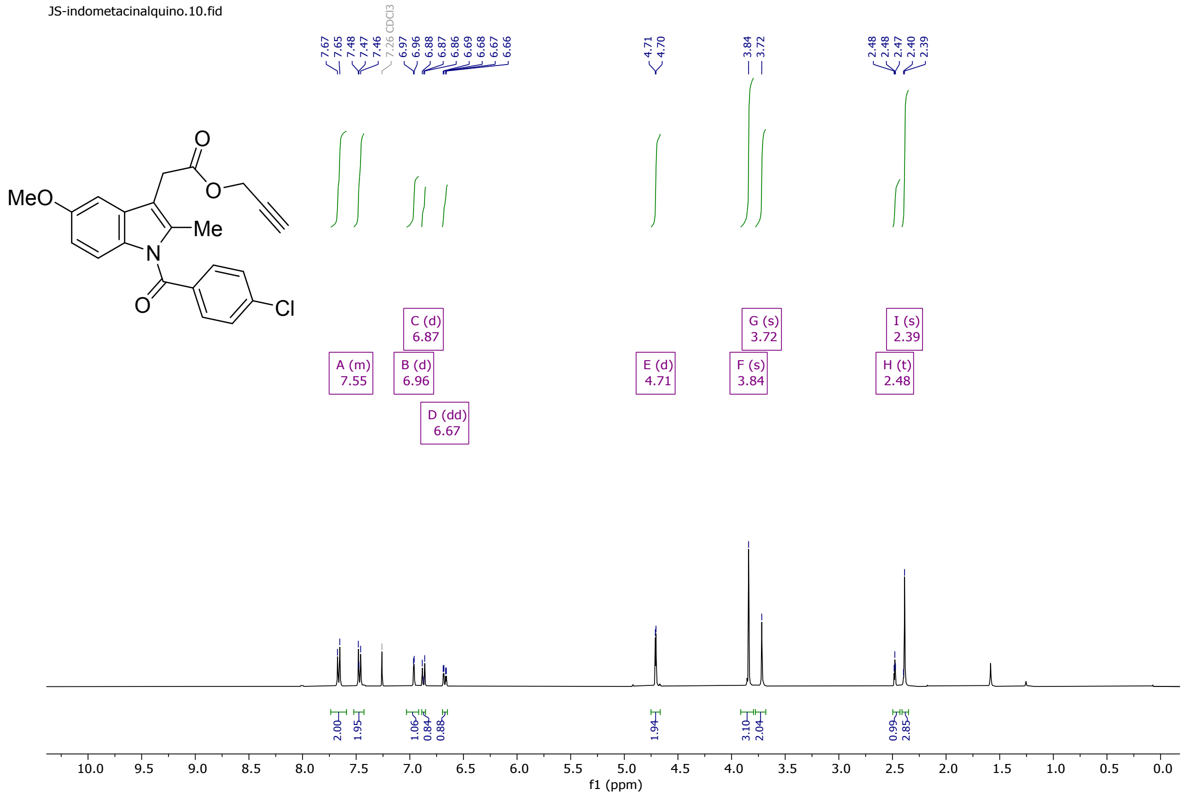

Figure S7. ^{1}H NMR Spectrum (400 MHz, $CDCl_3$) of **L4.**

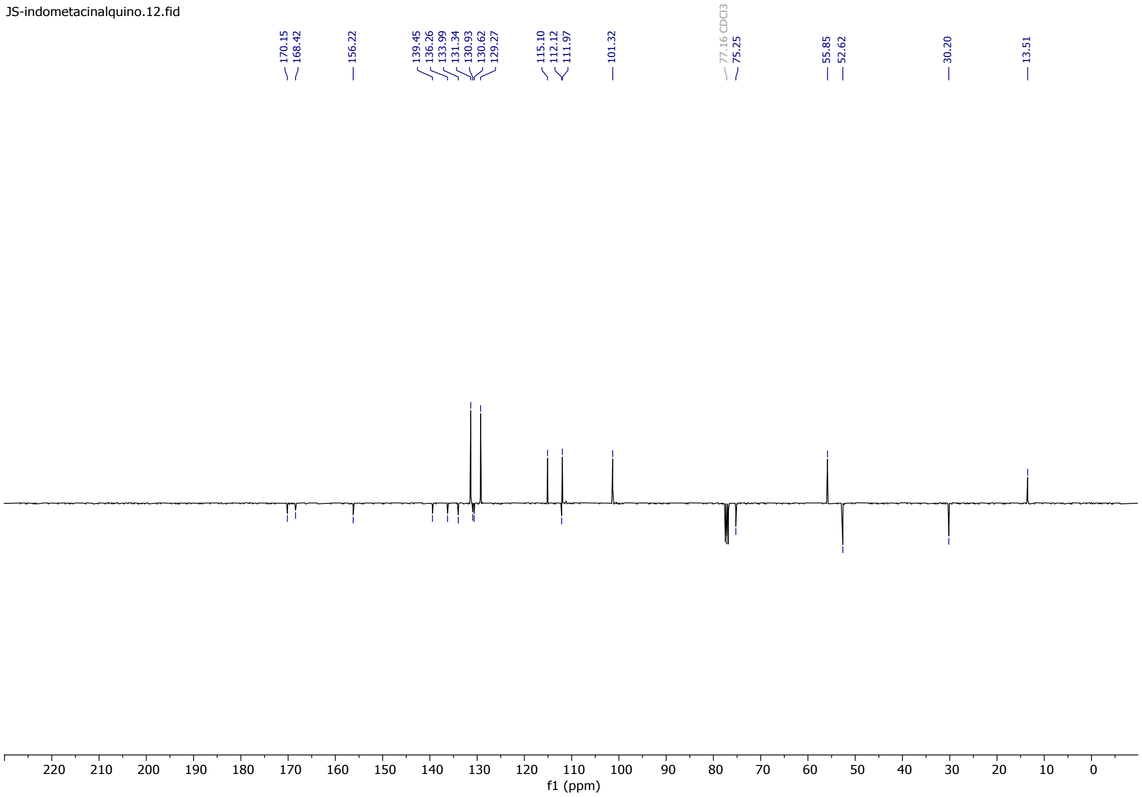

Figure S8. $^{13}C\{^1H\}$ NMR (101 MHz, $CDCl_3$) spectrum of **L4.**

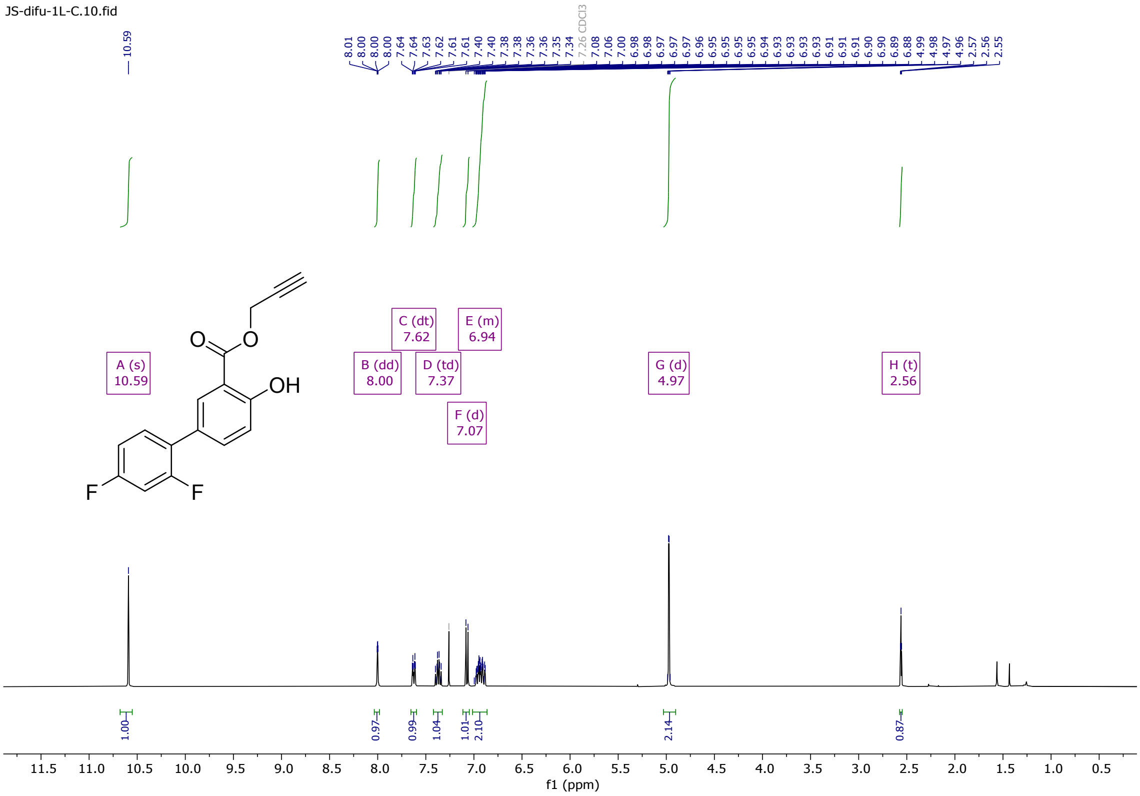

Figure S9.^{1}H NMR Spectrum (400 MHz, $CDCl_3$) of **L5**

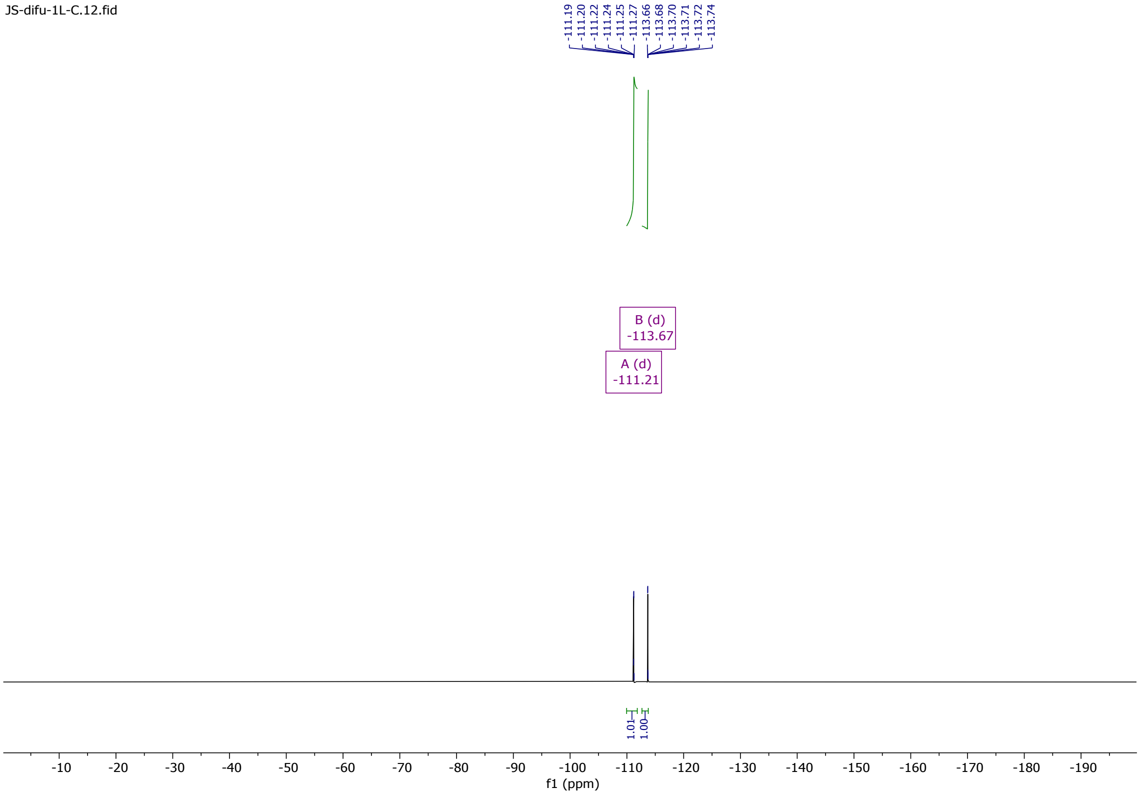

Figure S10. $^{19}F\{^{1}H\}$ NMR spectrum (376 MHz, $CDCl_3$) of **L5.**

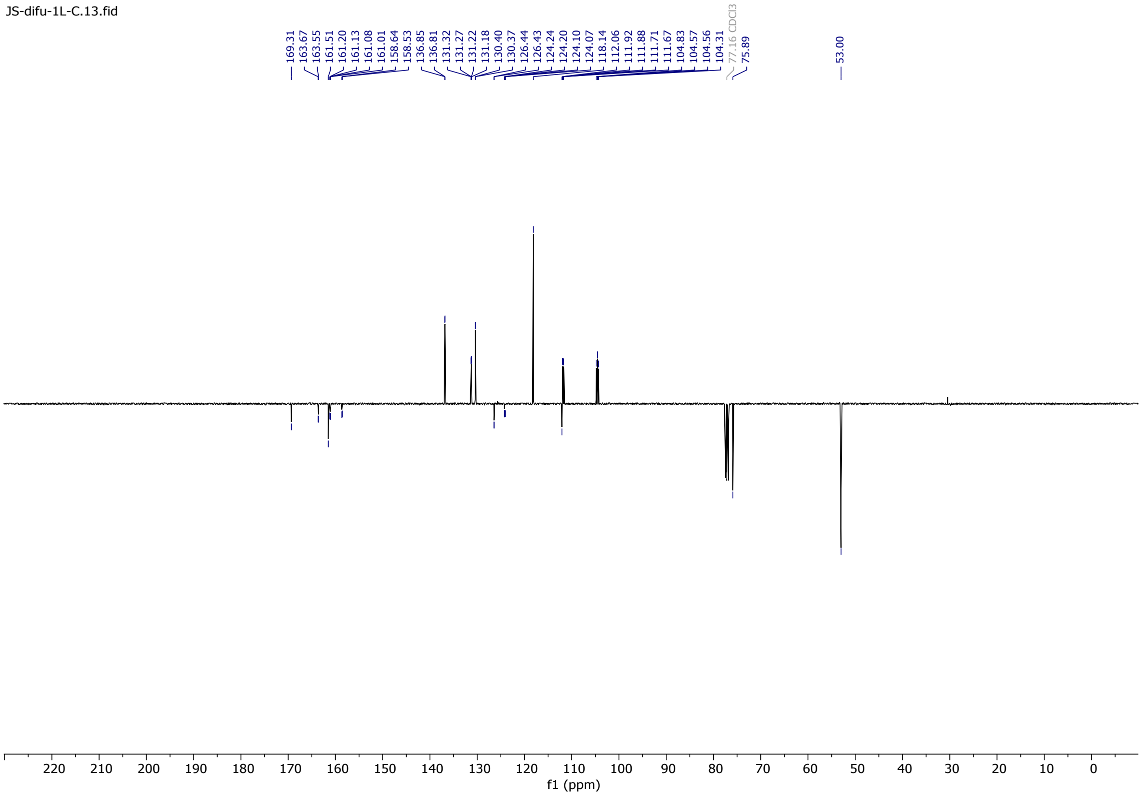

Figure S 11.$^{13}C\{^1H\}$ NMR (101 MHz, $CDCl_3$) spectrum of **L5.**

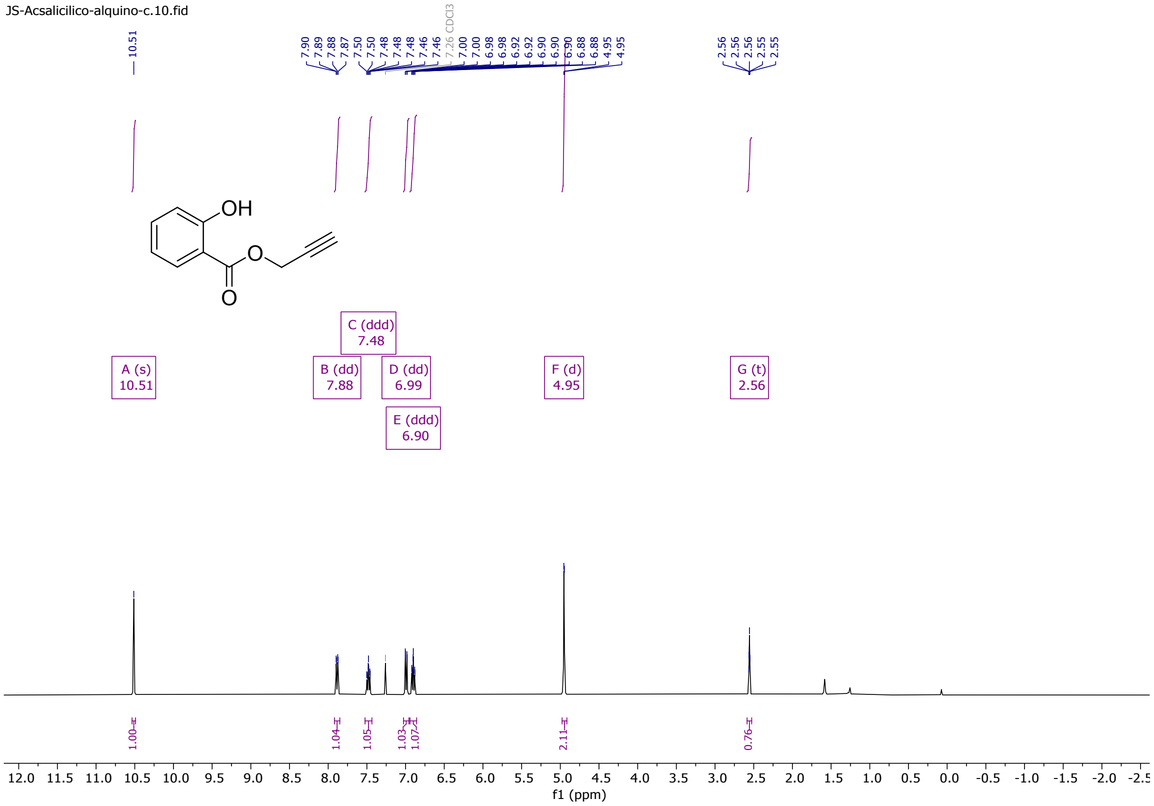

Figure S 12.^1H NMR spectrum (400 MHz, $CDCl_3$) of **L6.**

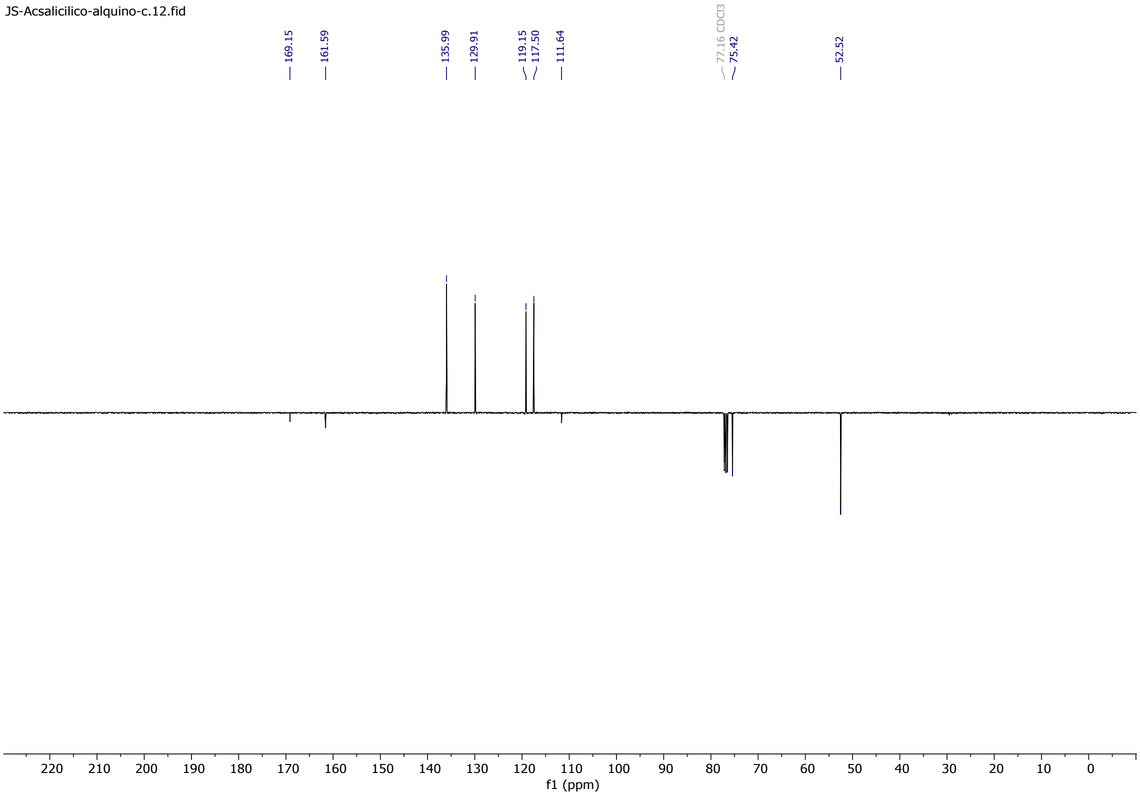

Figure S13. $^{13}C\{^{1}H\}$ NMR (101 MHz, $CDCl_3$) spectrum of **L6.**

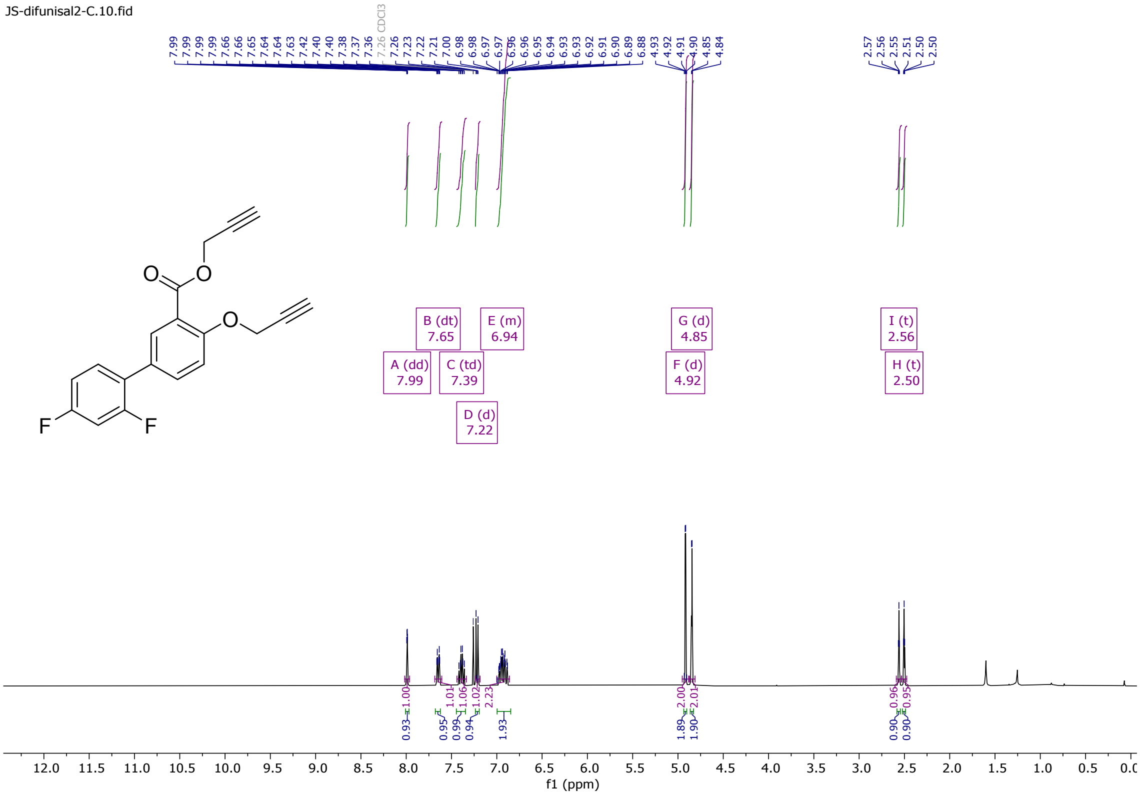

Figure S 14. ^{1}H NMR spectrum (400 MHz, $CDCl_3$) of **L7.**

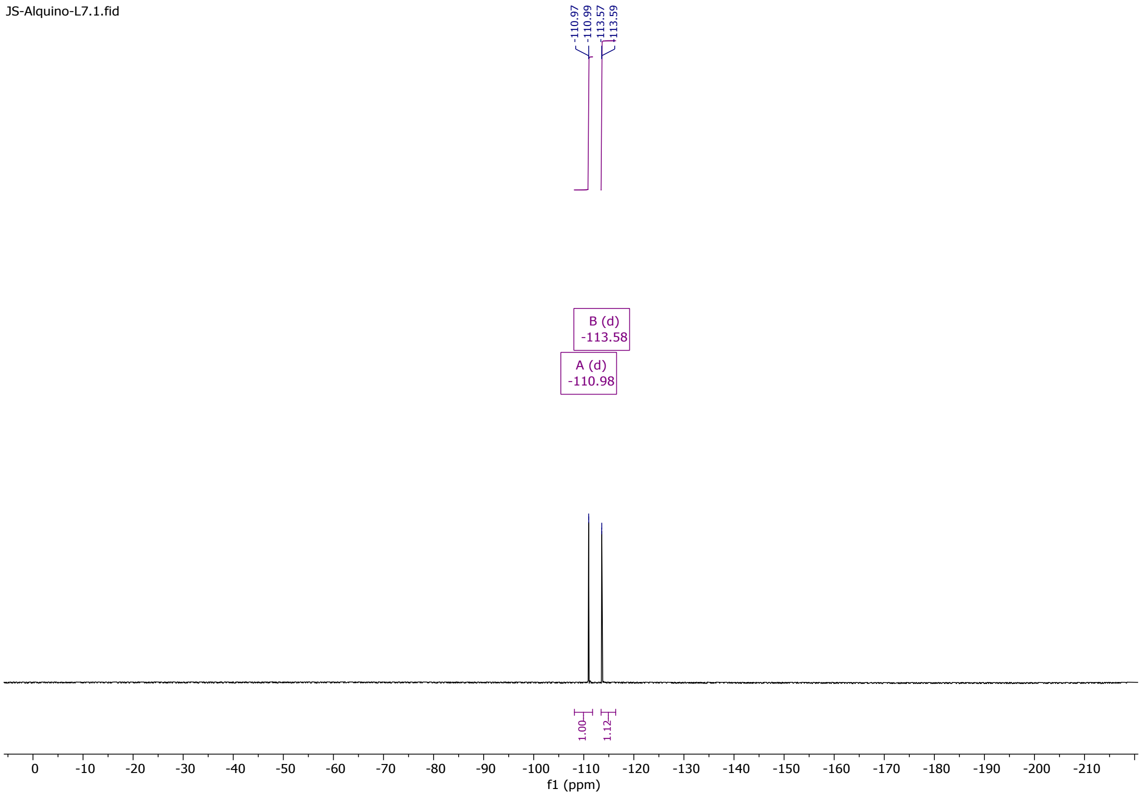

Figure S15. $^{19}F\{^1H\}$ NMR spectrum (376 MHz, $CDCl_3$) of **L7.**

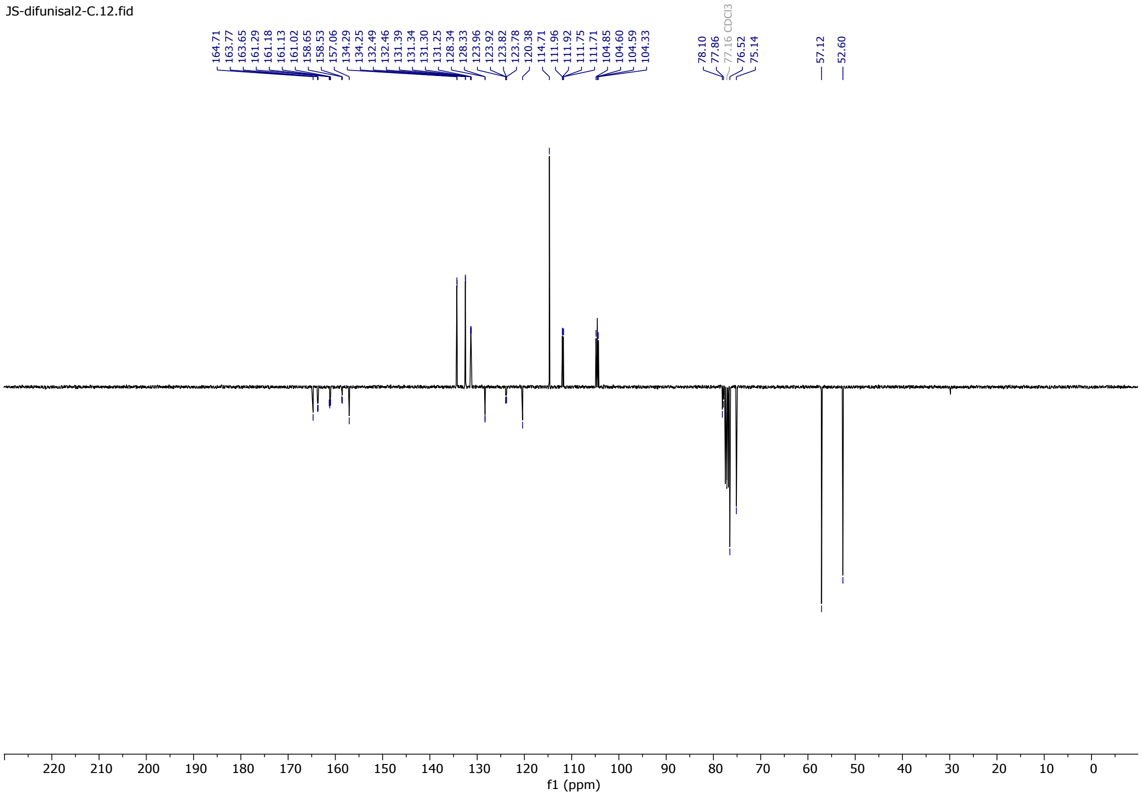

Figure S 16. $^{13}C\{^1H\}$ NMR (101 MHz, $CDCl_3$) spectrum of **L7**

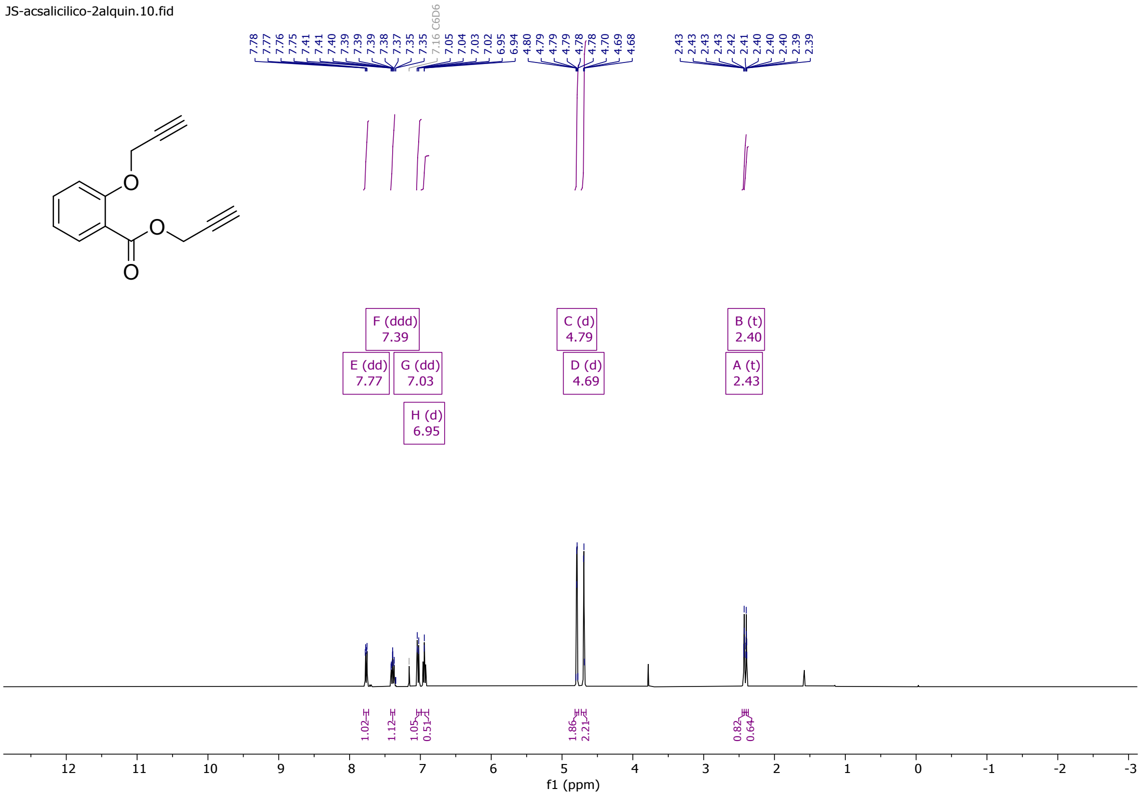

Figure S17.^{1}H NMR spectrum (400 MHz, $CDCl_3$) of **L8.**

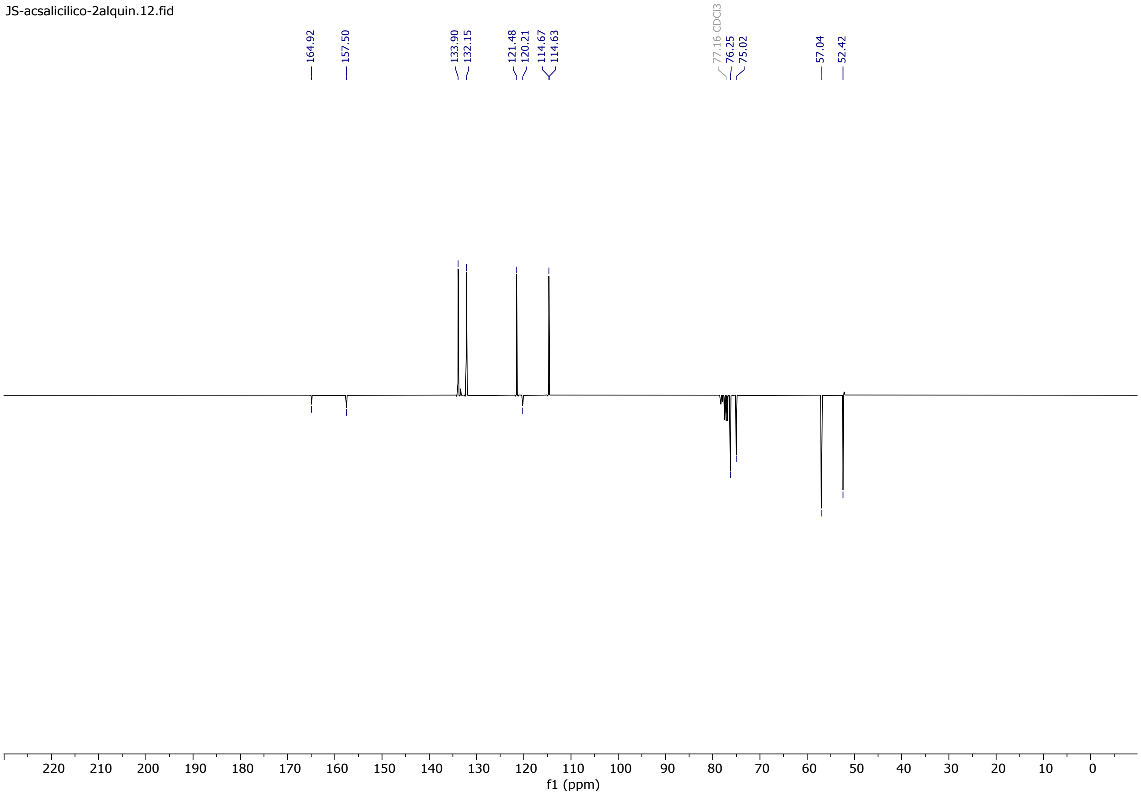

Figure S 18. $^{13}C\{^{1}H\}$ NMR (101 MHz, $CDCl_3$) spectrum of **L8.**

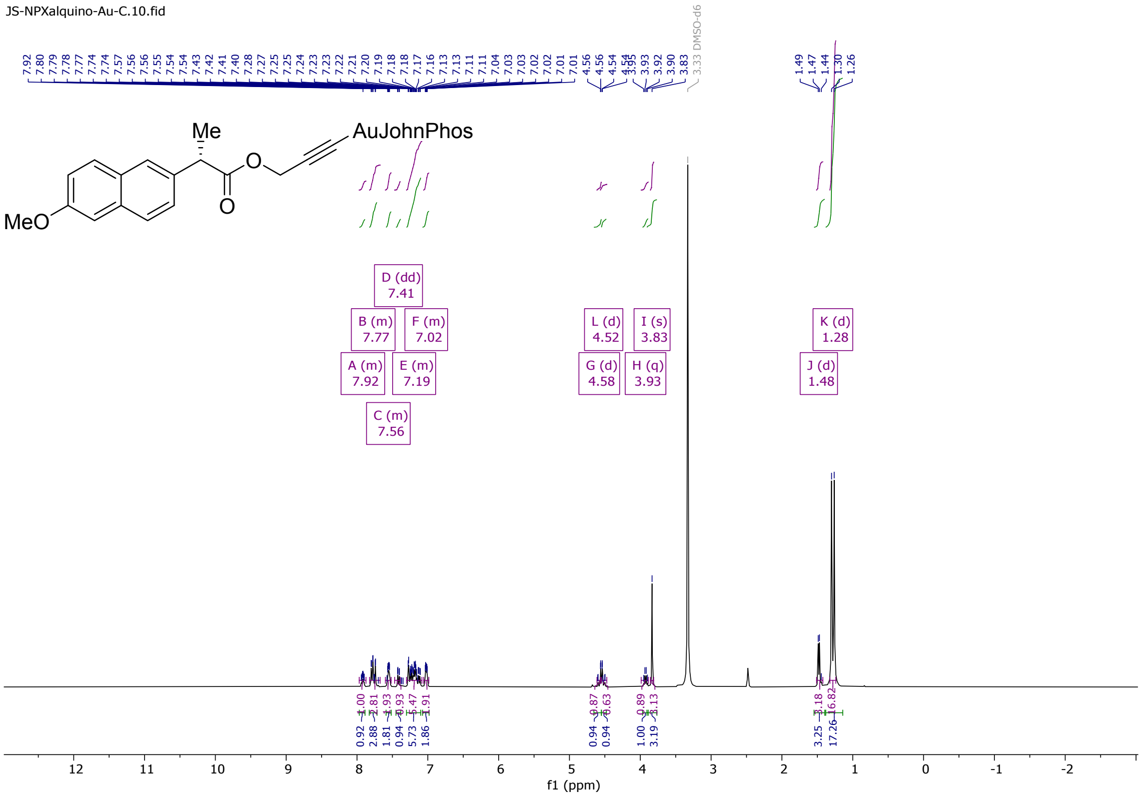

Figure S19.^{1}H NMR spectrum (400 MHz, $CDCl_3$) of **1.**

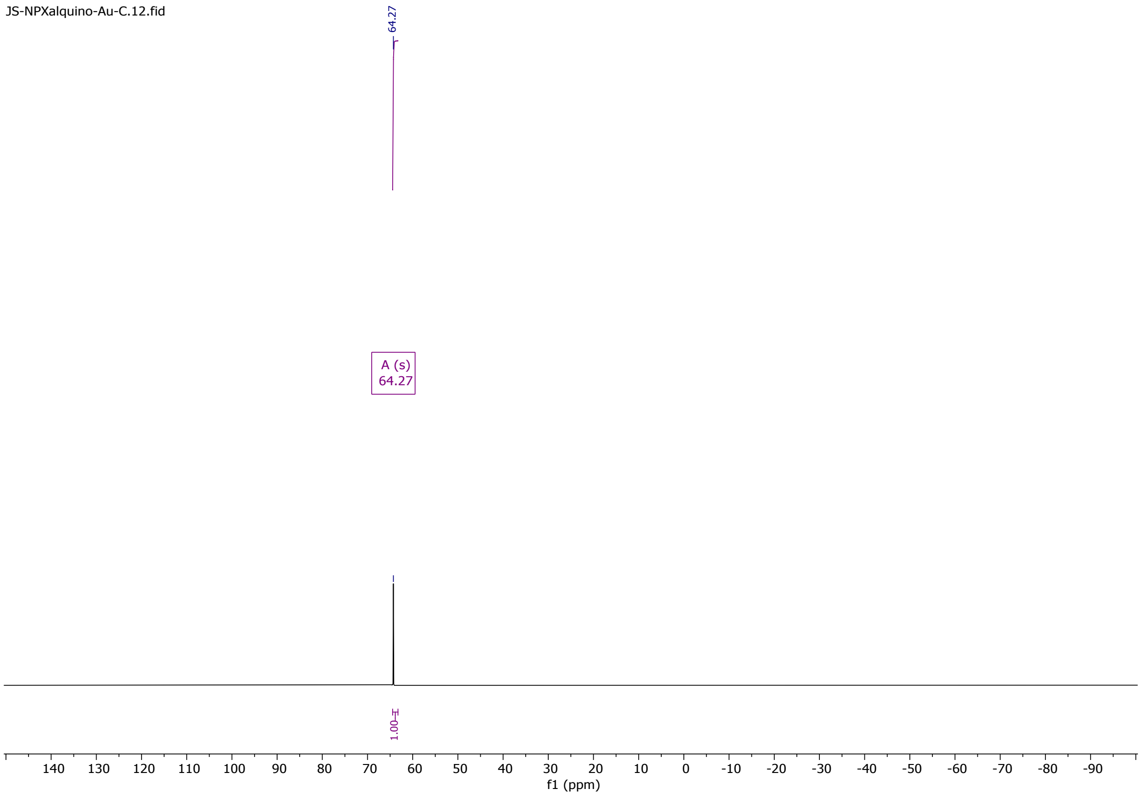

Figure S20.$^{31}P\{^{1}H\}$ NMR (162 MHz, DMSO-d_6) spectrum of complex **1**

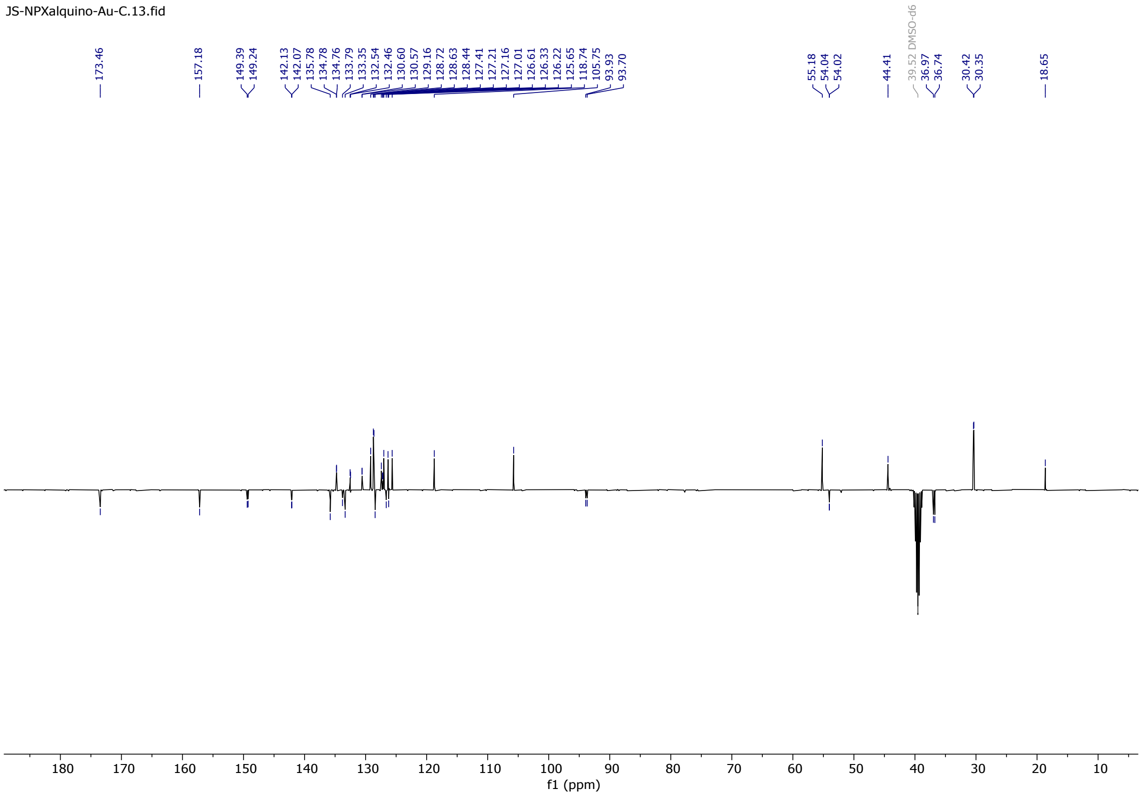

Figure S21. $^{13}C\{^1H\}$ NMR (101 MHz, $CDCl_3$) spectrum of complex **1.**

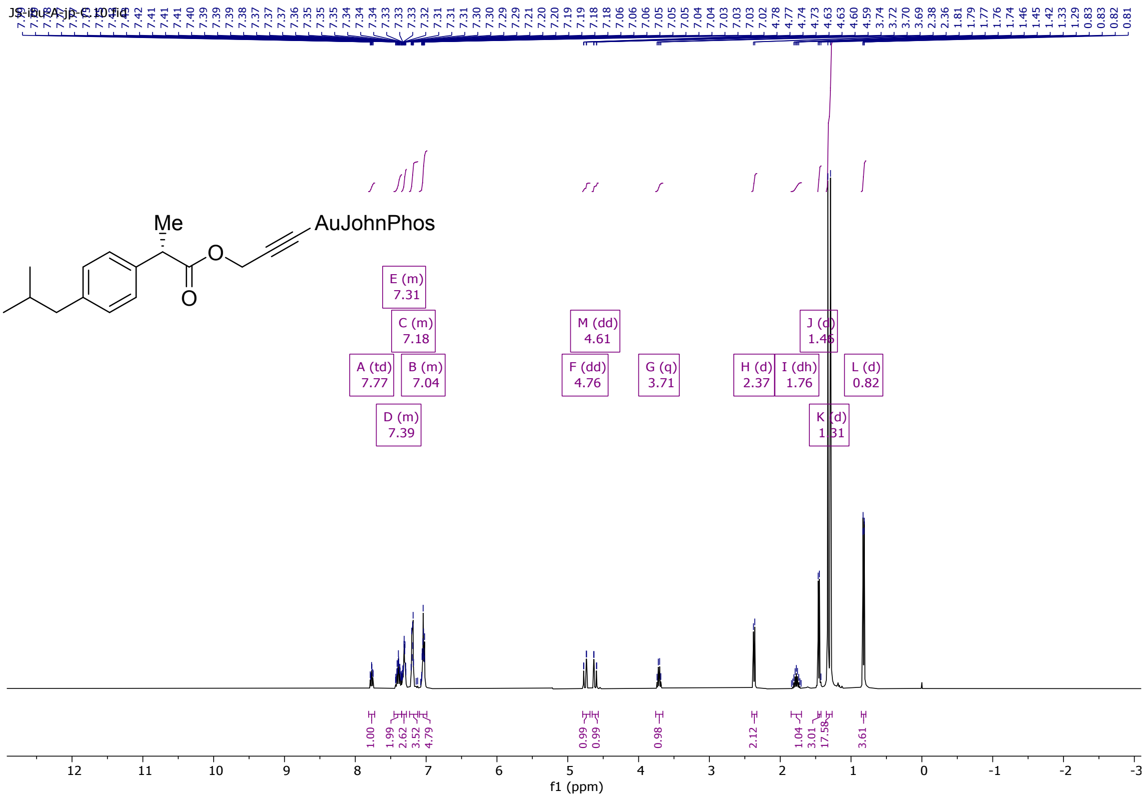

Figure S22. ^1H NMR Spectrum (400 MHz, $CDCl_3$) of complex **2.**

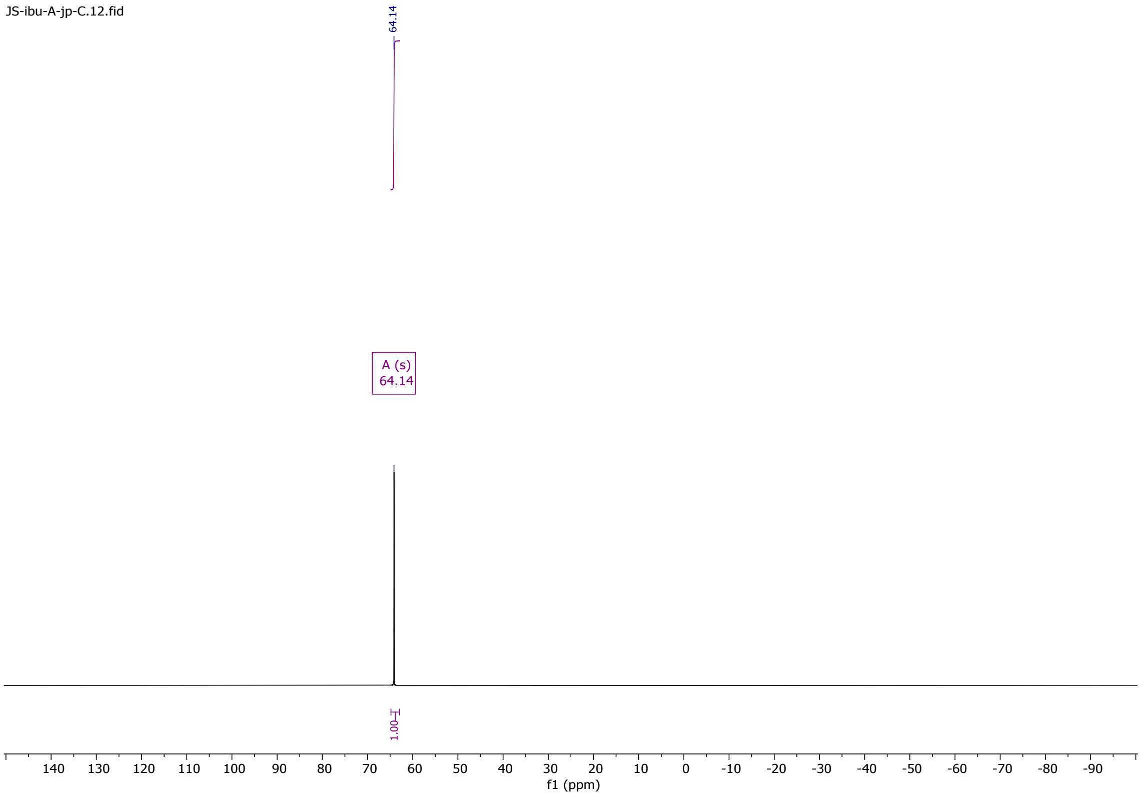

Figure S23. $^{31}P\{^{1}H\}$ NMR (162 MHz, $CDCl_3$) spectrum of complex **2**

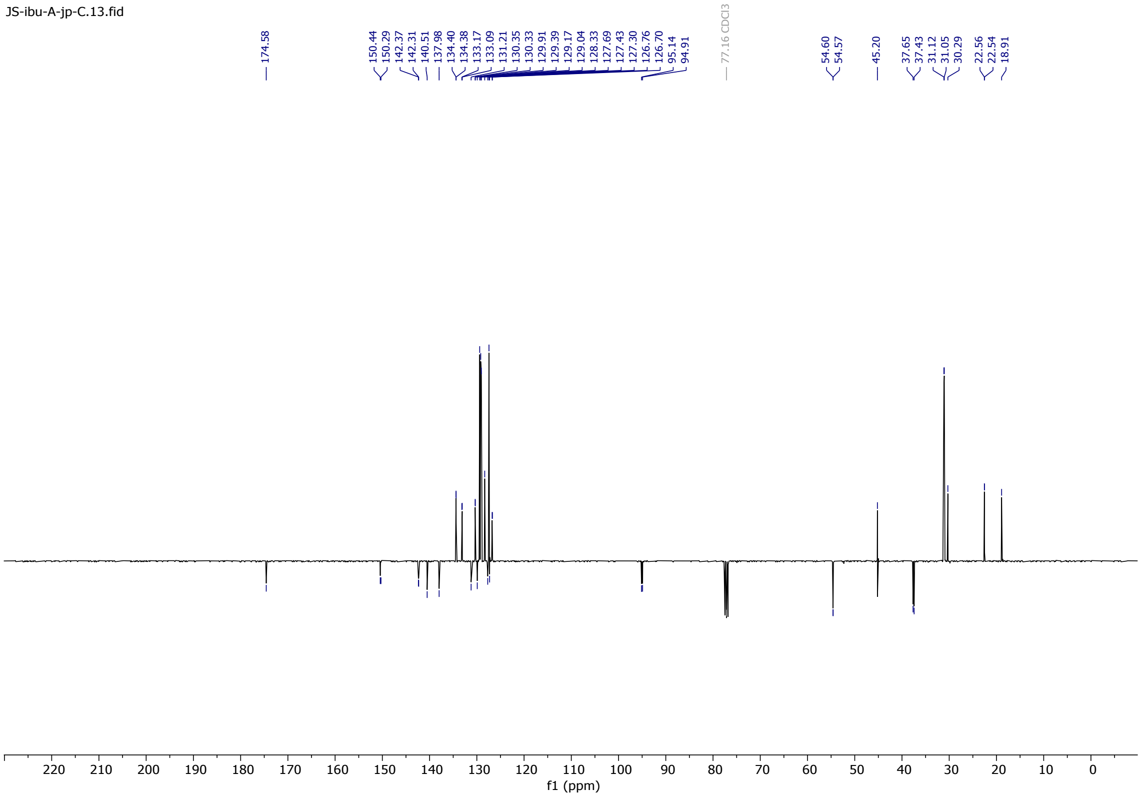

Figure S24. $^{13}C\{^{1}H\}$ NMR (101 MHz, $CDCl_3$) spectrum of complex **2.**

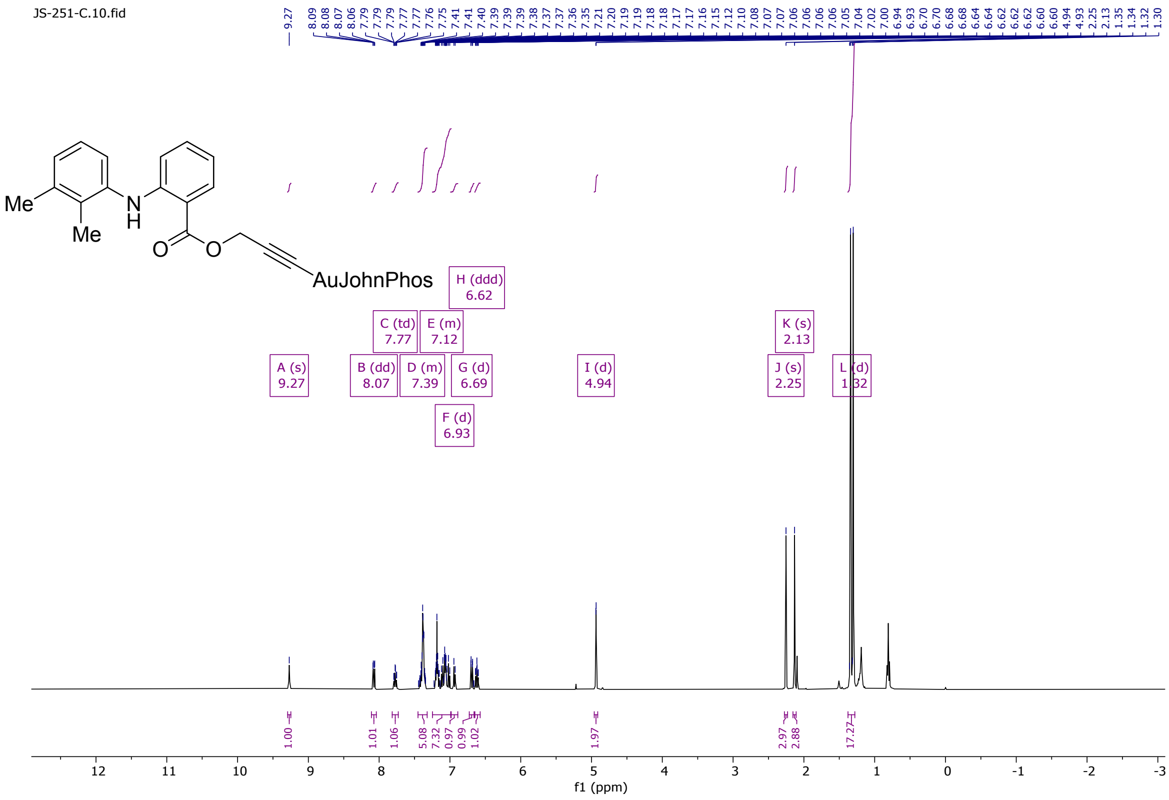

Figure S25. ^{1}H NMR Spectrum (400 MHz, $CDCl_3$) of complex **3.**

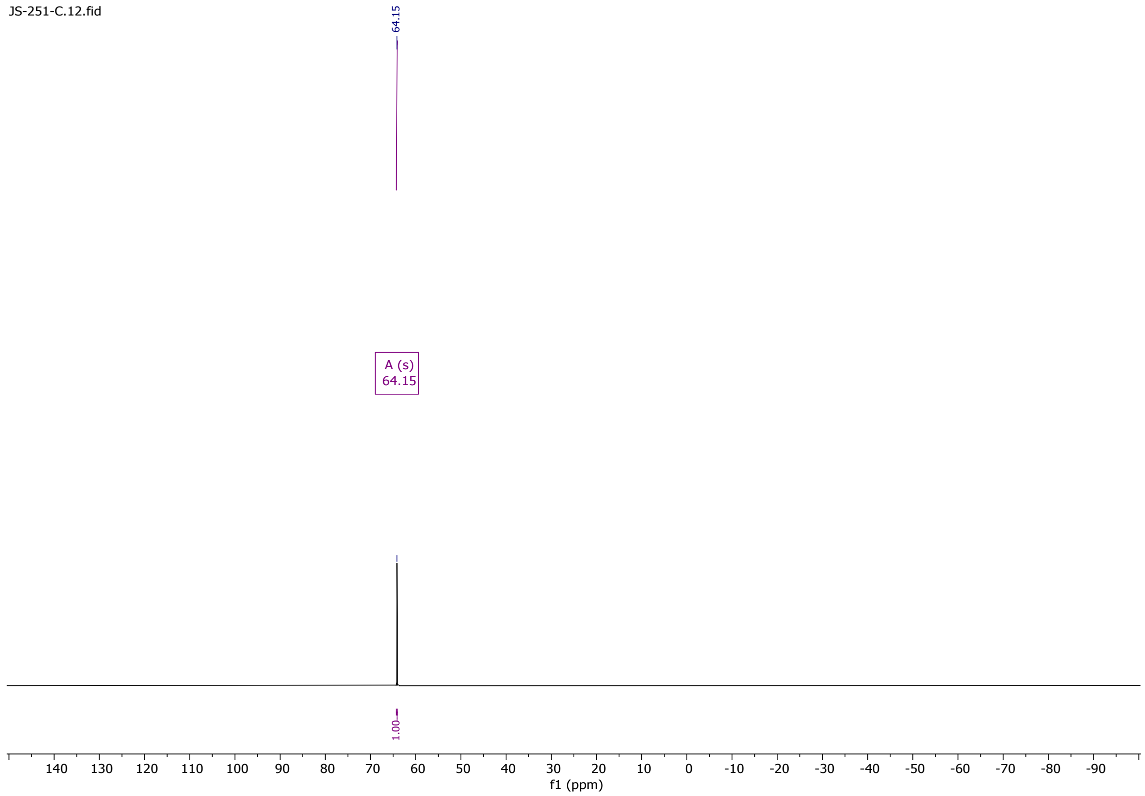

Figure S26. $^{31}P\{^{1}H\}$ NMR (162 MHz, $CDCl_3$) of complex **3.**

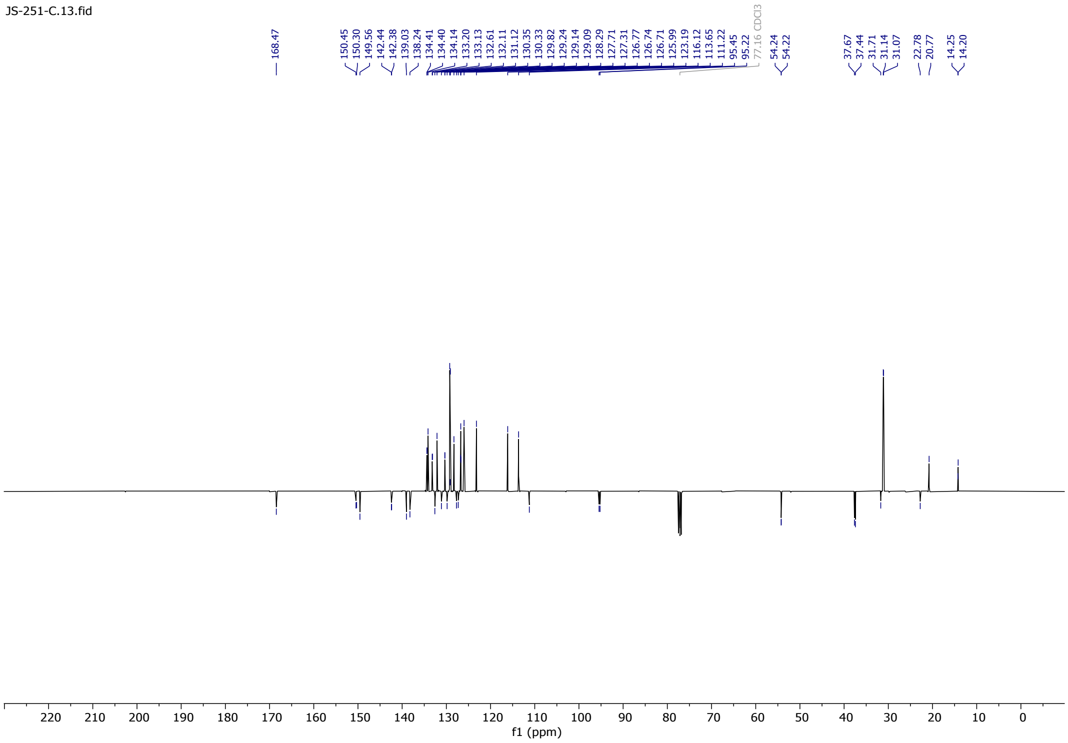

Figure S27. $^{13}C\{^1H\}$ NMR (101 MHz, $CDCl_3$) spectrum of complex **3.**

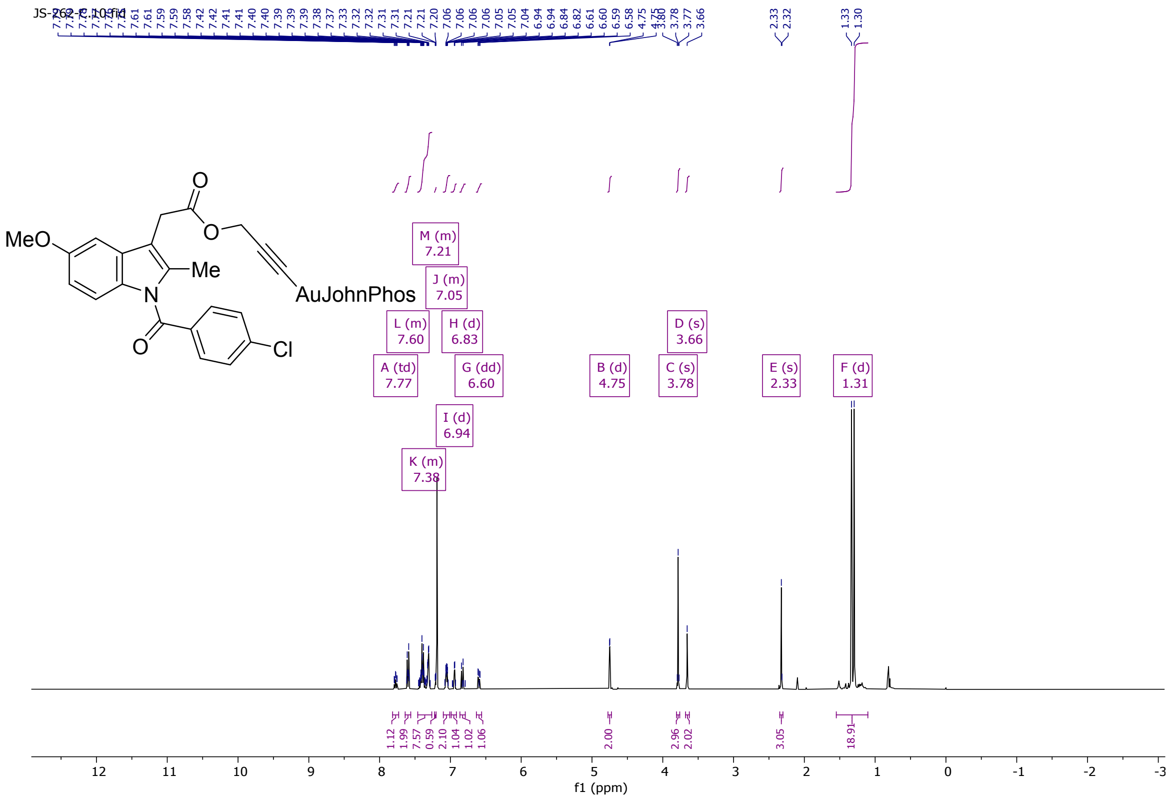

Figure S28. ^1H NMR Spectrum (400 MHz, $CDCl_3$) of complex **4.**

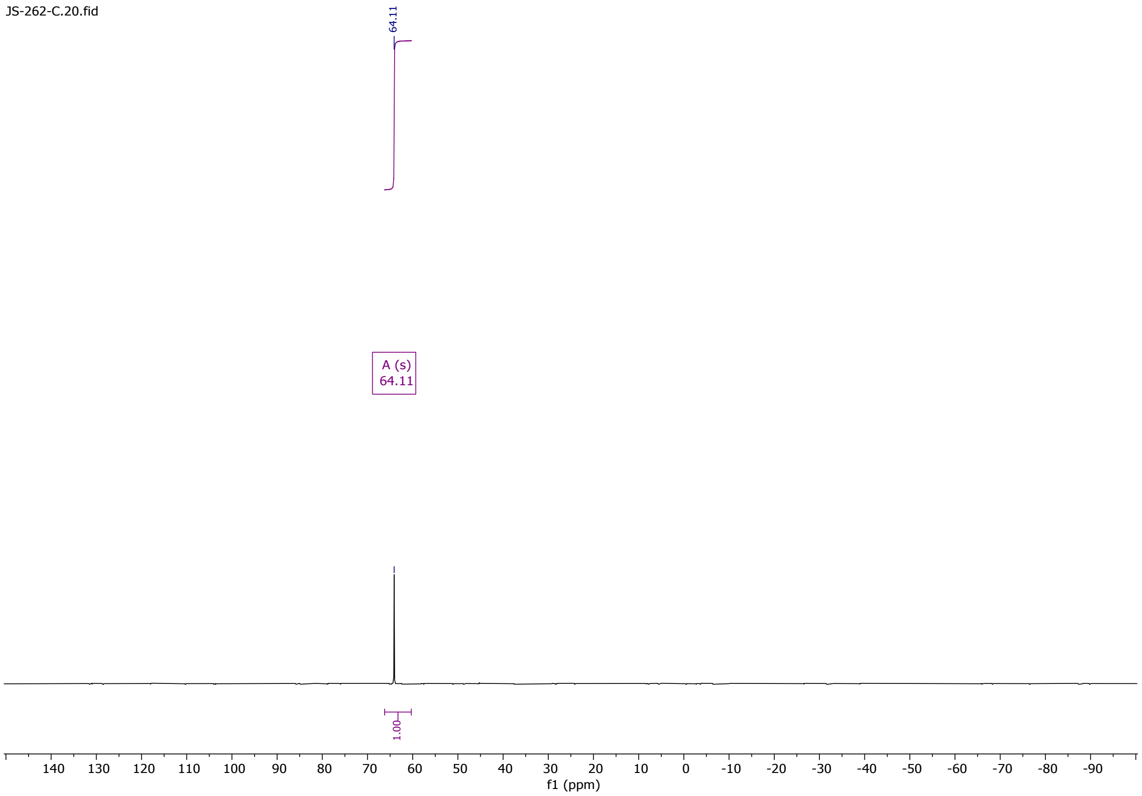

Figure S29. $^{31}P\{^{1}H\}$ NMR spectrum (162 MHz, $CDCl_3$) of complex **4.**

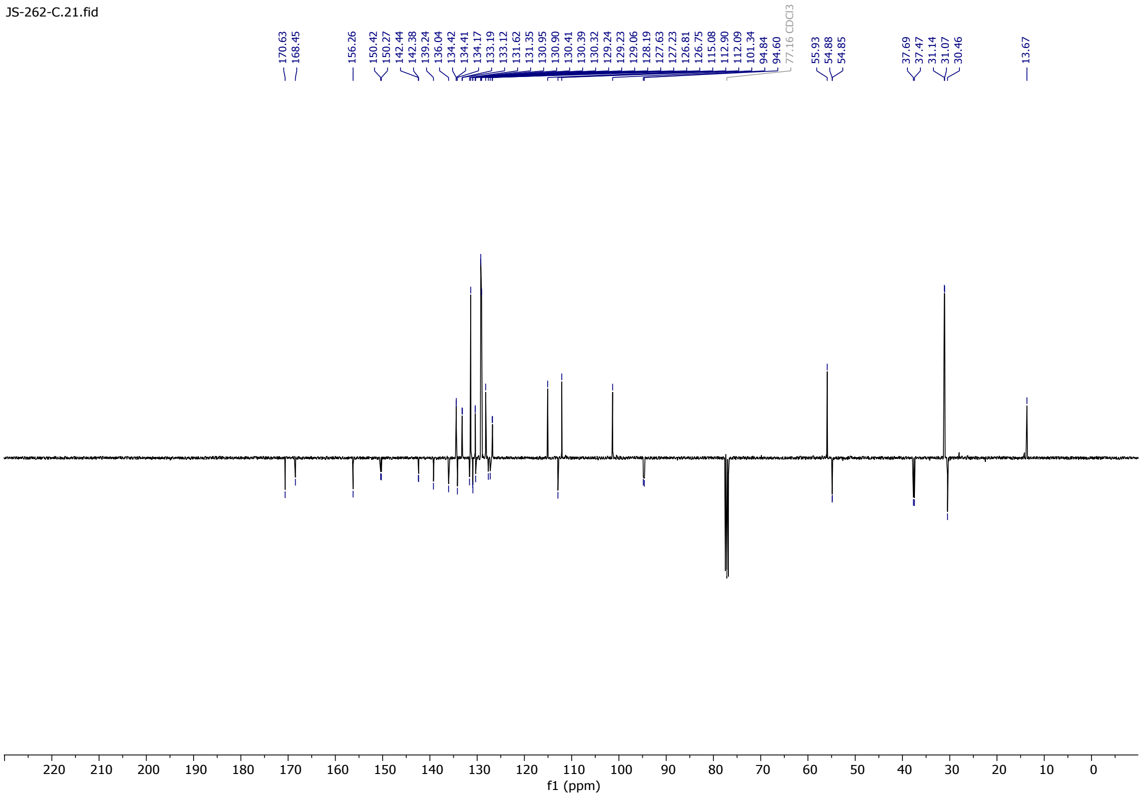

Figure S30. $^{13}C\{^{1}H\}$ NMR spectrum (101 MHz, $CDCl_3$) of complex **4.**

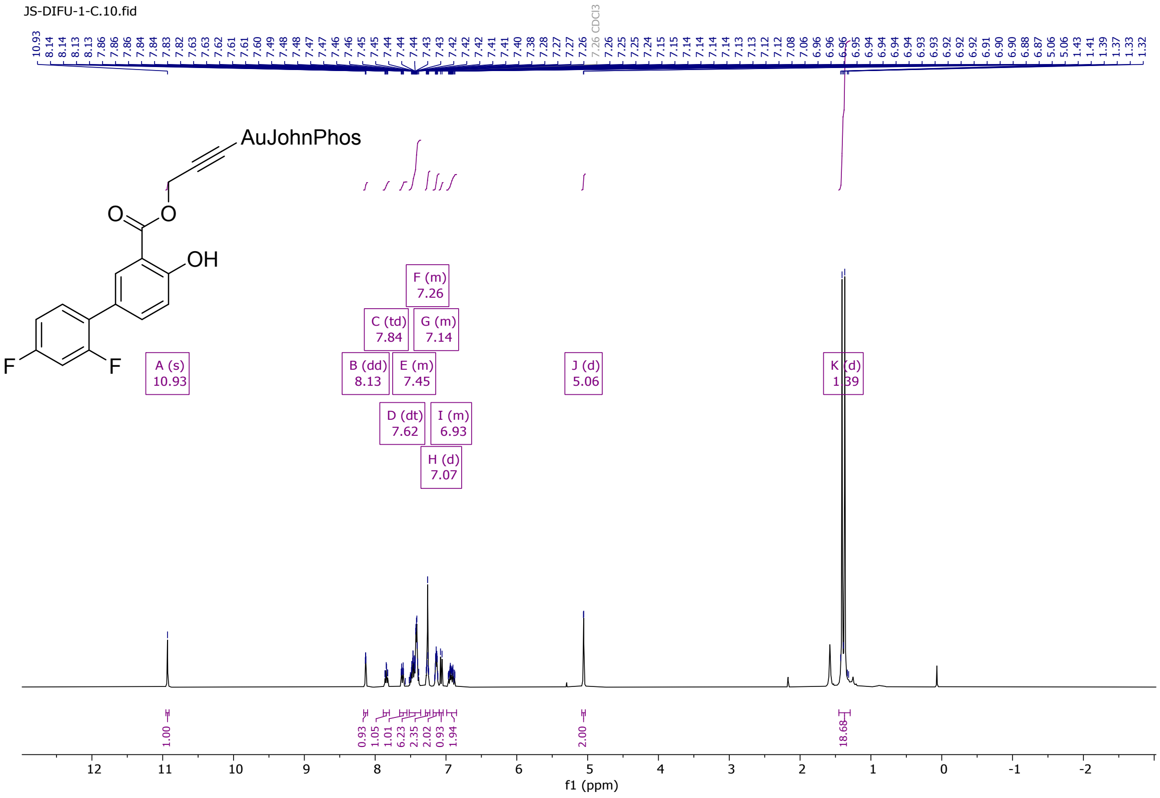

Figure S31. ^{1}H NMR Spectrum (400 MHz, $CDCl_3$) of complex **5**

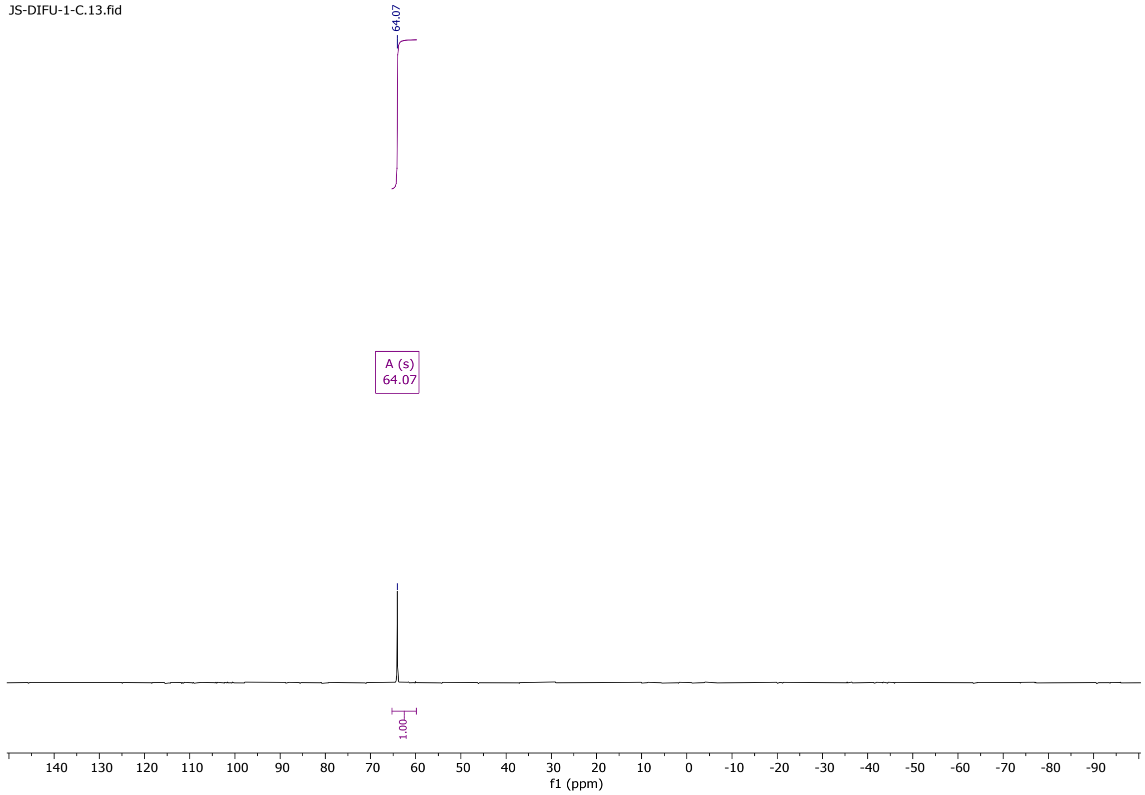

Figure S32. $^{31}P\{^{1}H\}$ NMR spectrum (162 MHz, $CDCl_3$) of complex **5**

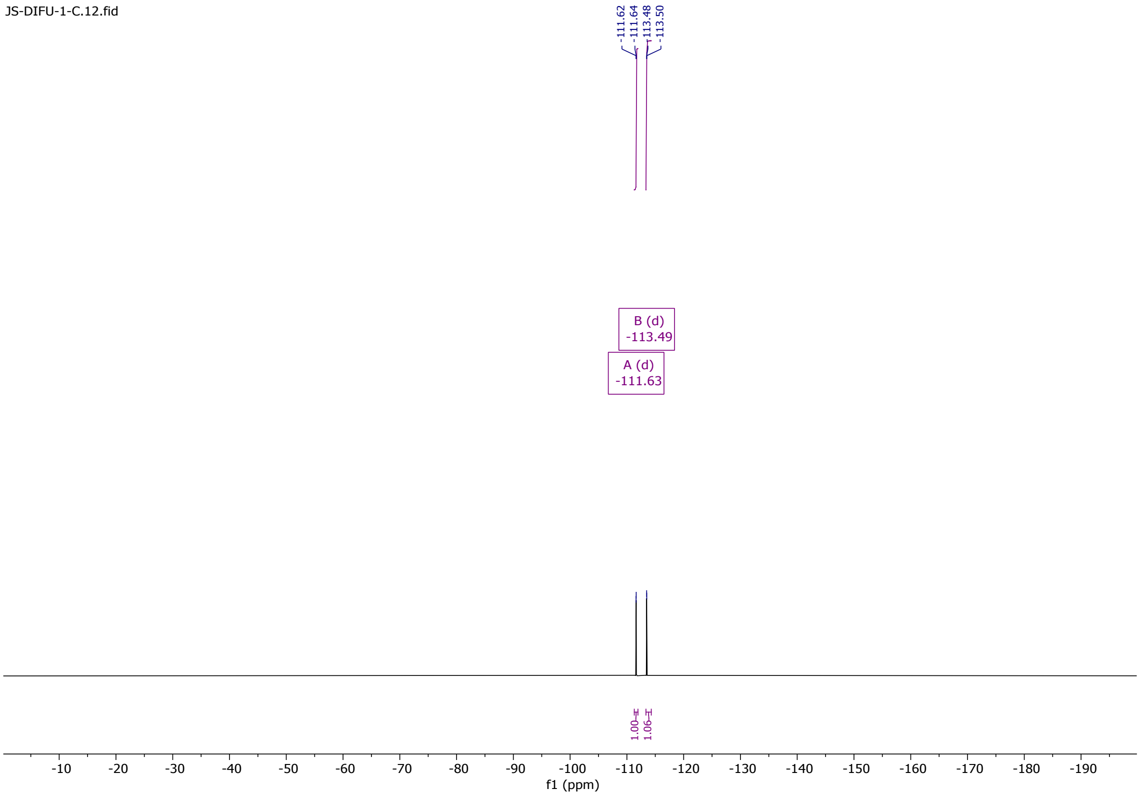

Figure S33. $^{19}F\{^1H\}$ NMR spectrum (376 MHz, $CDCl_3$) of complex **5.**

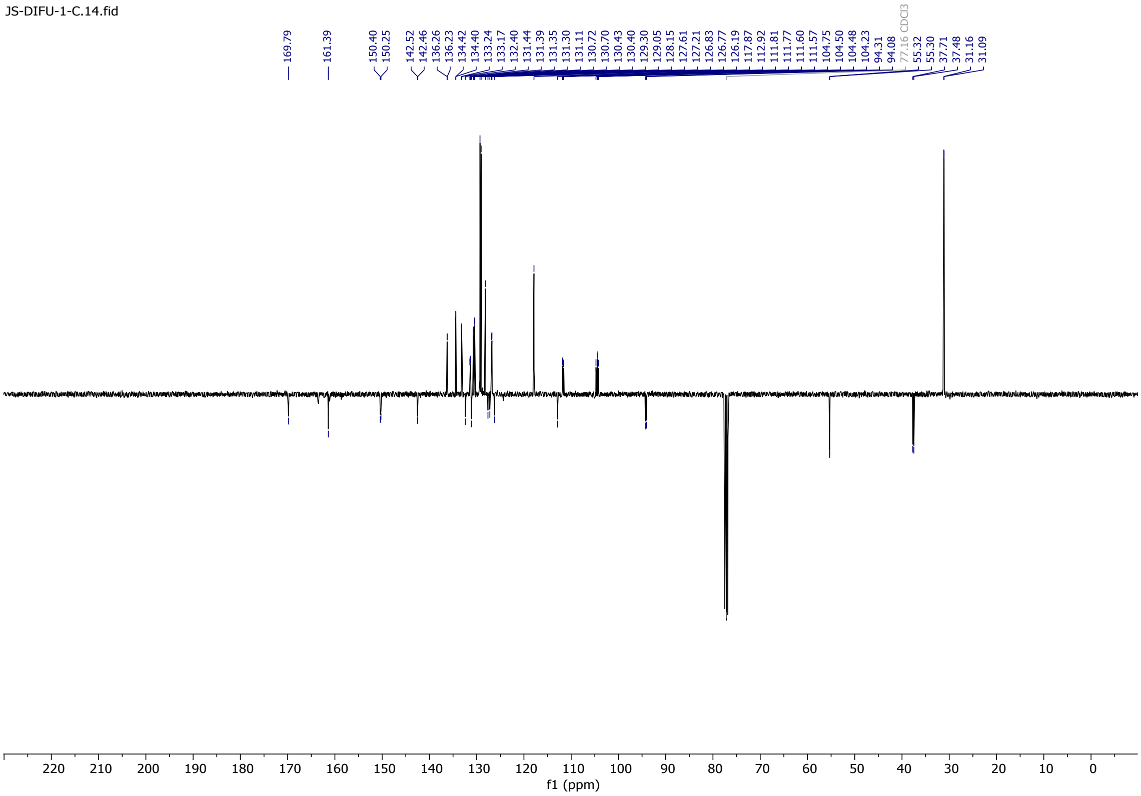

Figure S34. $^{13}C\{^1H\}$ NMR spectrum (101 MHz, $CDCl_3$) of complex **5**

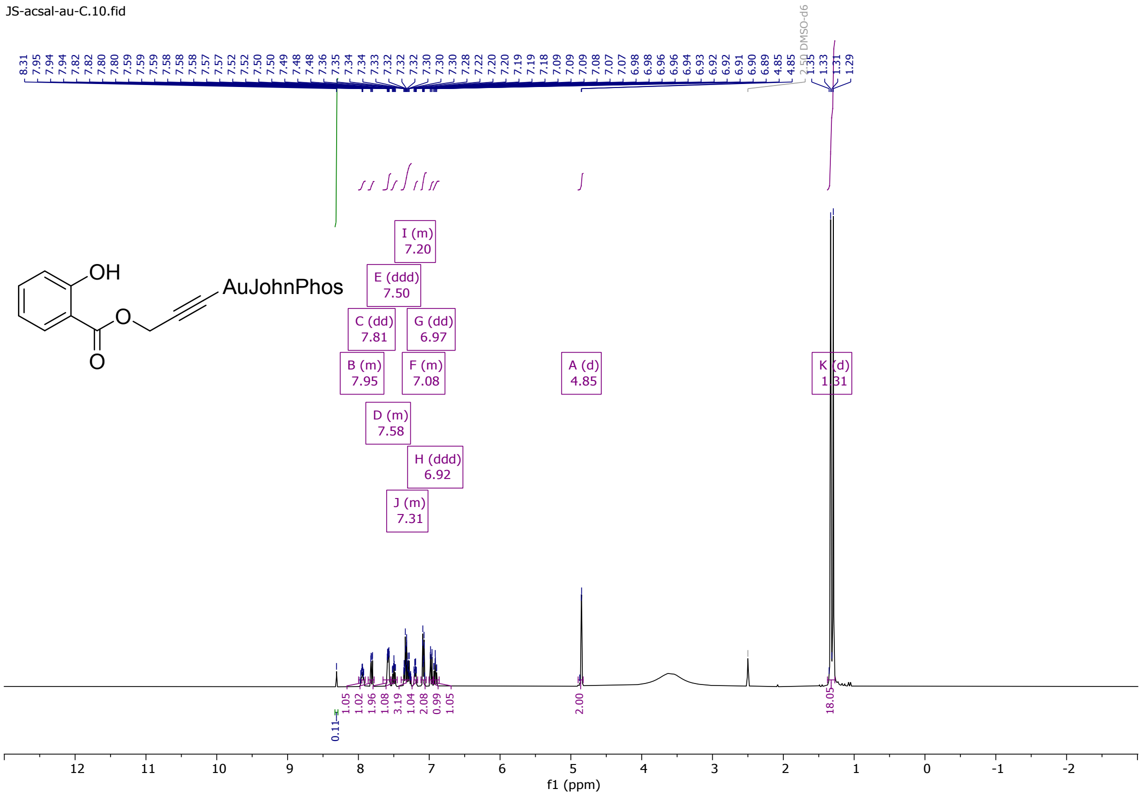

Figure S35. ^{1}H NMR Spectrum (400 MHz, DMSO-d$_6$) of complex **6.**

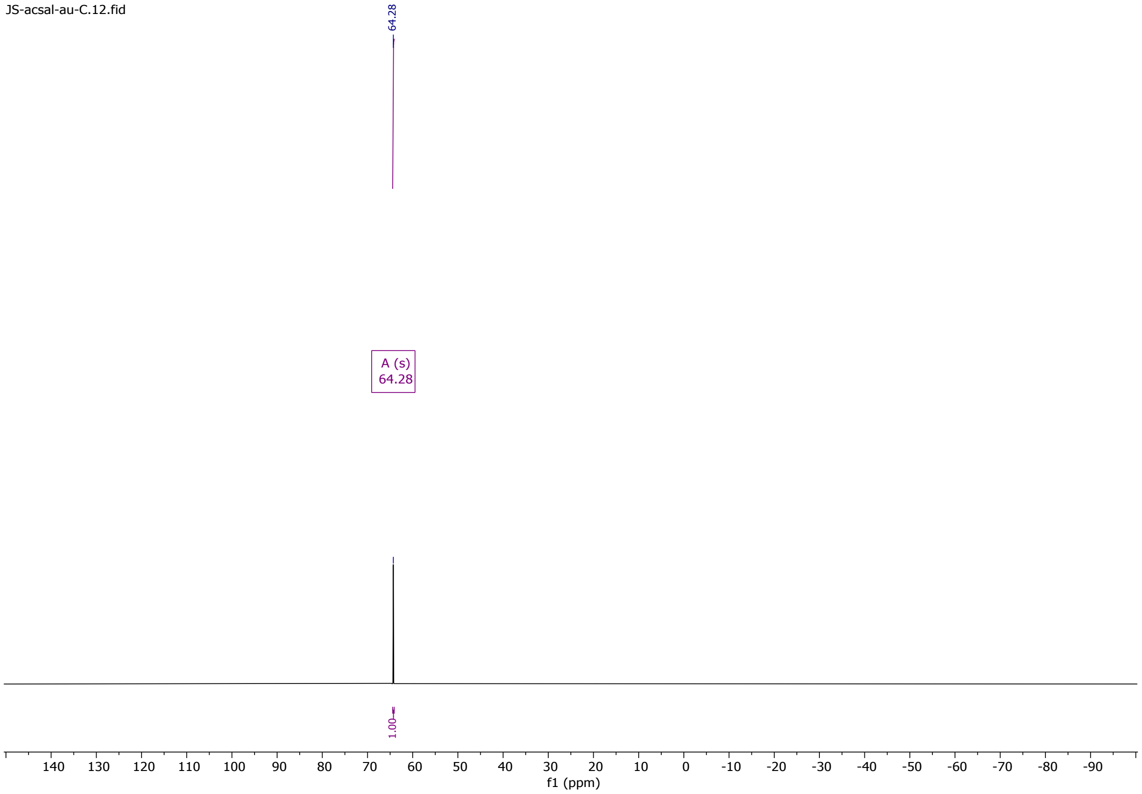

Figure S36. ^{31}P{^{1}H} NMR spectrum (162 MHz, DMSO-d$_6$) of complex **6**

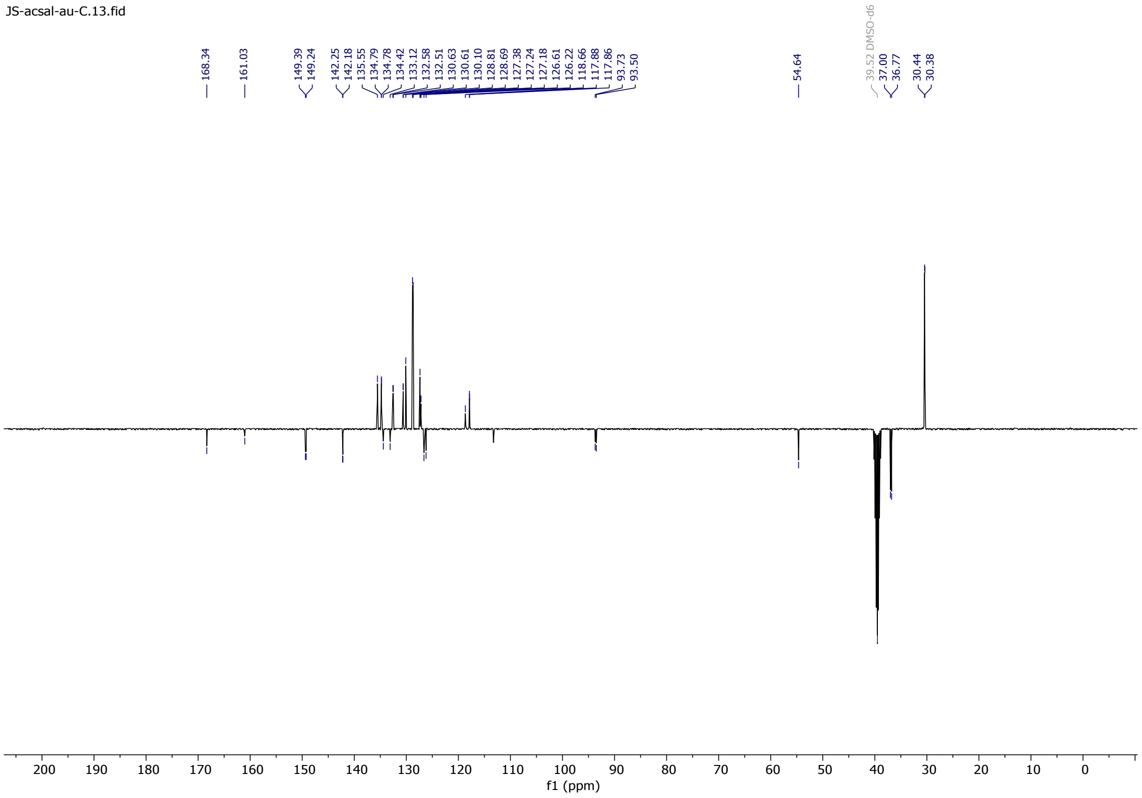

Figure S37. $^{13}C\{^{1}H\}$ NMR spectrum (101 MHz, DMSO-d$_6$) of complex **6**

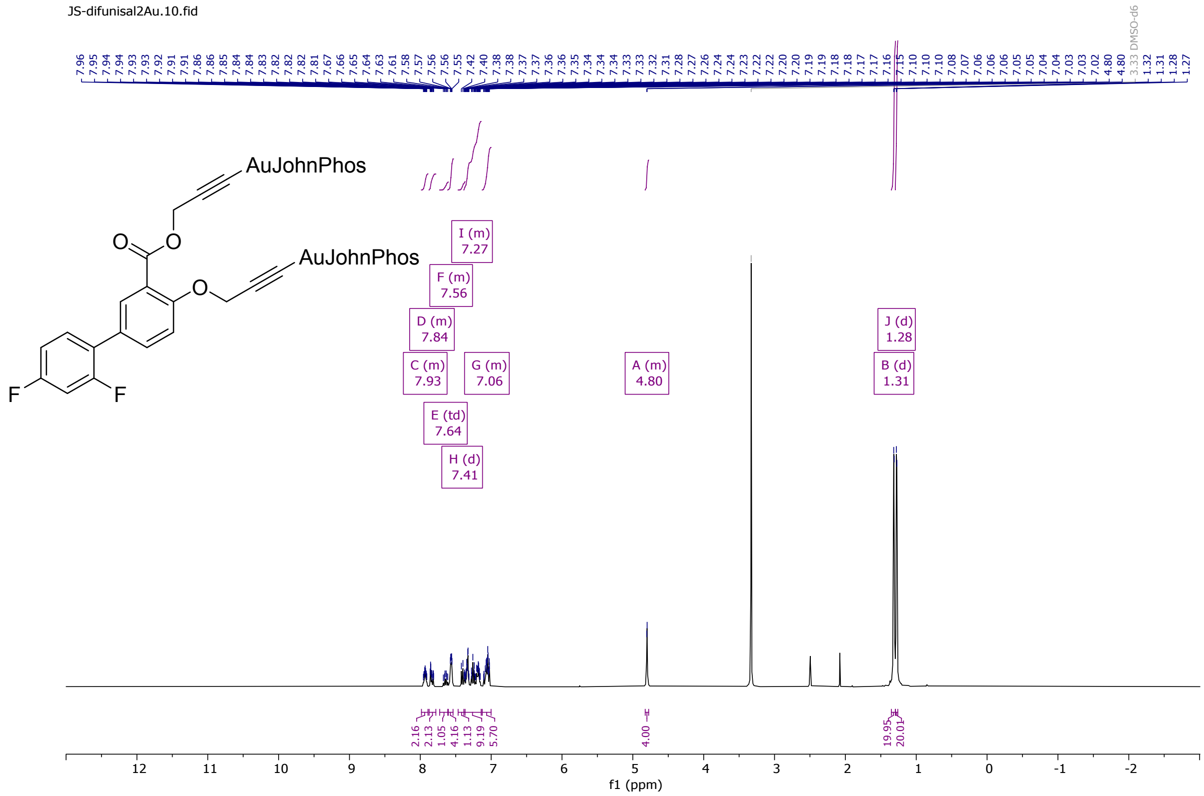

Figure S38. ^{1}H NMR spectrum (400 MHz, DMSO-d$_6$) of complex **7.**

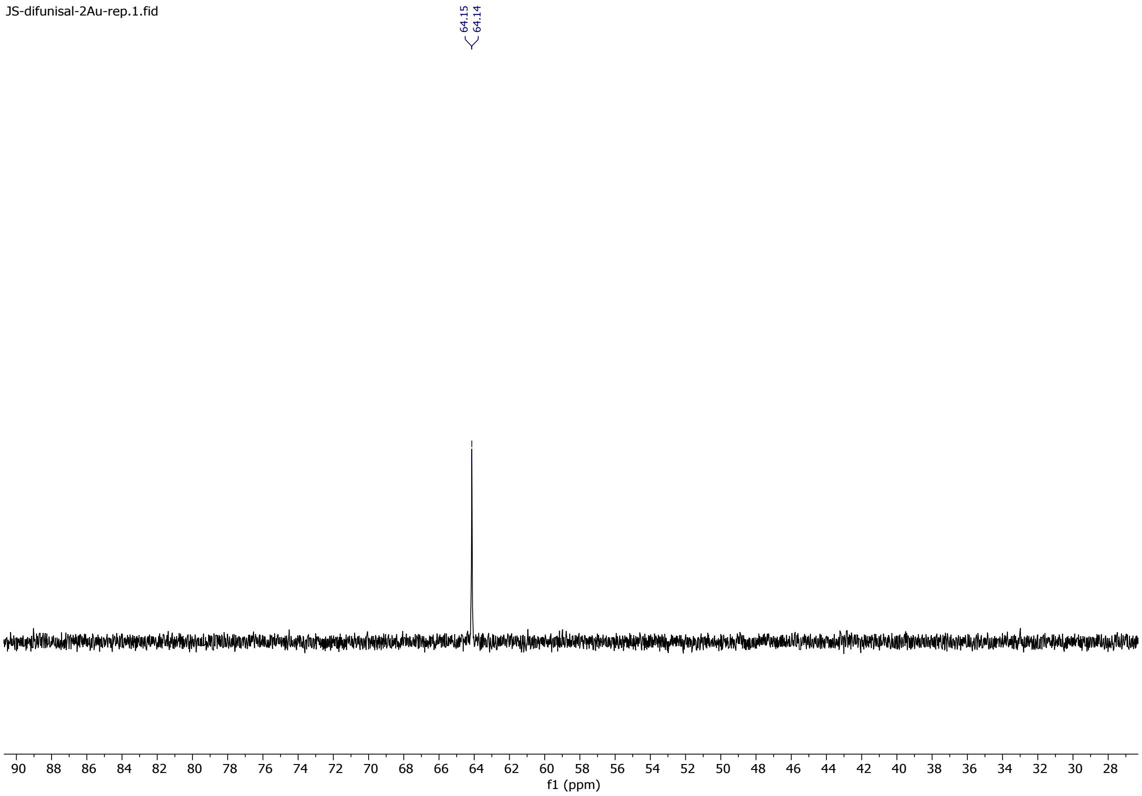

Figure S39. $^{31}P\{^{1}H\}$ NMR spectrum (162 MHz, DMSO-d_6) of complex **7**

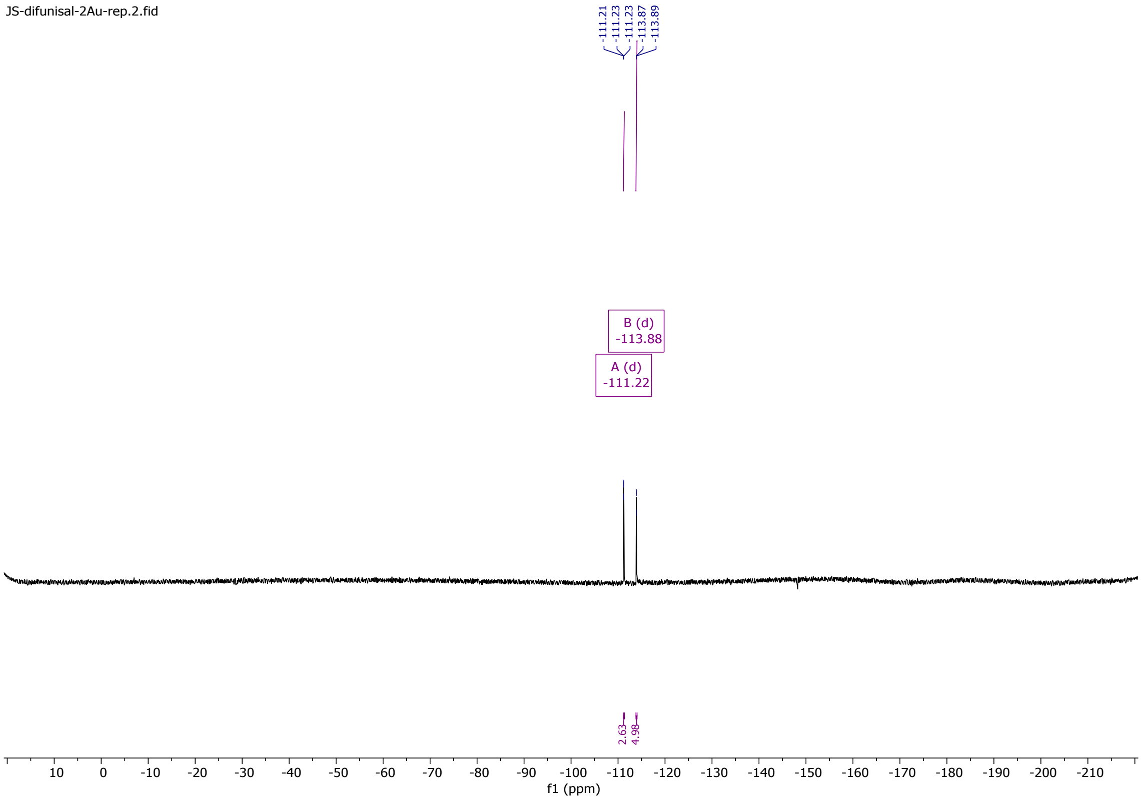

Figure S 40. $^{19}F\{^{1}H\}$ NMR spectrum (376 MHz, DMSO-d_6) of complex **7.**

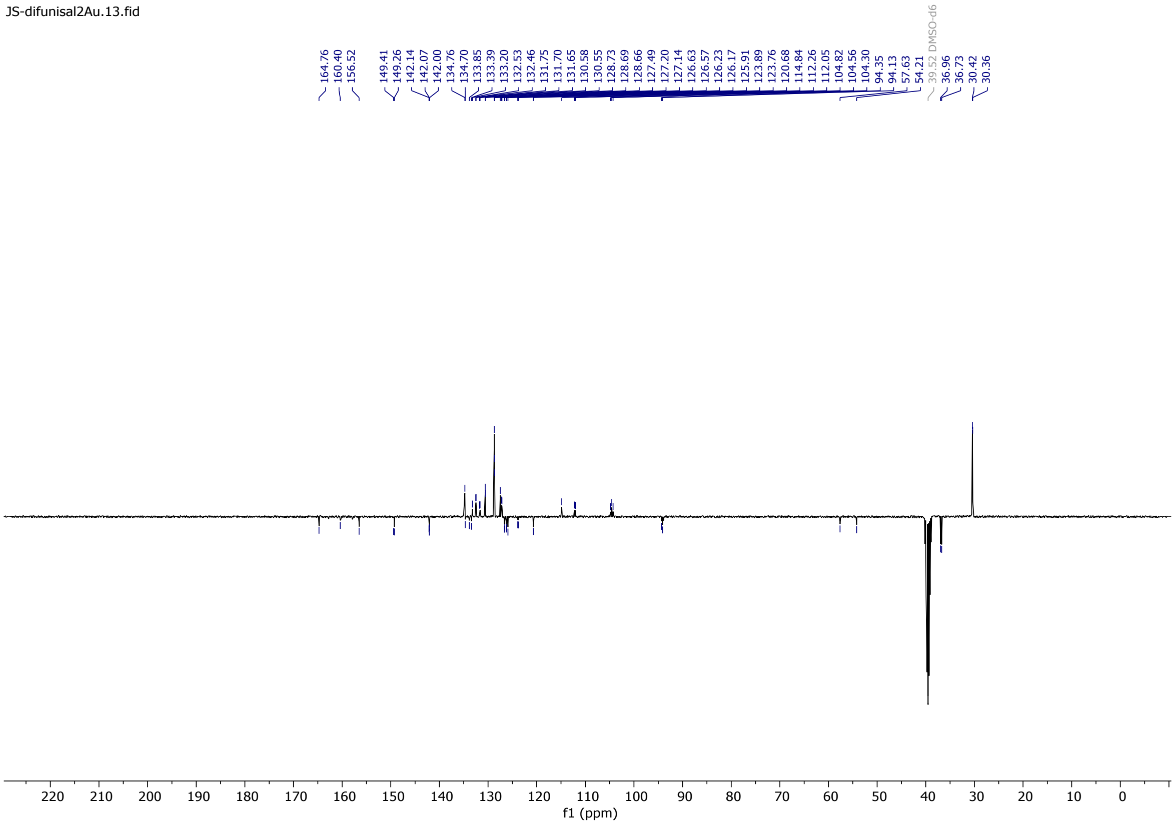

Figure S41. $^{13}C\{^1H\}$ NMR spectrum (101 MHz, DMSO-d$_6$) of complex **7**

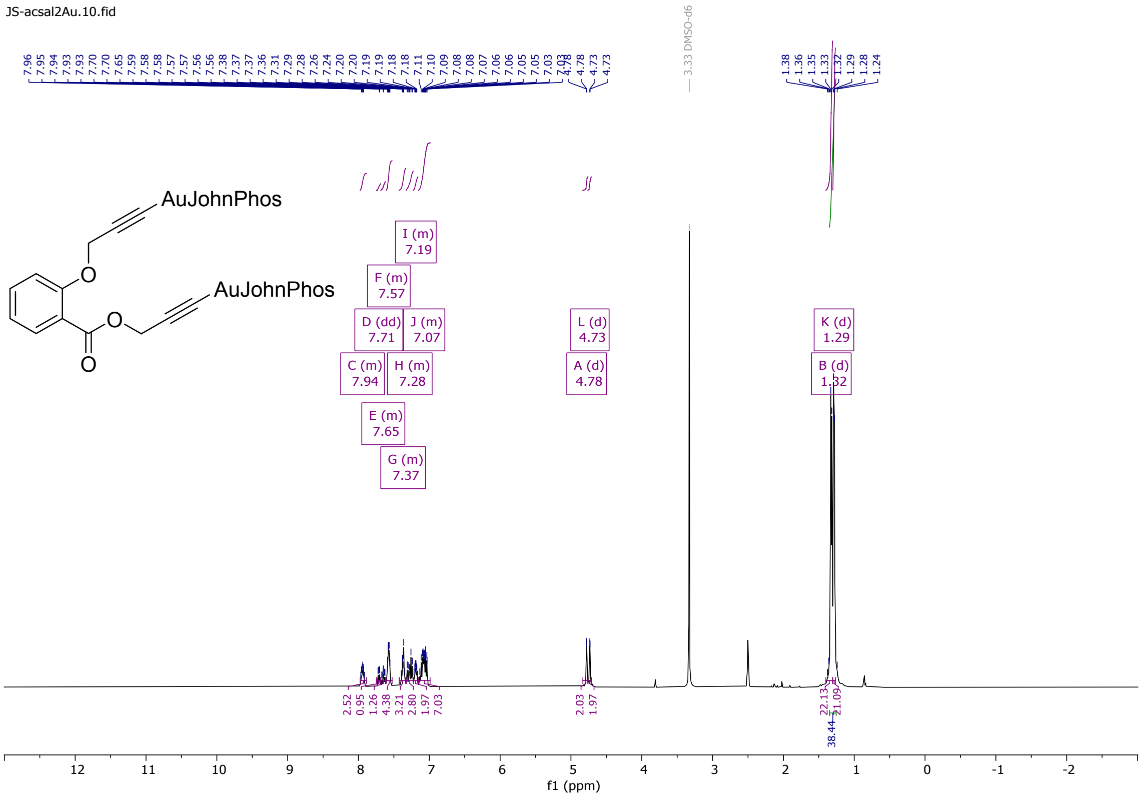

Figure S42. ^1H NMR Spectrum (400 MHz, DMSO-d$_6$) of complex **8.**

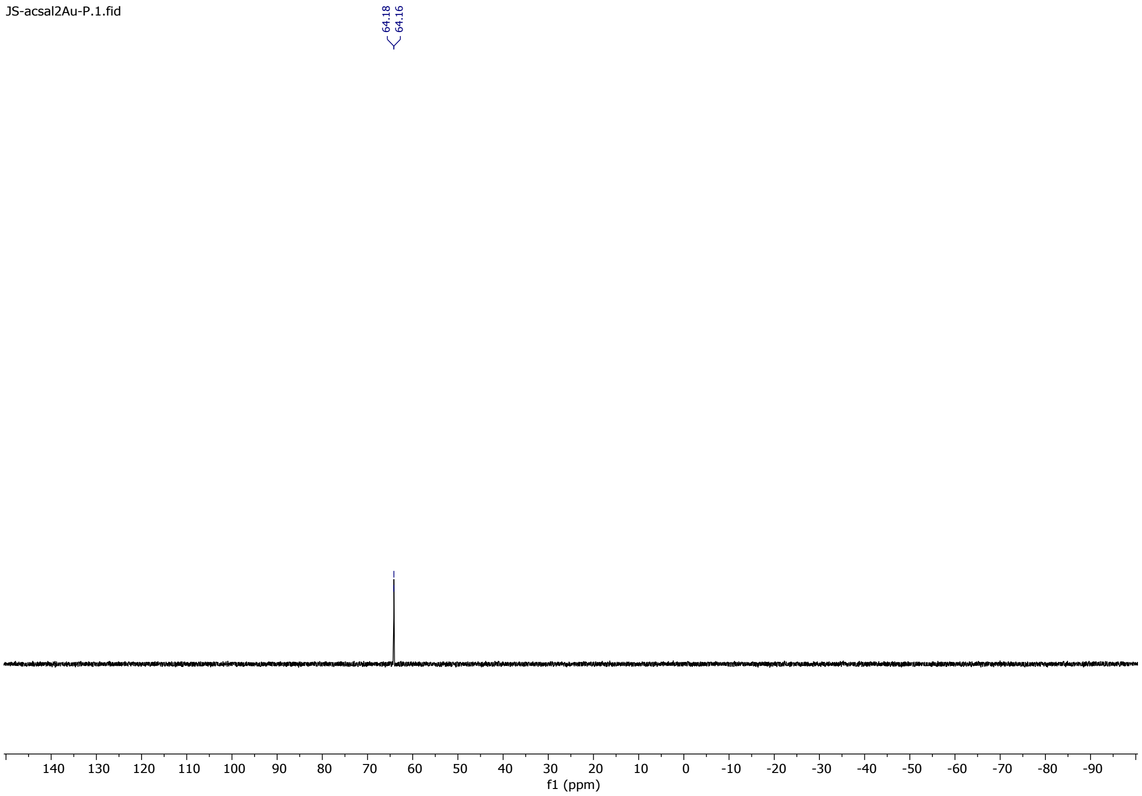

Figure S43. $^{31}P\{^{1}H\}$ NMR spectrum (162 MHz, DMSO-d$_6$) of complex **8.**

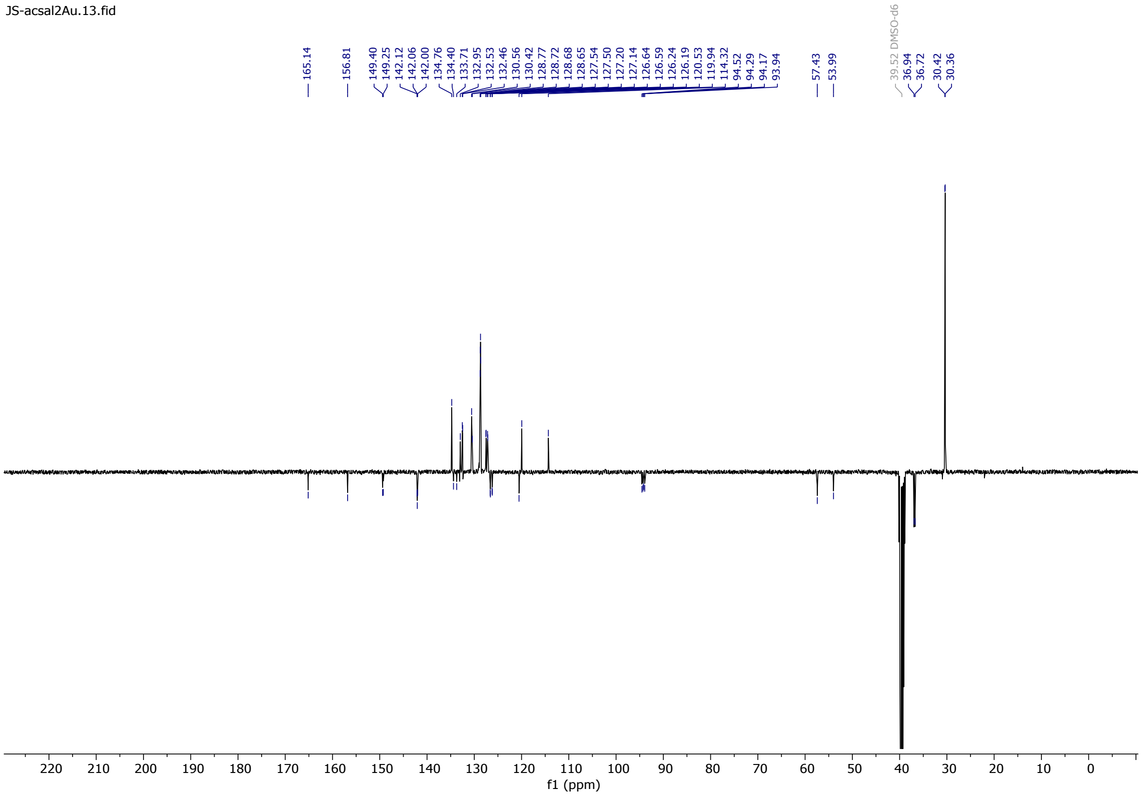

Figure S44. $^{13}C\{^{1}H\}$ NMR spectrum (101 MHz, DMSO-d$_6$) of complex **8.**

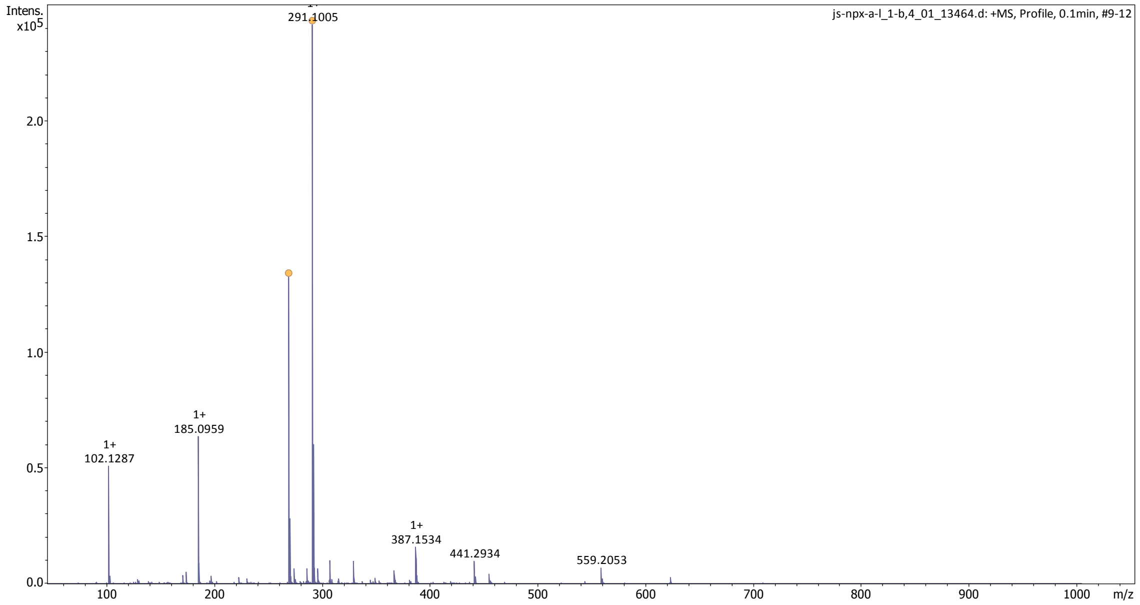

Figure S45. ESI-HRMS of **L-1.**

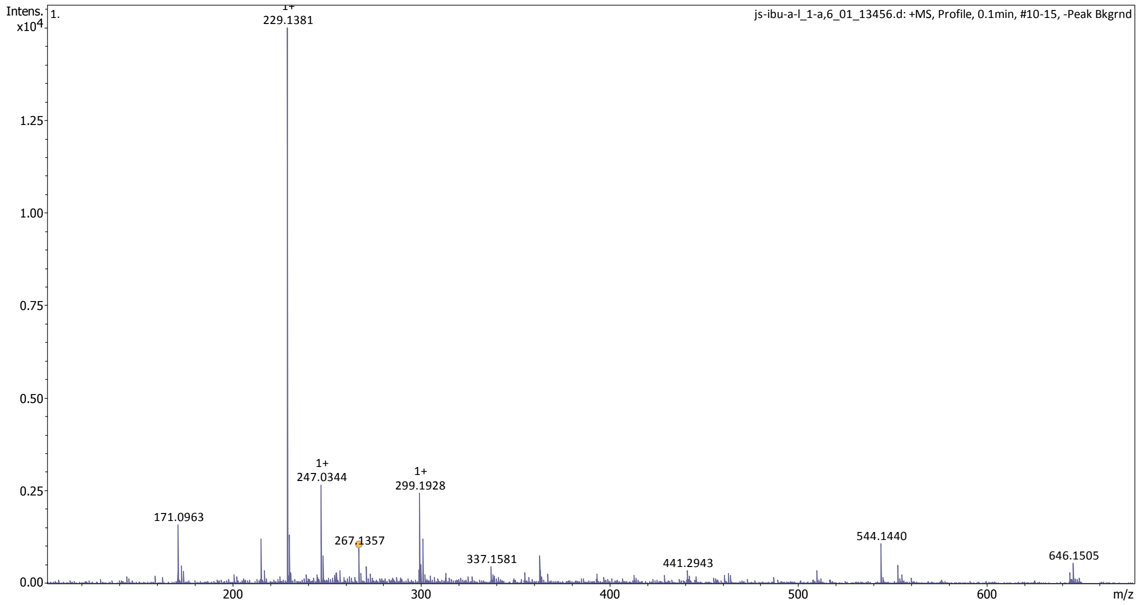

Figure S46. ESI-HRMS of **L-2.**

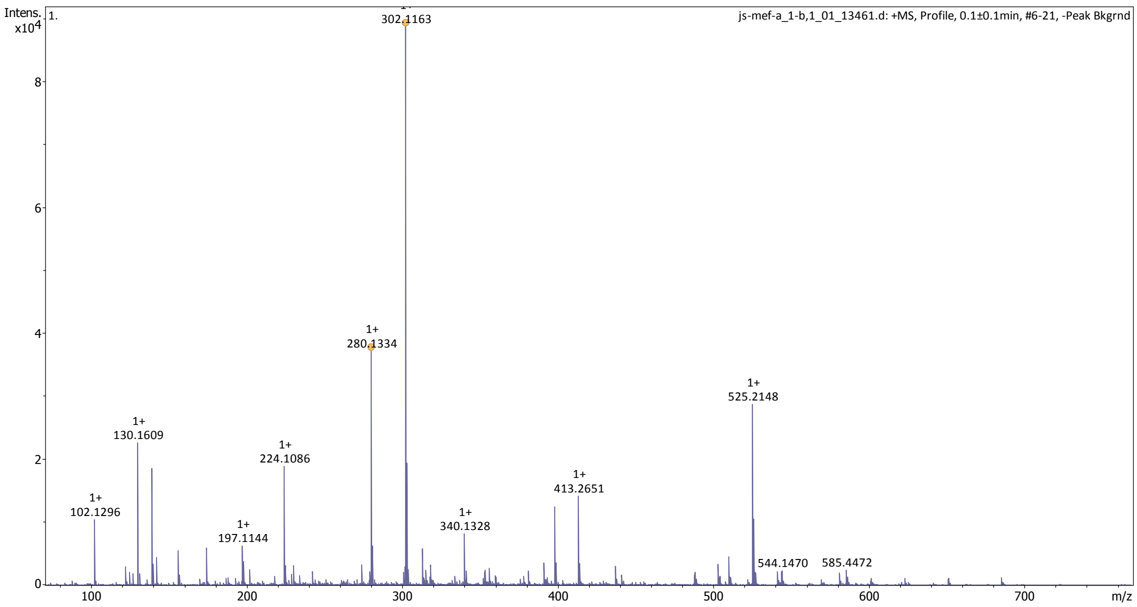

Figure S47. ESI-HRMS of **L-3.**

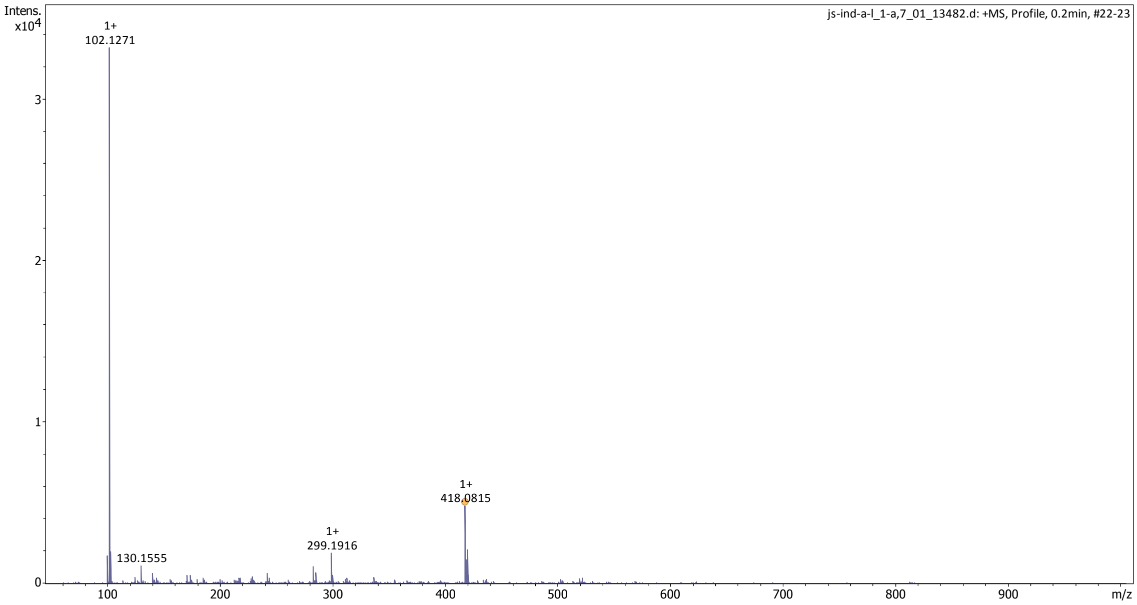

Figure S 48. ESI-HRMS of **L4.**

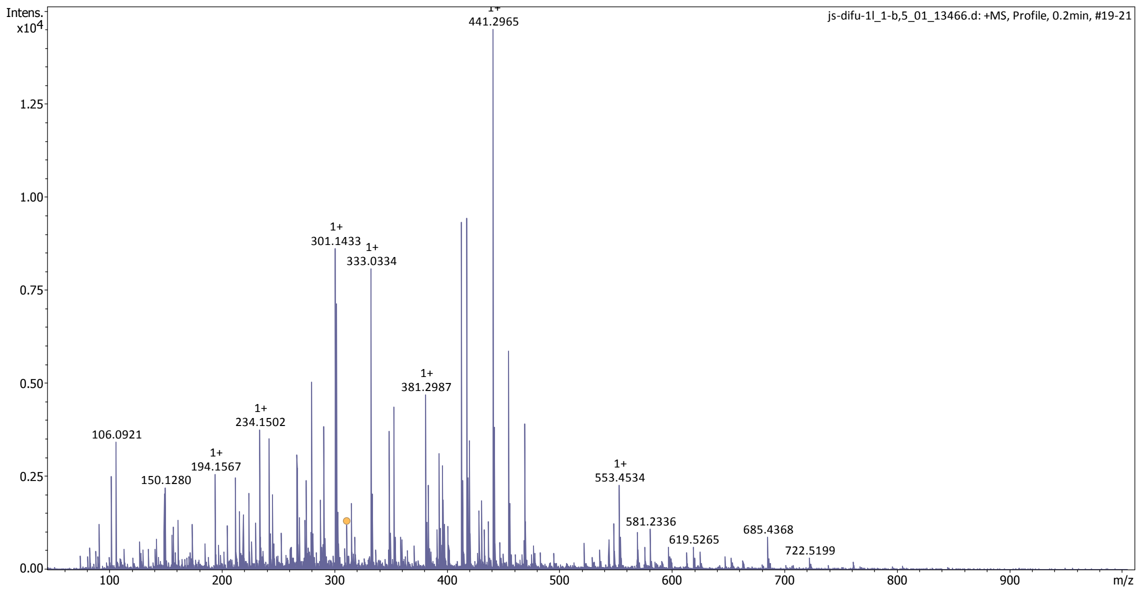

Figure S 49. ESI-HRMS of **L5.**

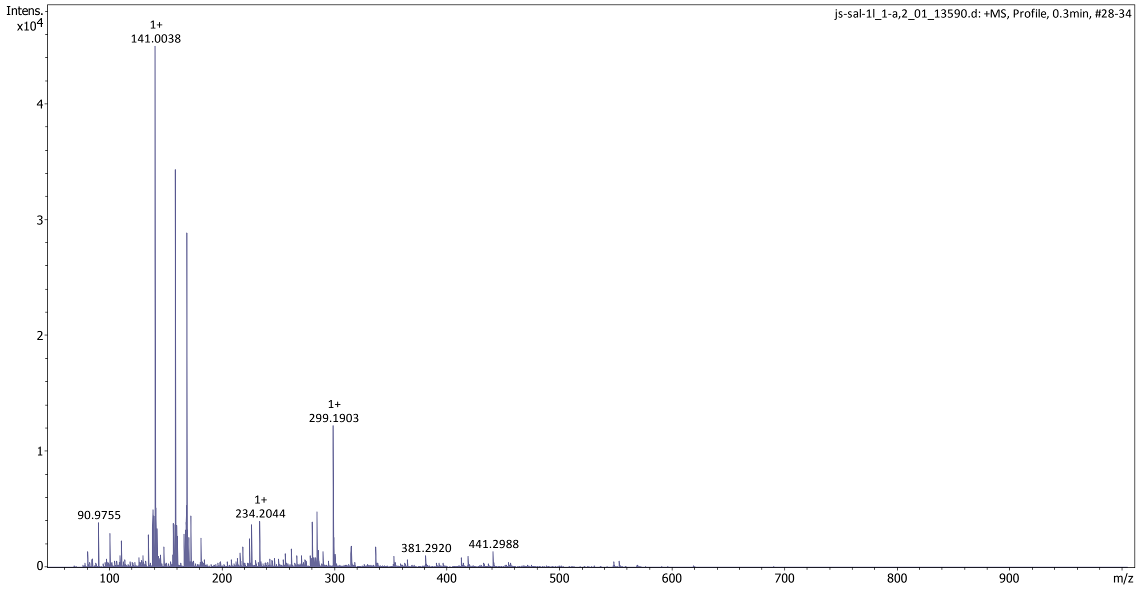

Figure S 50. ESI-HRMS of **L6.**

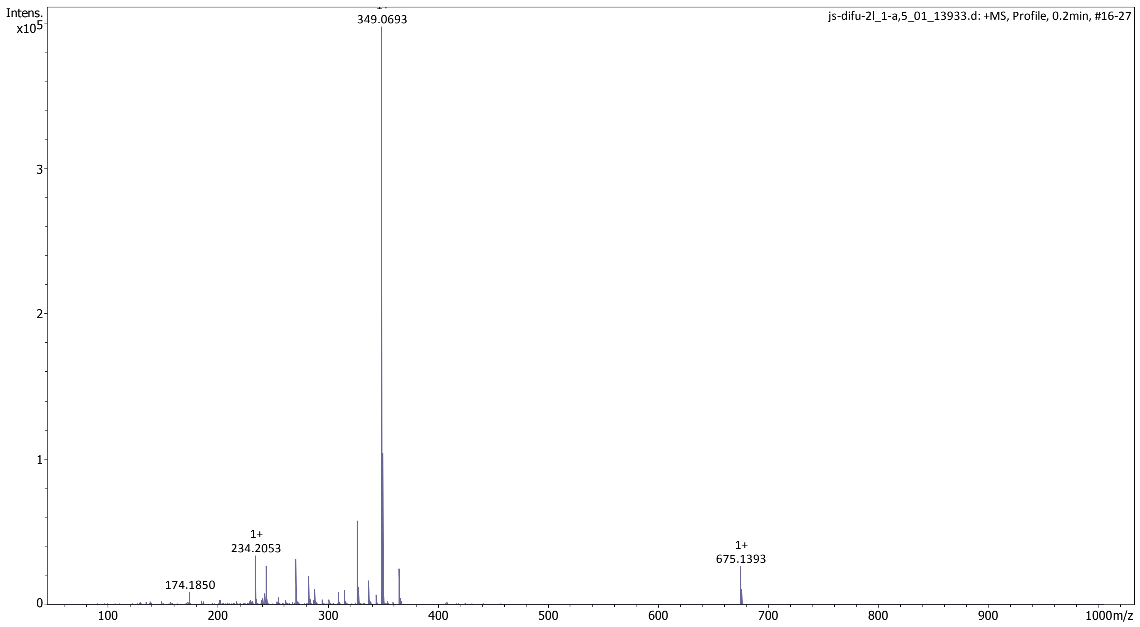

Figure S51. ESI-HRMS of **L7.**

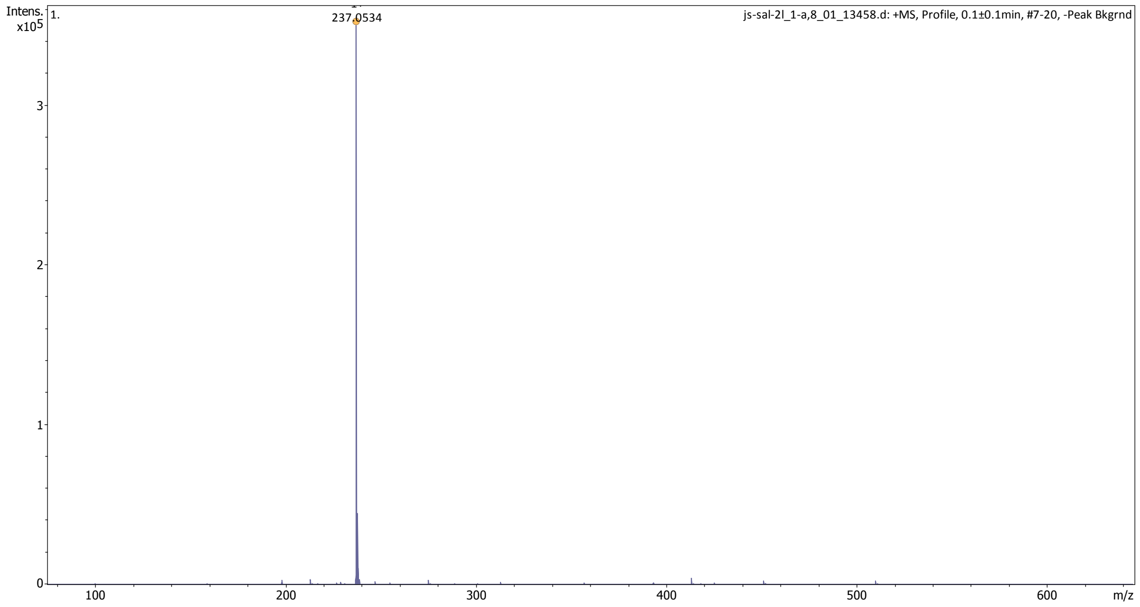

Figure S52. ESI-HRMS of **L8.**

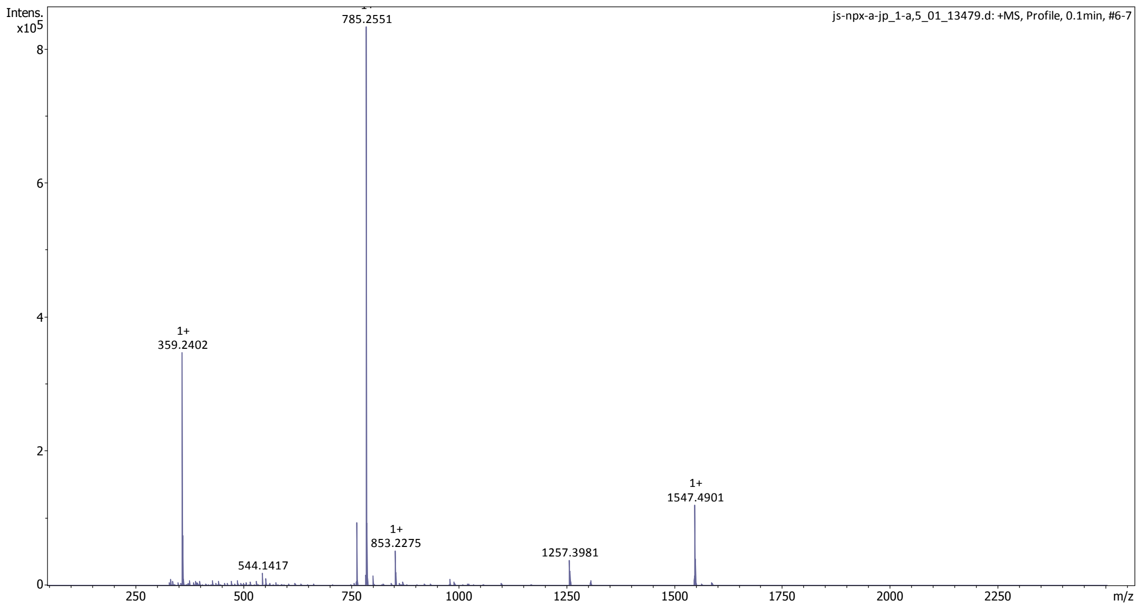

Figure S53. ESI-HRMS of complex **1.**

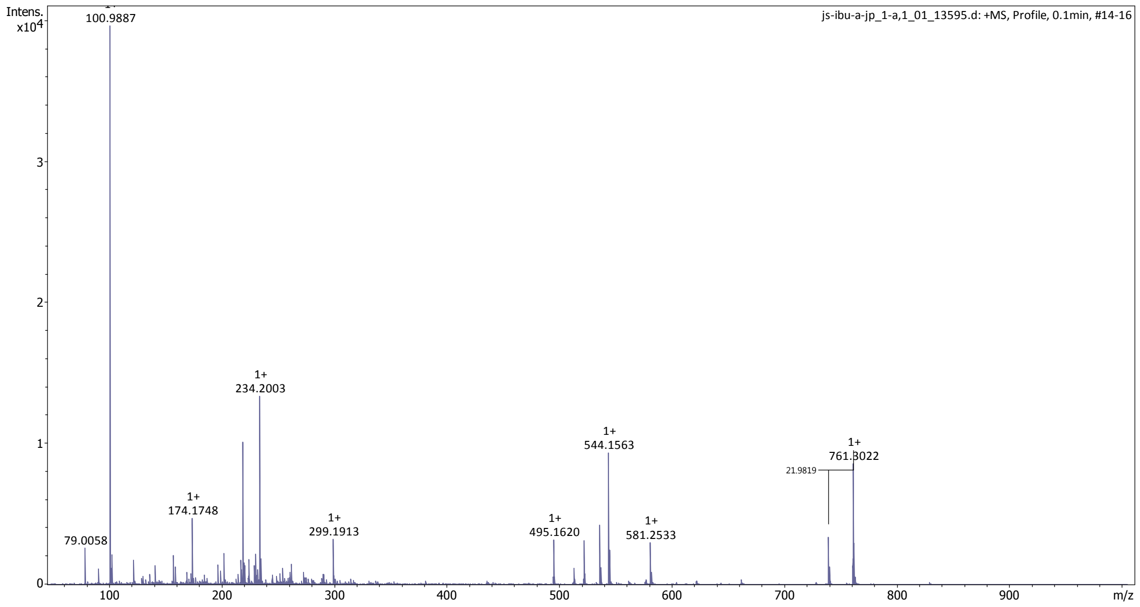

Figure S54. ESI-HRMS of complex **2.**

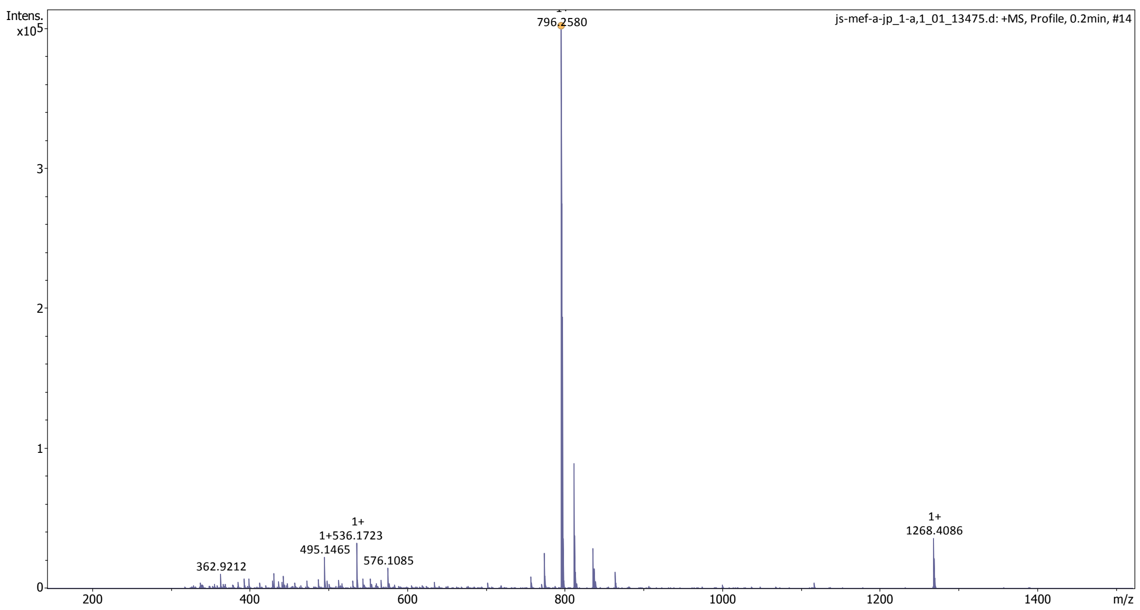

Figure S55. ESI-HRMS of complex **3.**

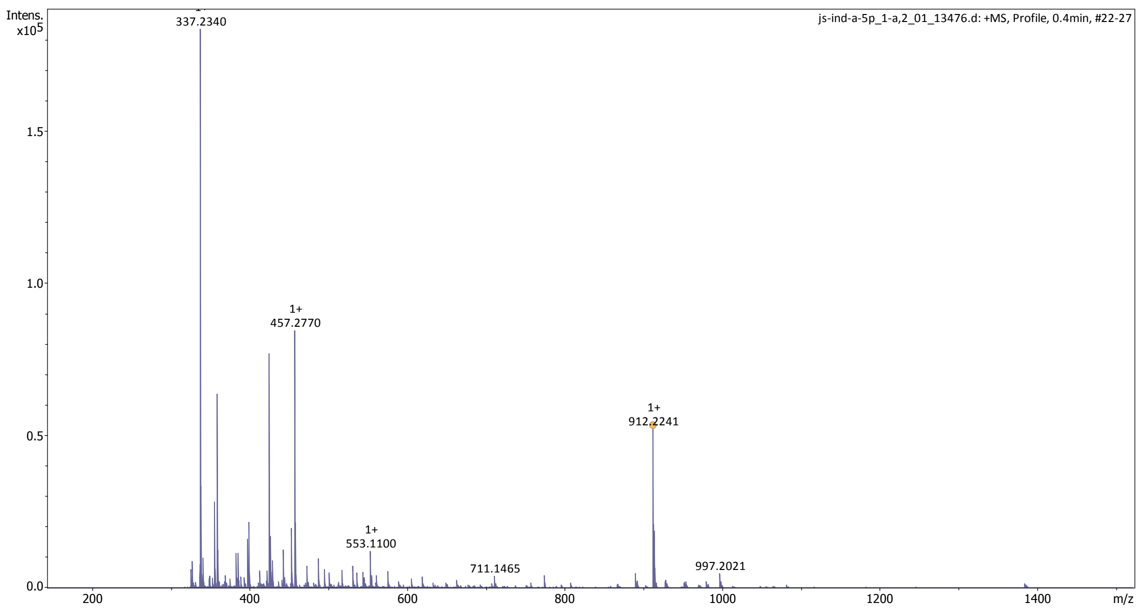

Figure S56. ESI-HRMS of complex **4.**

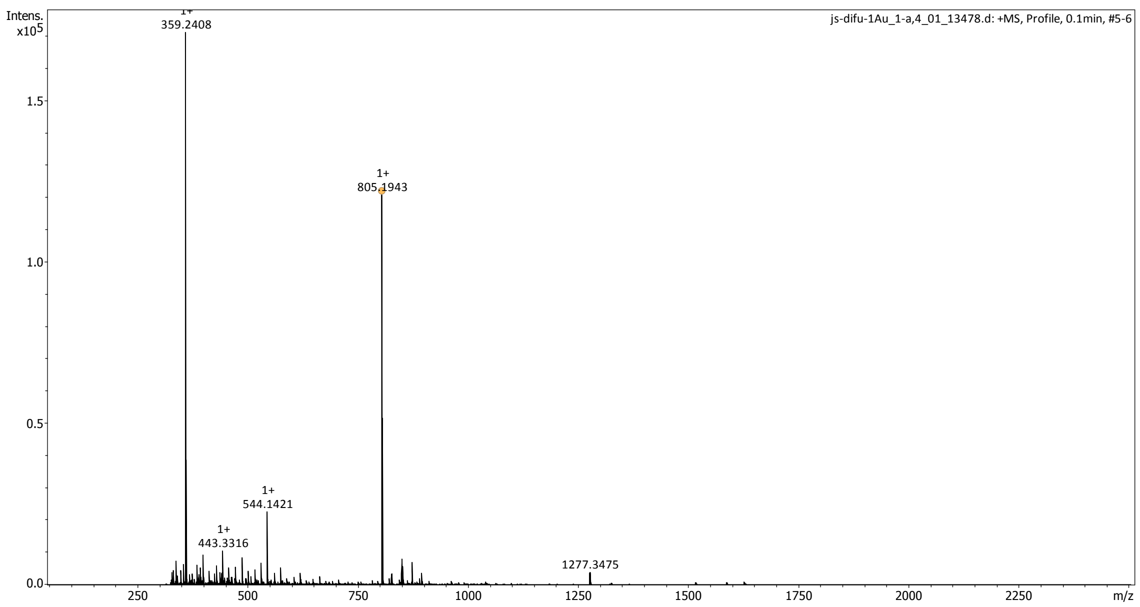

Figure S57. ESI-HRMS of complex **5.**

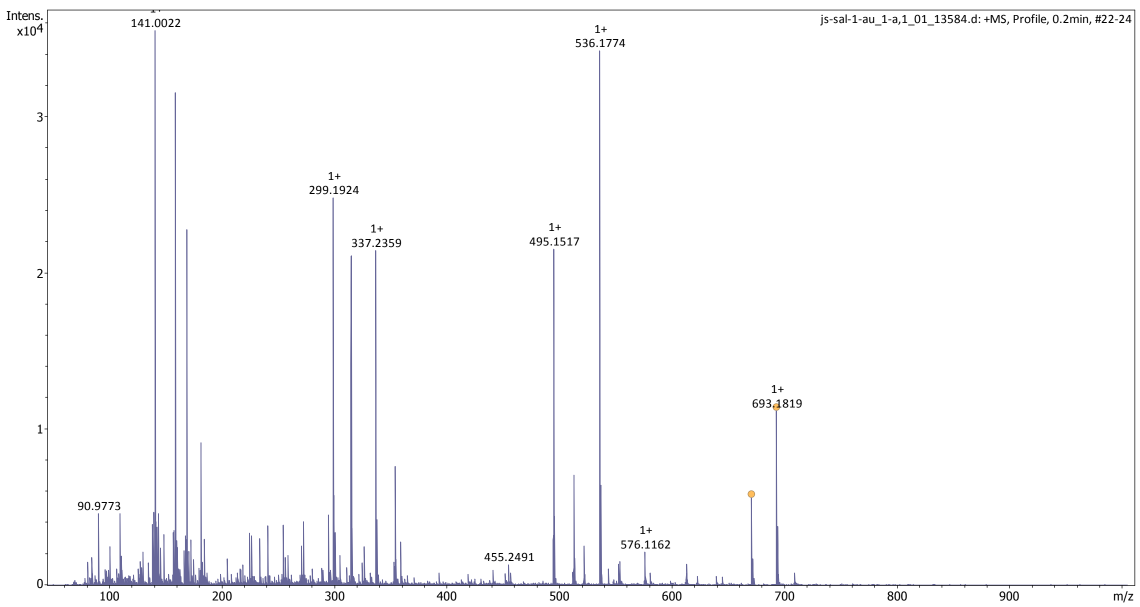

Figure S58. ESI-HRMS of complex **6.**

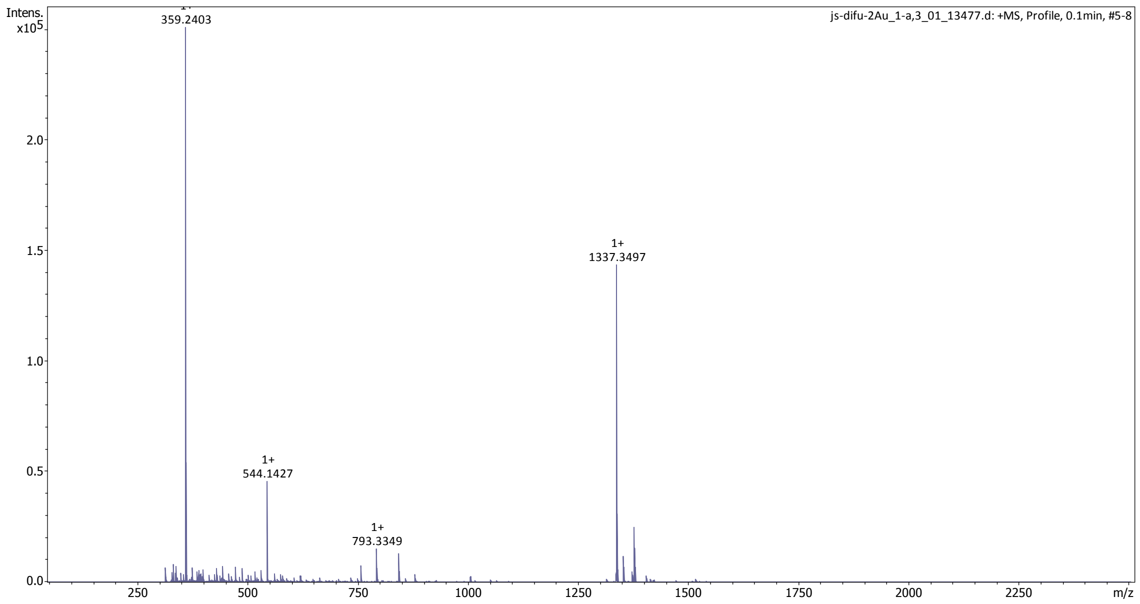

Figure S59. ESI-HRMS of complex **7.**

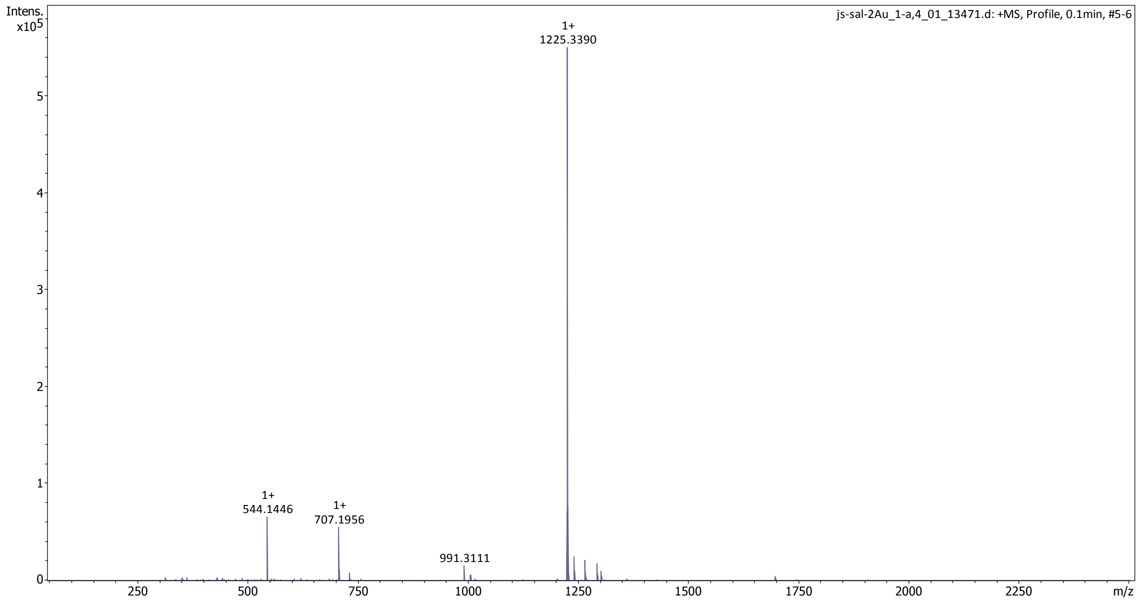

Figure S60. ESI-HRMS of complex **8.**

Table S1. IC_{50} (μM) values of the free ligands on Caco-2/TC7, MCF-7 and MDA-MB-231 cancer cell lines

	IC_{50} (μM)		
Ligand	Caco-2/TC7	MCF-7	MDA-MB231
**L1**	>70	>60	>70
**L2**	>70	>70	>60
**L3**	>80	>90	>70
**L4**	>60	>80	>60
**L5**	>100	>90	>80
**L6**	>100	>90	>80
**L7**	>50	>60	>70
**L8**	>90	>80	>90

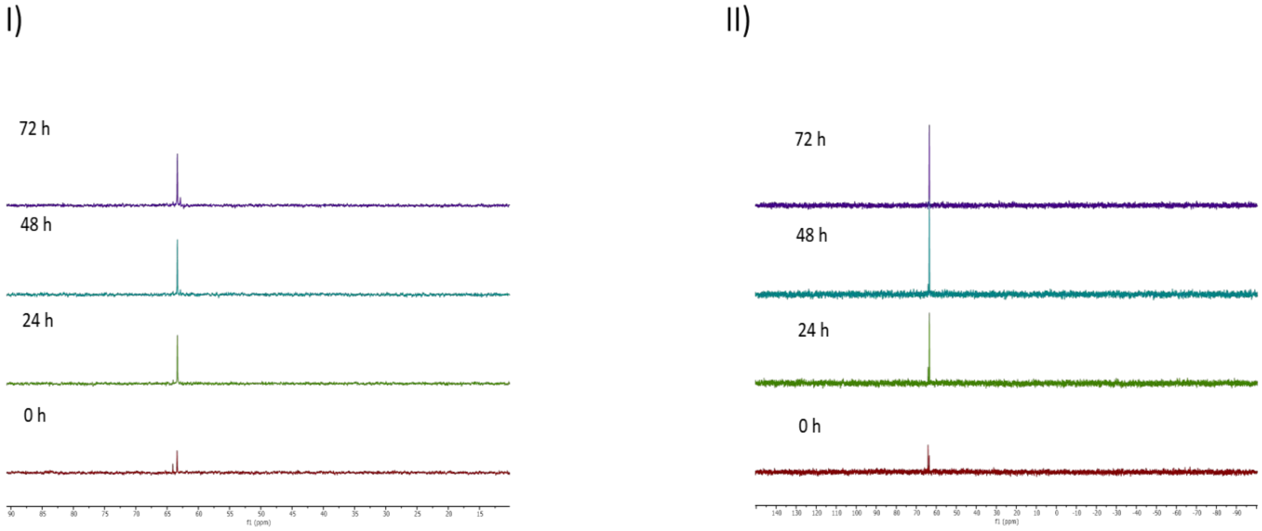

Figure S61. I) $^{31}P\{^{1}H\}$ NMR spectrum in DMSO-d_6/D_2O (80:20, v/v) enlarged at 72h of complex 1 + NAC. II) $^{31}P\{^{1}H\}$ NMR spectrum enlarged at 72h of complex 1 + GSH.

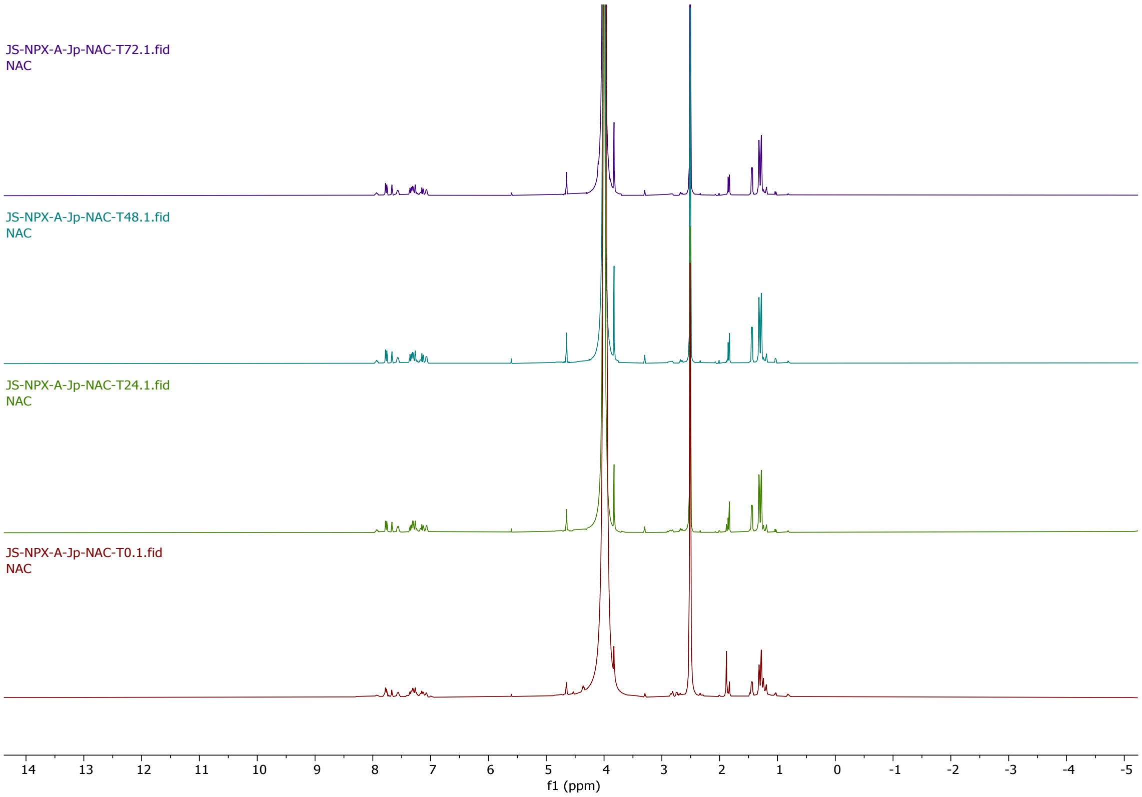

Figure S62. ^{1}H NMR spectrum in DMSO-d_6/D_2O (80:20, v/v) of complex **1** + NAC.

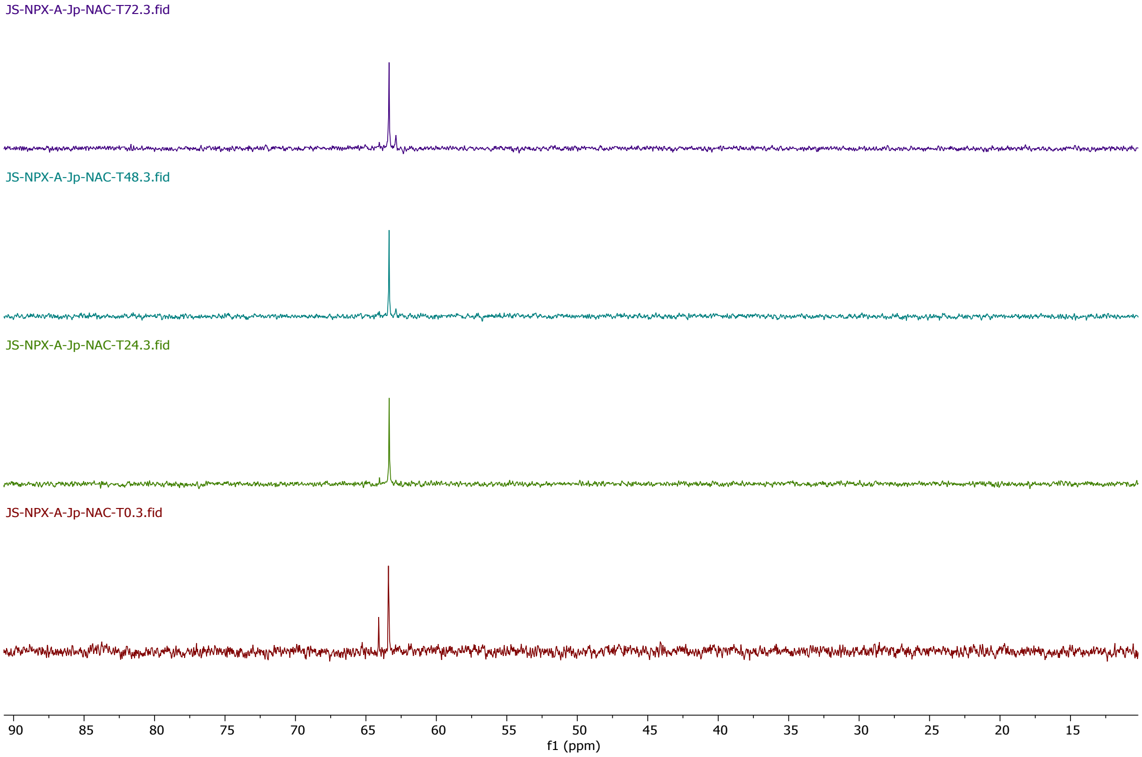

Figure S63. $^{31}P\{^{1}H\}$ NMR spectrum in DMSO-d_6/D_2O (80:20, v/v) of complex **1** + NAC.

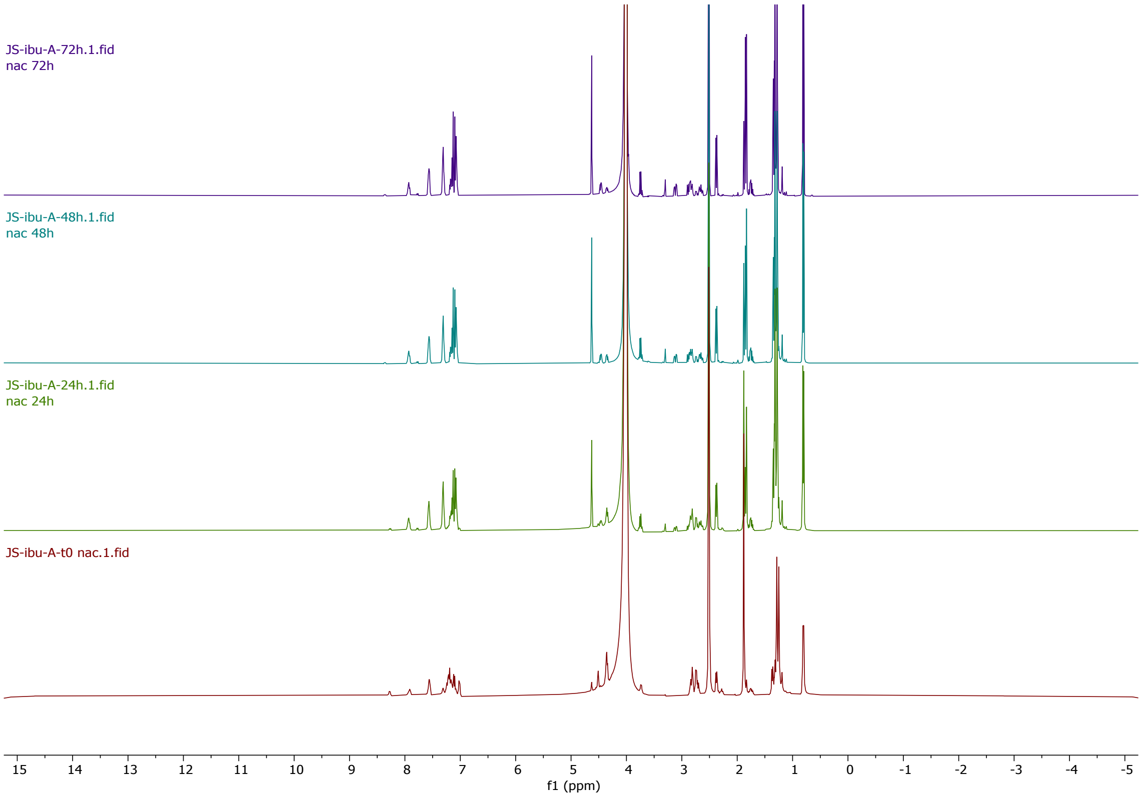

Figure S 64. ^{1}H NMR spectrum in DMSO-d_6/D_2O (80:20, v/v) of complex **2** + NAC.

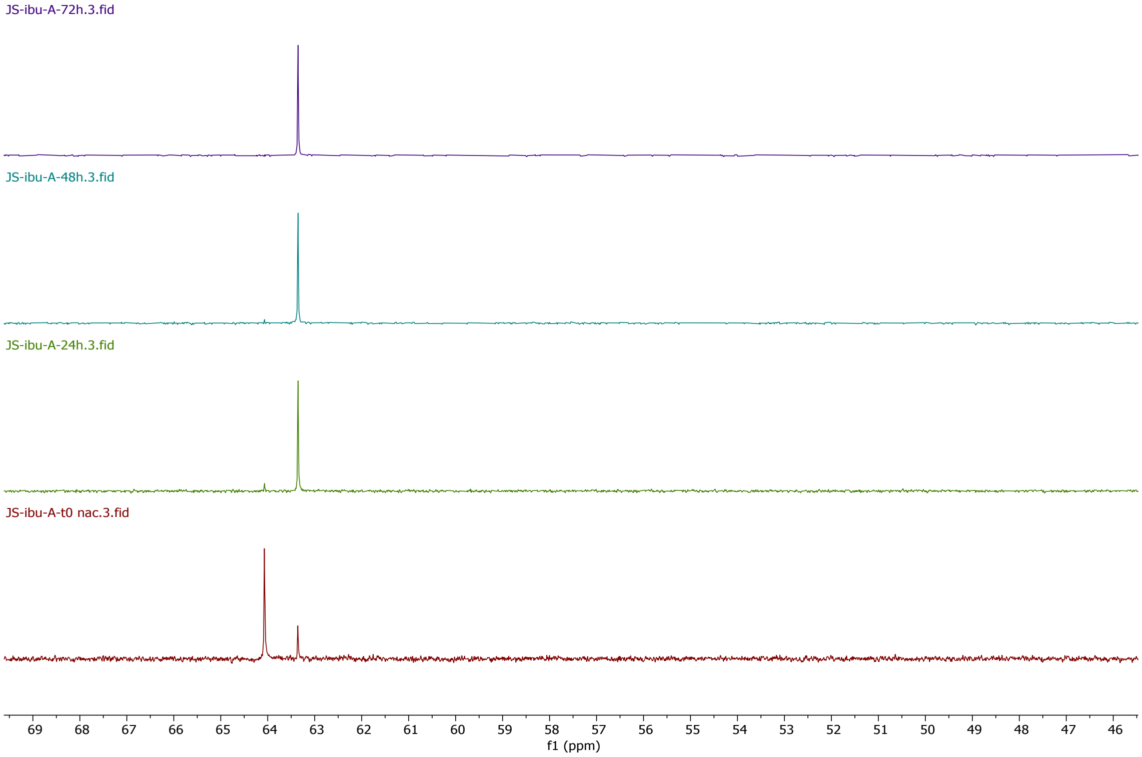

Figure S65. $^{31}P\{^{1}H\}$ NMR spectrum in DMSO-d_6/D_2O (80:20, v/v) of complex **2** + NAC.

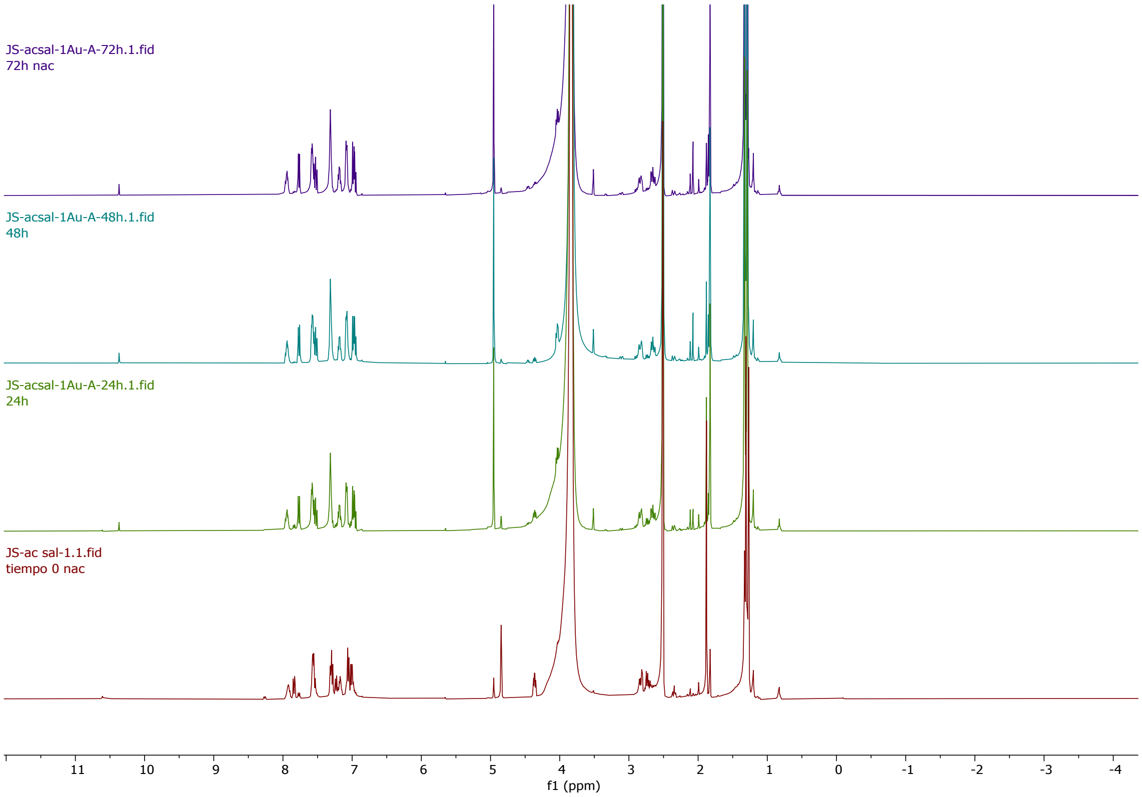

Figure S66. ^{1}H NMR spectrum in DMSO-d_6/D_2O (80:20, v/v) of complex **6** + NAC.

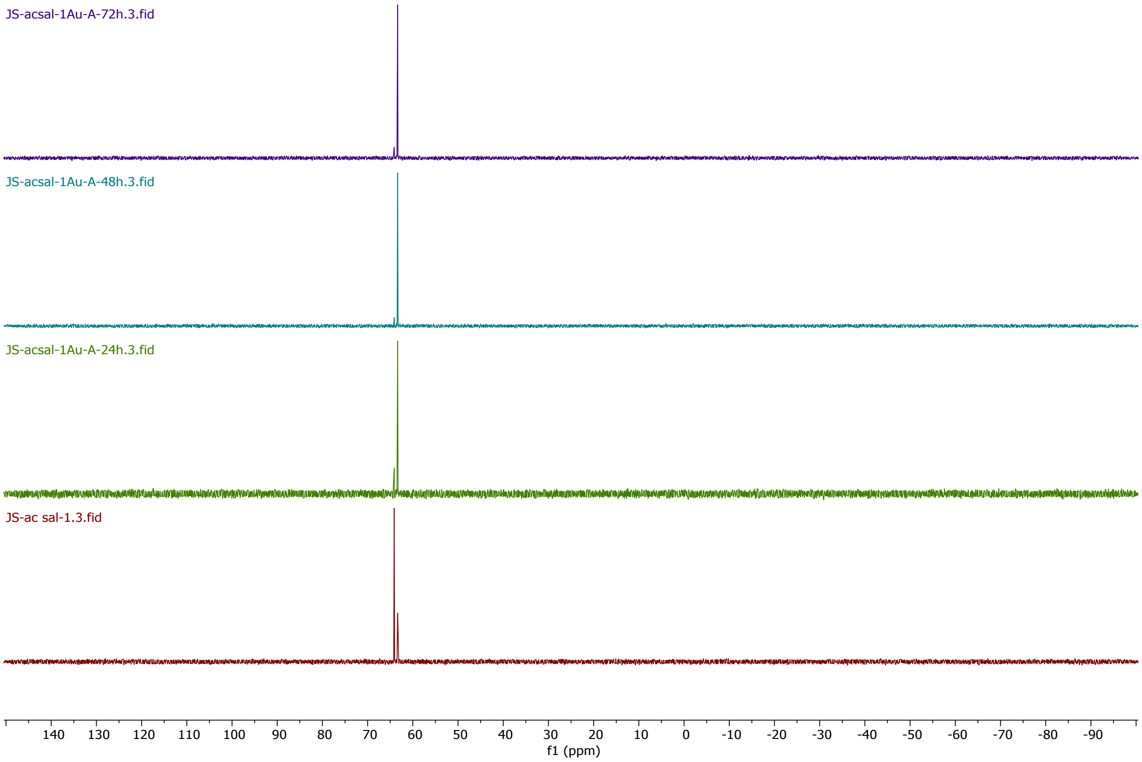

Figure S67. $^{31}P\{^{1}H\}$ NMR spectrum in DMSO-d_6/D_2O (80:20, v/v) of complex **6** + NAC.

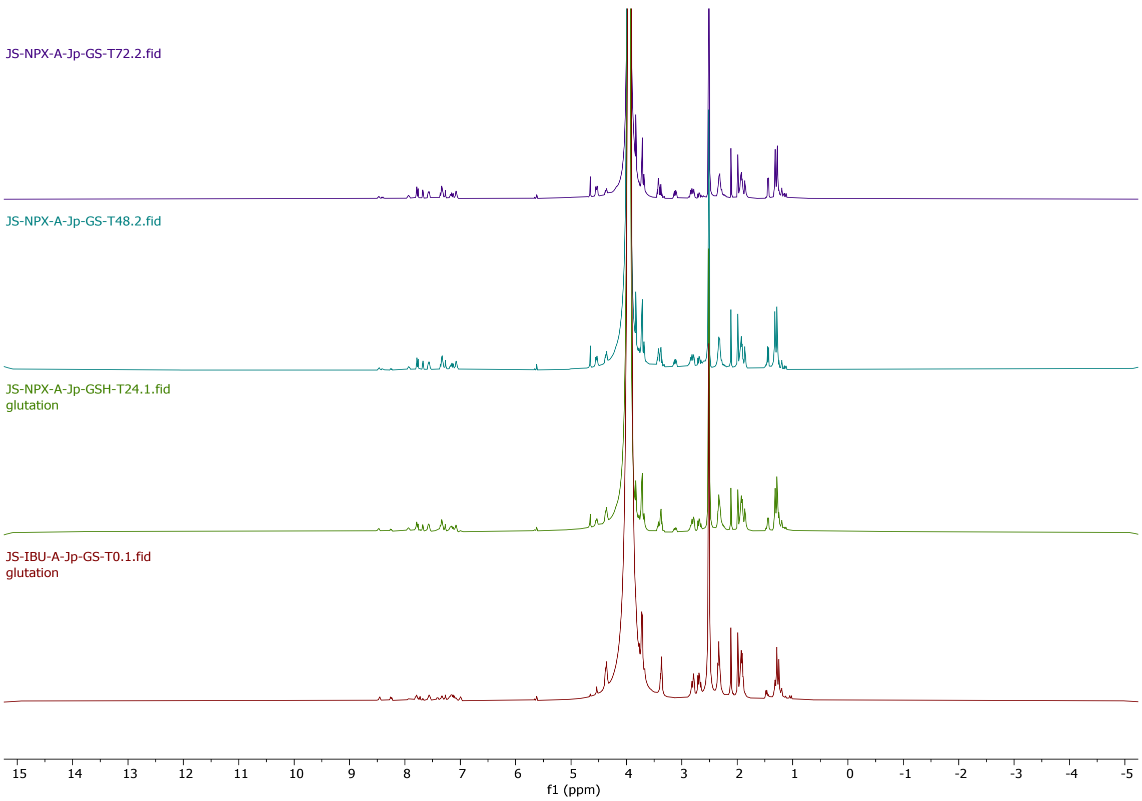

Figure S68.^{1}H NMR spectrum in DMSO-d_6/D_2O (80:20, v/v) of complex **1** + GSH.

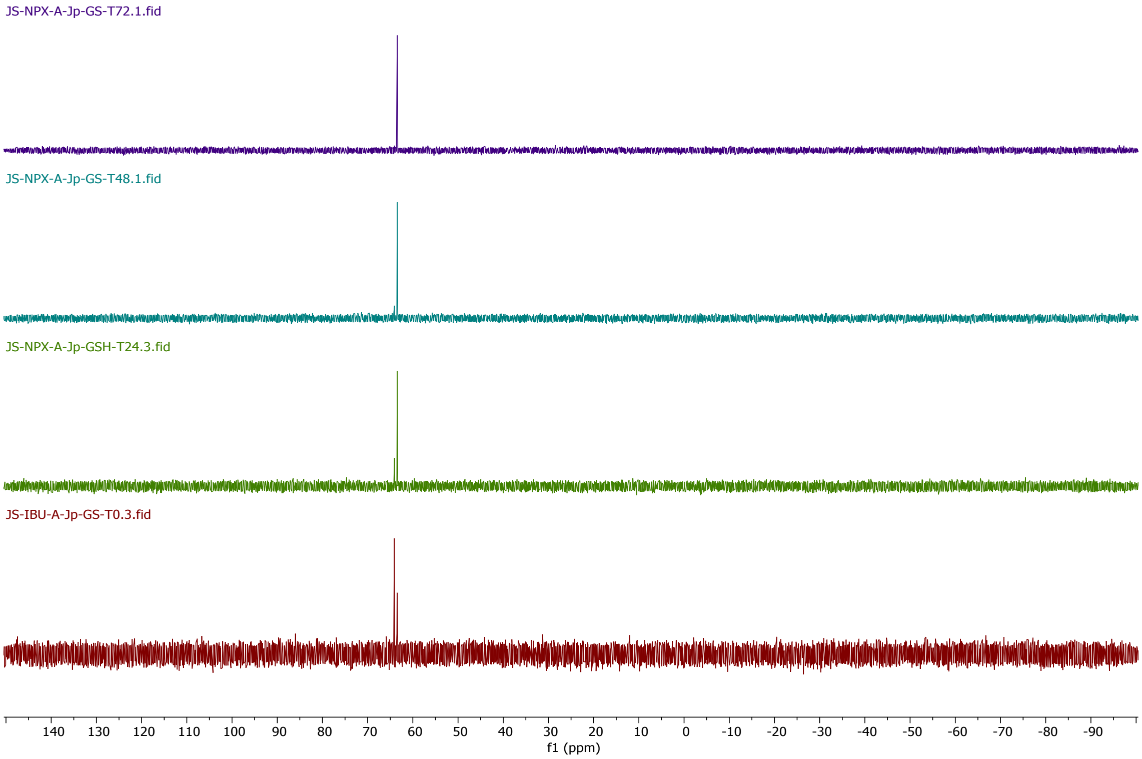

Figure S69. $^{31}P\{^1H\}$ NMR spectrum in DMSO-d_6/D_2O (80:20, v/v) of complex **1** + GSH.

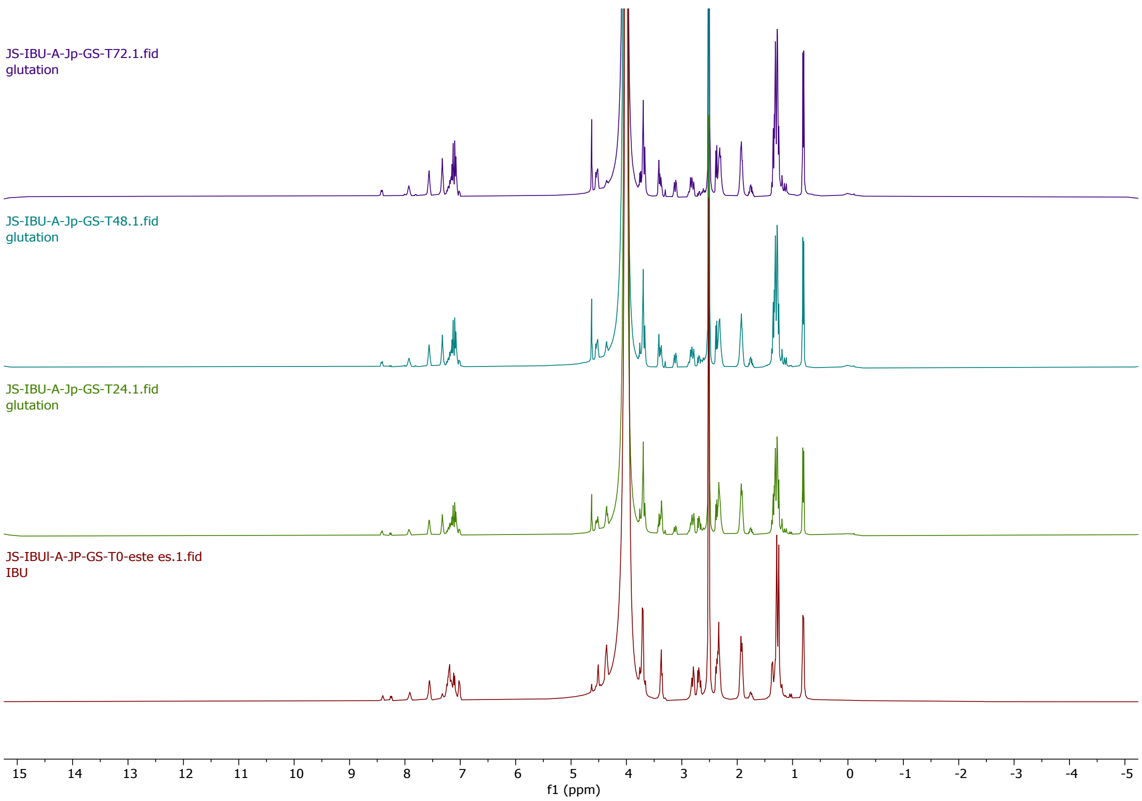

Figure S70. ^{1}H NMR spectrum in DMSO-d_6/D_2O (80:20, v/v) of complex **2** + GSH.

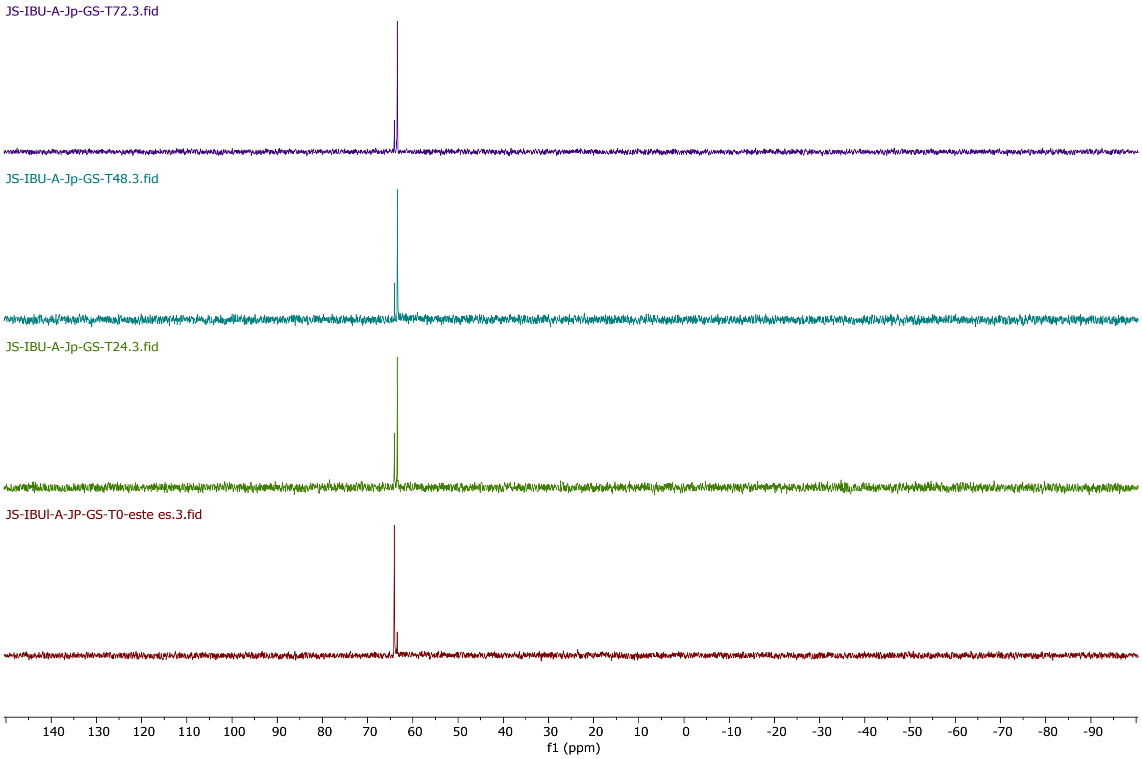

Figure S71. $^{31}P\{^{1}H\}$ NMR spectrum in DMSO-d_6/D_2O (80:20, v/v) of complex **2** + GSH.

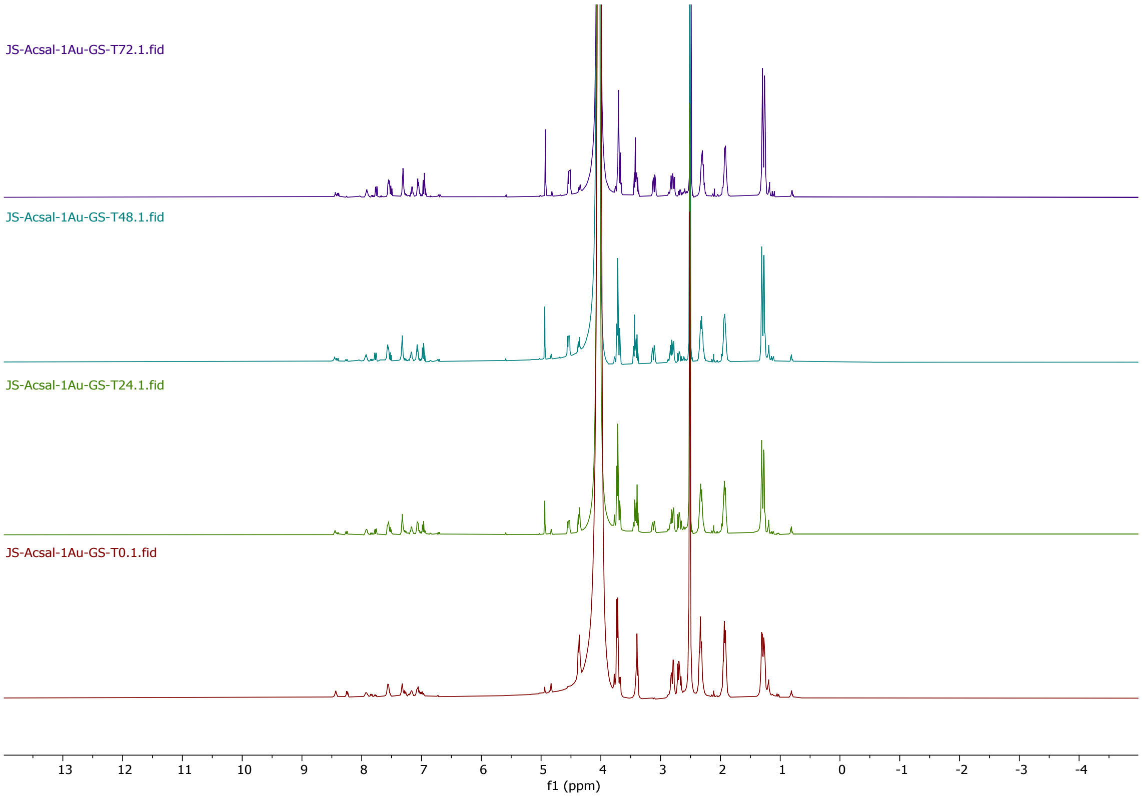

Figure S72. ^{1}H NMR spectrum in DMSO-d_6/D_2O (80:20, v/v) of complex **6** + GSH.

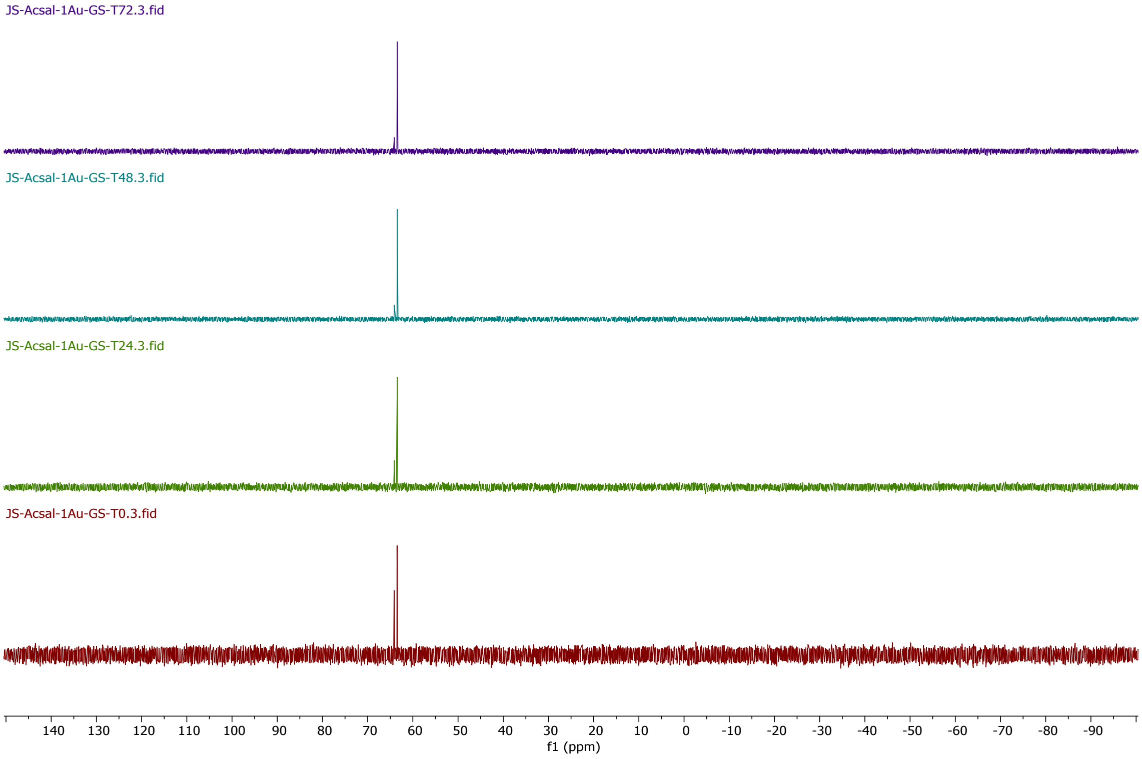

Figure S73. $^{31}P\{^{1}H\}$ NMR spectrum in DMSO-d_6/D_2O (80:20, v/v) of complex **6** + GSH.

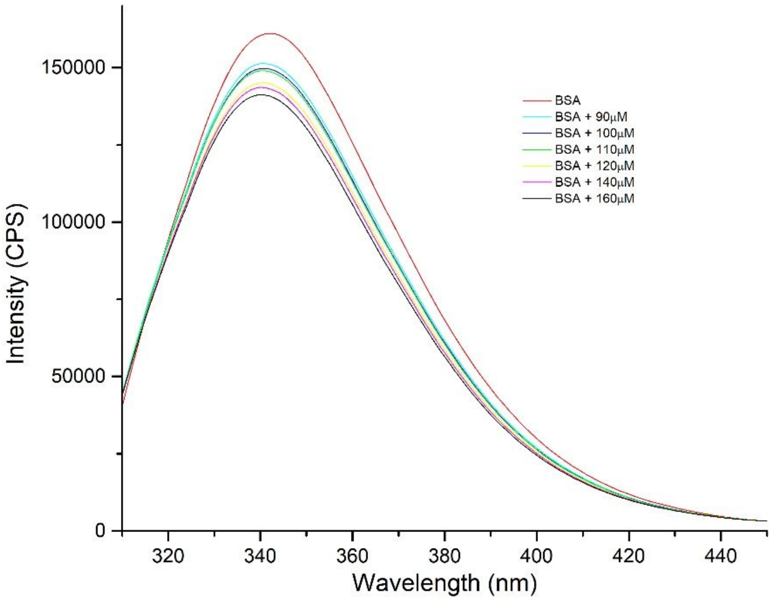

Figure S74. Fluorescence emission spectra of BSA at 298 K in the presence of increasing amounts of complex **2.**

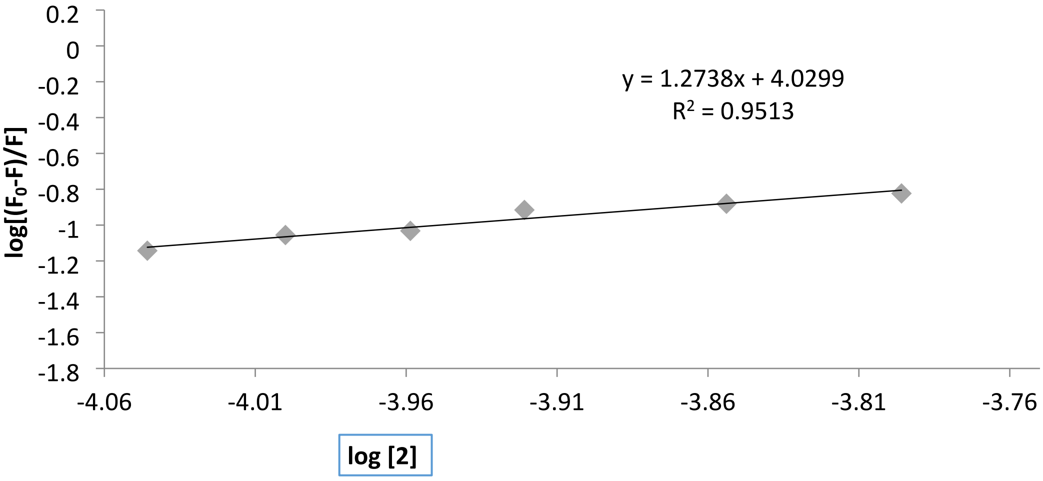

Figure S75. Stern-Volmer equation used: $\log\{(F_0\text{-}F)/F\} = \log K_b + n\log[\mathbf{2}]$. The intercept of the best fit linear trend provides the Stern-Volmer quenching constant K_b.

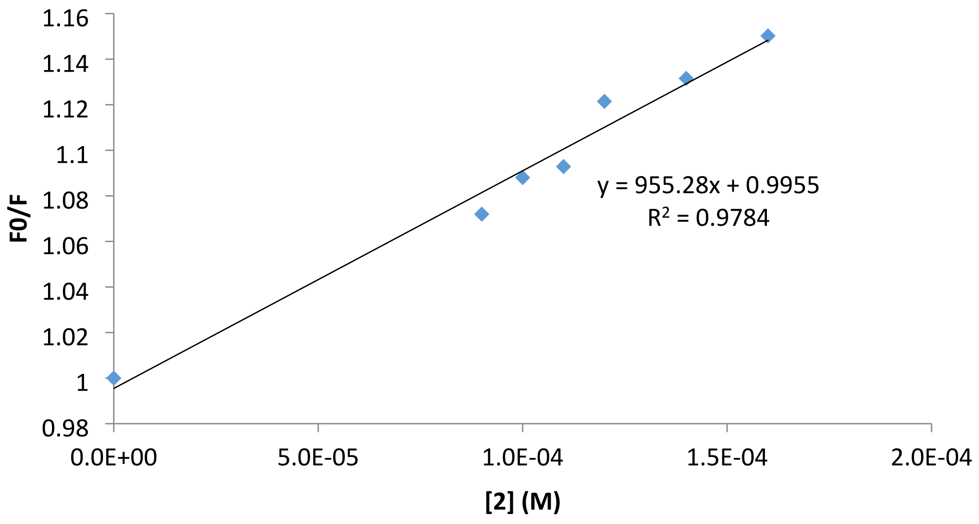

Figure S76. Stern-Volmer plot for the quenching of BSA with complex **2.**

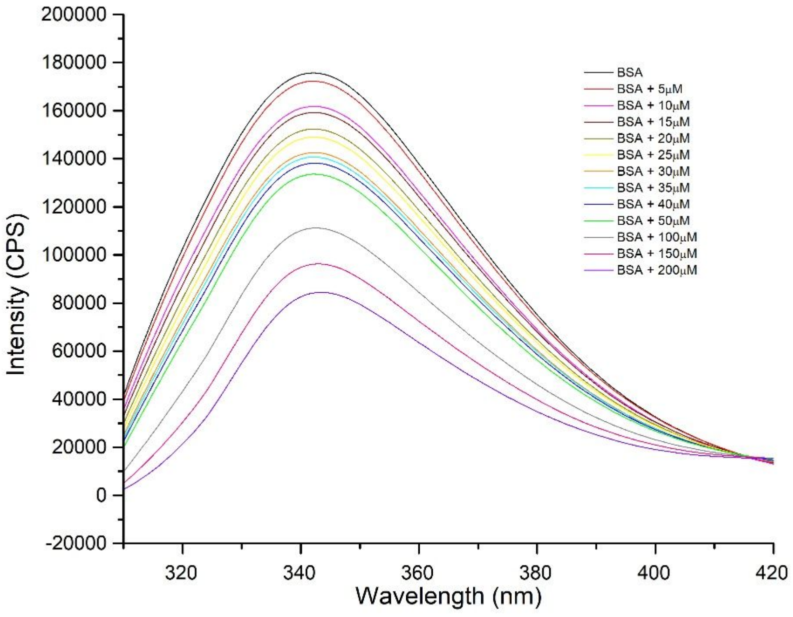

Figure S77. Fluorescence emission spectra of BSA at 298 K in the presence of increasing amounts of complex **6.**

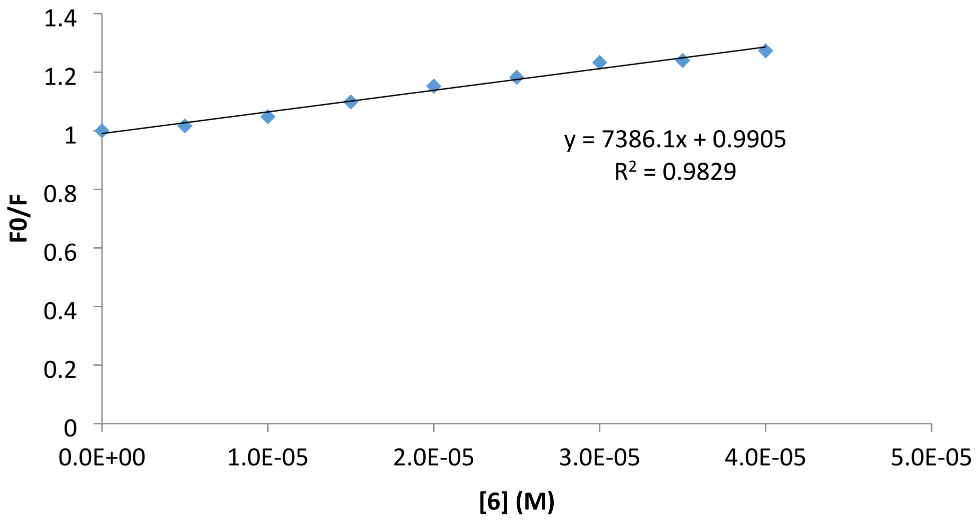

Figure S78. Stern-Volmer plot for the quenching of BSA with complex **6.**

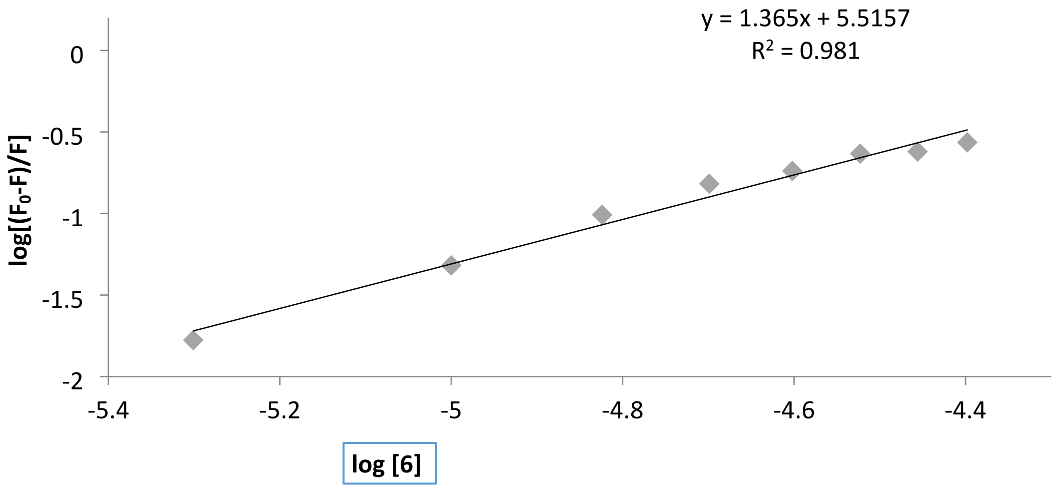

Figure S79. Stern-Volmer equation used: log{(F_0-F)/F} = logK_b + $n$log[**6**]. The intercept of the best-fit linear trend provides the Stern-Volmer quenching constant K_b.

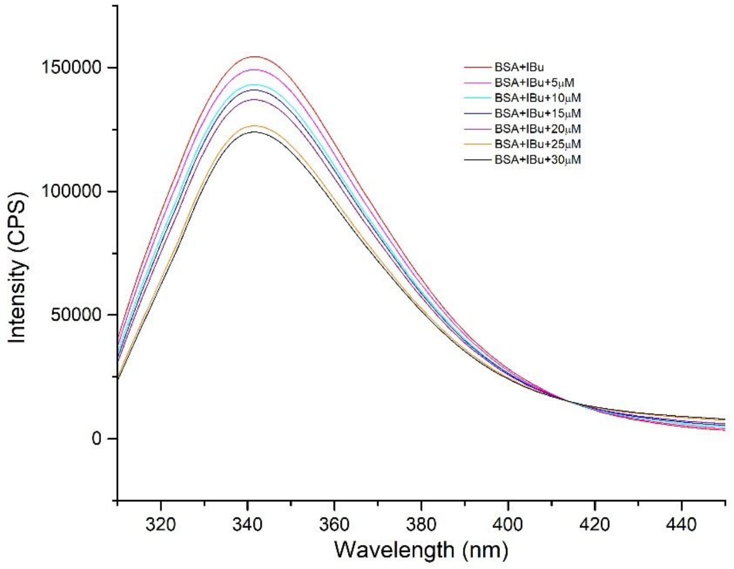

Figure S80. Fluorescence emission spectra of BSA with Ibuprofen at 298 K in the presence of increasing amounts of **6**

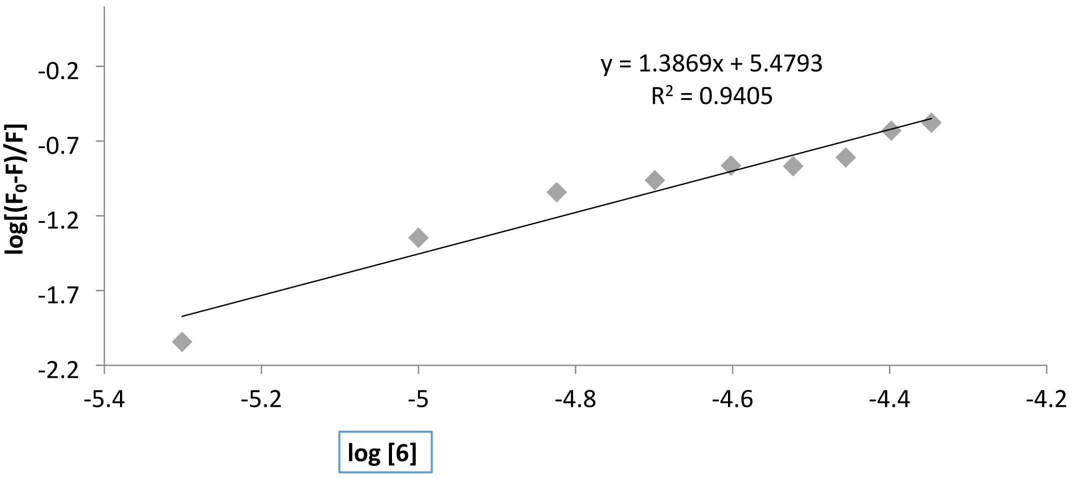

Figure S81. BSA+Ibuprofen: Stern-Volmer equation used: $\log\{(F_0\text{-}F)/F\} = \log K_b + n\log[\mathbf{6}]$. The intercept of the best-fit linear trend provides the Stern-Volmer quenching constant K_b.

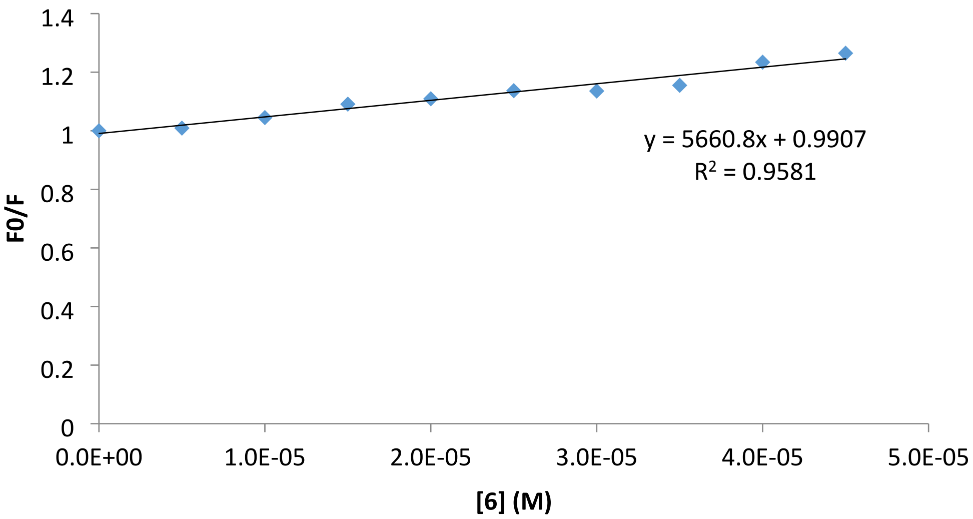

Figure S82. Stern-Volmer plot for the quenching of BSA+Ibuprofen with complex **6.**

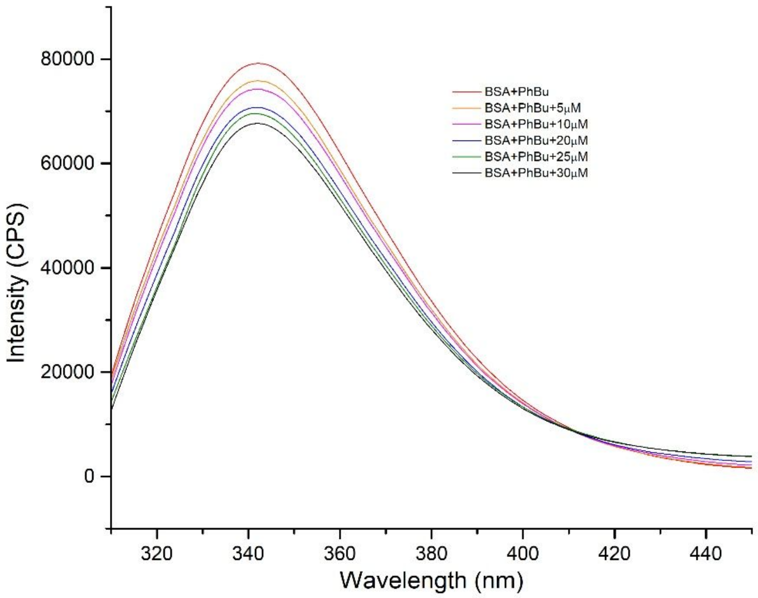

Figure S83. Fluorescence emission spectra of BSA with phenylbutazone at 298 K in the presence of increasing amounts of **6.**

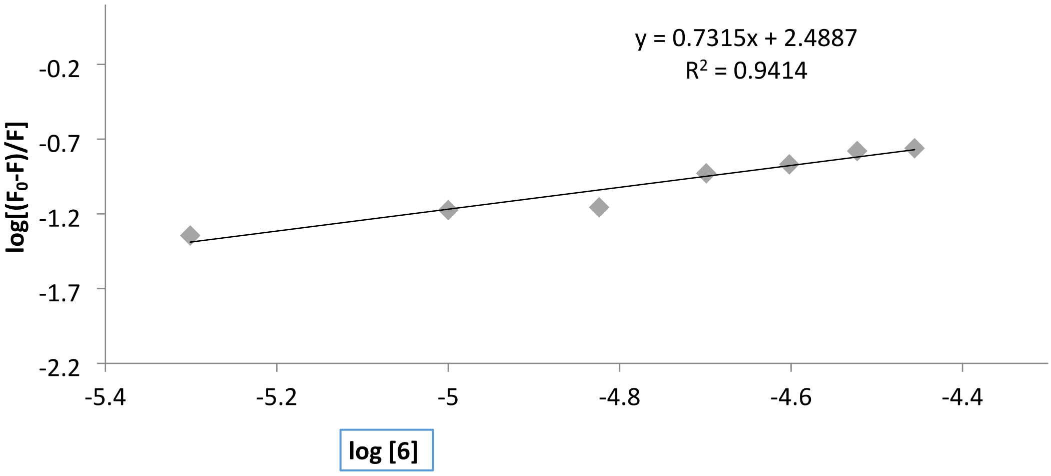

Figure S84. BSA+phenylbutazone: Stern-Volmer equation used: log{(F_0-F)/F} = logK_b + $n$log[**6**]. The intercept of the best fit linear trend provides the Stern-Volmer quenching constant K_b.

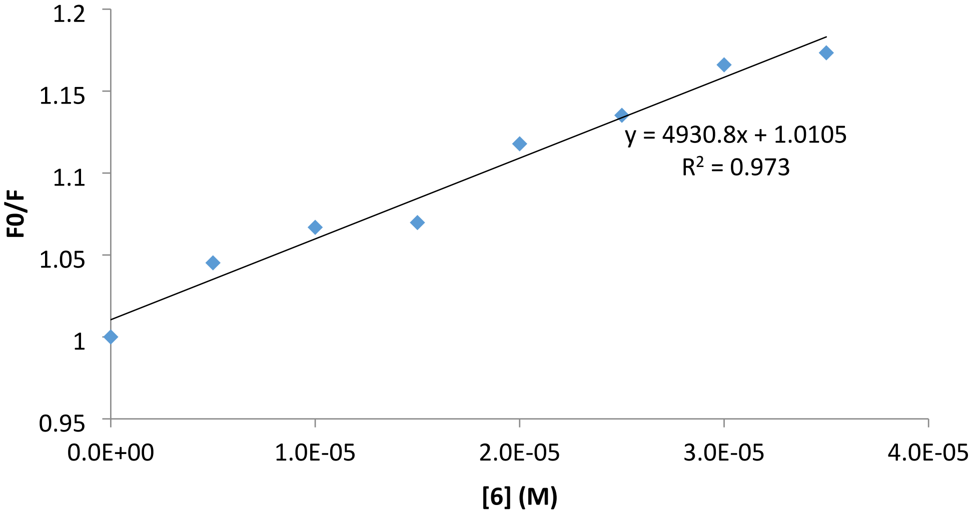

Figure S85. Fluorescence emission spectra of BSA with phenylbutazone at 298 K in the presence of increasing amounts of **6.**

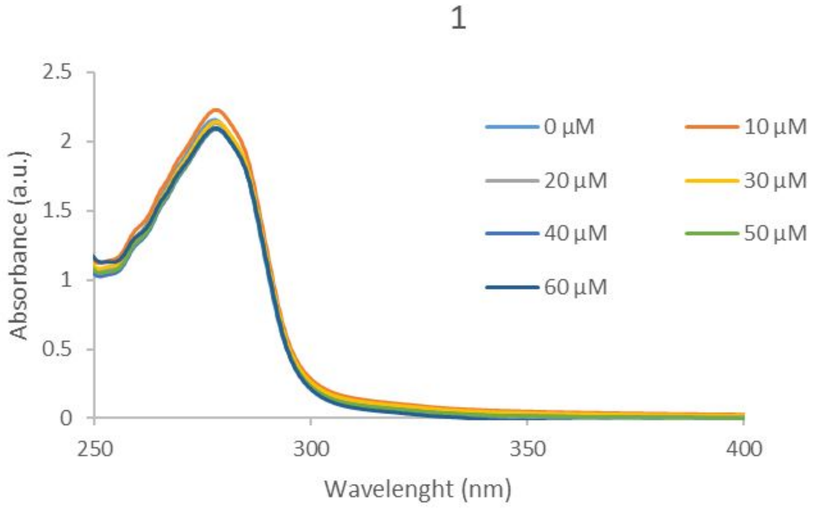

Figure S86. UV-visible absorption spectra of BSA with increasing amounts of complex **1** at 298K. [BSA = 10 μM] and [complex] (10-50μM). An equal concentration of the complex was also added to the reference cell (in each measurement) to eliminate its absorbance.

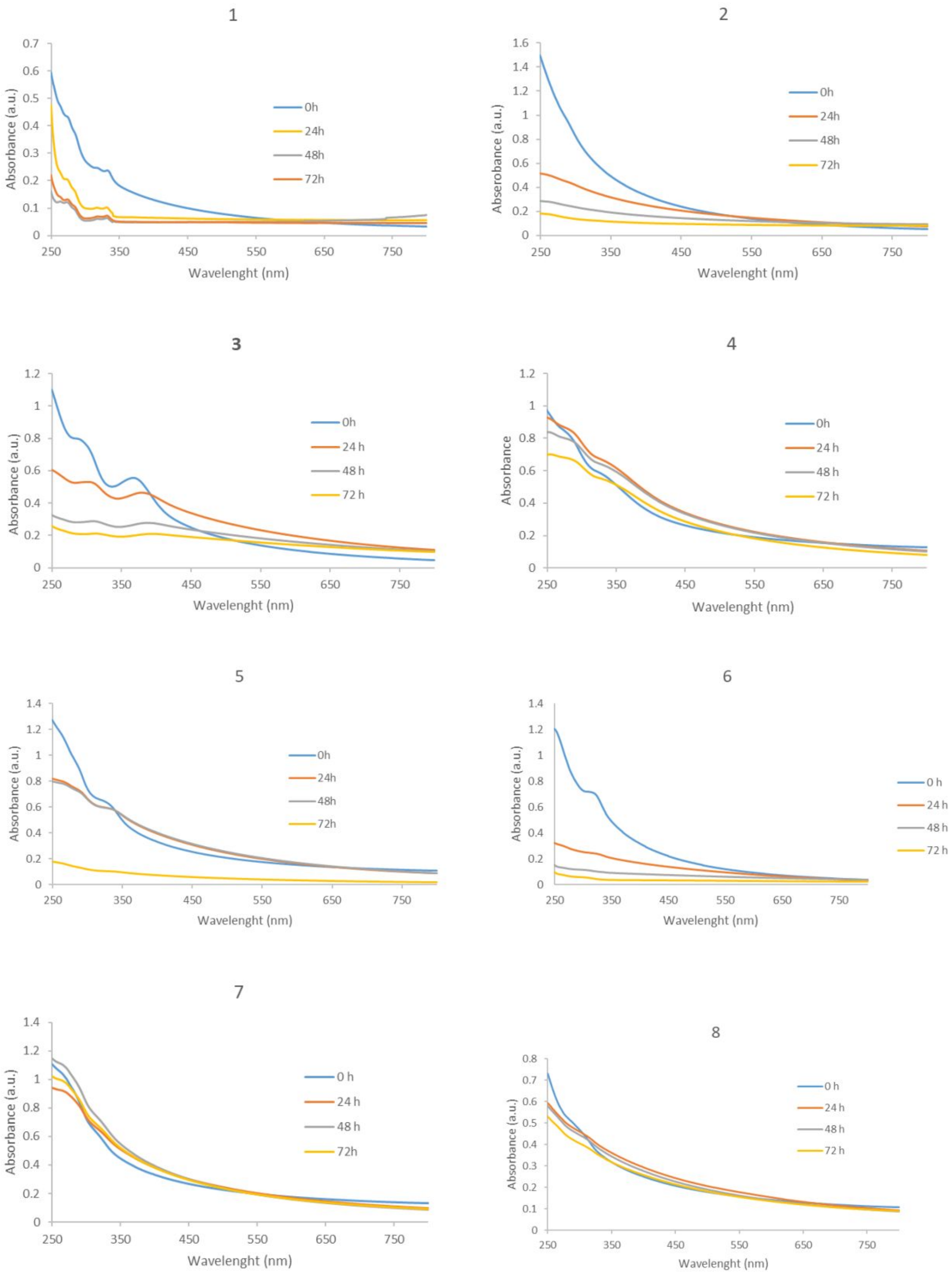

Figure S87. UV-Vis spectra of the complexes over 72 hours.

Table S2. Characteristics of primers used in RT-qPCR according to MIQE guidelines.

Gene symbol	Accession	Sequence (5' → 3')		Amplicon length (nt)	Exons	[Primer] nM	Efficiency %
GAPDH	NM_002046.7	Sense<br>Antisense	CATGTTCGTCATGGGTGTGAACCA<br>AGTGATGGCATGGACTGTGGTCAT	137	6-7	150	95
IL6	NM_000600.5	Sense<br>Antisense	TTCGGTCCAGTTGCCTTCTC<br>CAGCTCTGGCTTGTTCCTCA	403	2-4	-	-
IL8	NM_000584.4	Sense<br>Antisense	CCAGGAAGAAACCACCGGAA<br>TTCTCAGCCCTCTTCAAAAACT	337	1-4	80	95
NOS2	NM_000625.4	Sense<br>Antisense	CCCGAGTCAGAGTCACCATC<br>GGCAGCTCAGCCTGTACTTA	135	14-15	100	96
PTGS2	NM_000963.4	Sense<br>Antisense	ACCCACTCCAAACACAGTGC<br>AAGGGAGTCGGGCAATCATC	243	3-4	100	93

References

1. Martínez-Beamonte, R.; Navarro, M. A.; Larraga, A.; Strunk, M.; Barranquero, C.; Acín, S.; Guzman, M. A.; Iñigo, P.; Osada, J. Selection of reference genes for gene expression studies in rats. *J. Biotechnol.* **2011,** *151* (4), 325-34.

2. G. M. Sheldrick, *SADABS, Program for adsorption correction*. University of Göttingen, Göttingen, Germany, 1996.

3. Sheldrick, G. Crystal structure refinement with SHELXL. *Acta Cryst. Section C* **2015,** *71* (1), 3-8.
